# A genome-wide CRISPR-Cas9 knockout screen identifies essential and growth-restricting genes in human trophoblast stem cells

**DOI:** 10.1038/s41467-022-30207-9

**Published:** 2022-05-10

**Authors:** Chen Dong, Shuhua Fu, Rowan M. Karvas, Brian Chew, Laura A. Fischer, Xiaoyun Xing, Jessica K. Harrison, Pooja Popli, Ramakrishna Kommagani, Ting Wang, Bo Zhang, Thorold W. Theunissen

**Affiliations:** 1 Department of Developmental Biology, Washington University School of Medicine, St. Louis, MO 63110 USA; 2 Center of Regenerative Medicine, Washington University School of Medicine, St. Louis, MO 63110 USA; 3 Department of Genetics, Center for Genome Sciences & Systems Biology, Washington University School of Medicine, St. Louis, MO 63110 USA; 4 Department of Obstetrics and Gynecology, Center for Reproductive Health Sciences, Washington University School of Medicine, St. Louis, MO 63110 USA

**Keywords:** CRISPR-Cas systems, Self-renewal

## Abstract

The recent derivation of human trophoblast stem cells (hTSCs) provides a scalable in vitro model system of human placental development, but the molecular regulators of hTSC identity have not been systematically explored thus far. Here, we utilize a genome-wide CRISPR-Cas9 knockout screen to comprehensively identify essential and growth-restricting genes in hTSCs. By cross-referencing our data to those from similar genetic screens performed in other cell types, as well as gene expression data from early human embryos, we define hTSC-specific and -enriched regulators. These include both well-established and previously uncharacterized trophoblast regulators, such as *ARID3A, GATA2*, and *TEAD1* (essential), and *GCM1, PTPN14*, and *TET2* (growth-restricting). Integrated analysis of chromatin accessibility, gene expression, and genome-wide location data reveals that the transcription factor TEAD1 regulates the expression of many trophoblast regulators in hTSCs. In the absence of *TEAD1*, hTSCs fail to complete faithful differentiation into extravillous trophoblast (EVT) cells and instead show a bias towards syncytiotrophoblast (STB) differentiation, thus indicating that this transcription factor safeguards the bipotent lineage potential of hTSCs. Overall, our study provides a valuable resource for dissecting the molecular regulation of human placental development and diseases.

## Abstract (web summary)

Here the authors perform a genome-wide CRISPR-Cas9 knockout screen to systematically identify and characterize essential and growth-restricting genes in human trophoblast cells. They identify TEAD1 as a key regulator that plays an important role in the specification, maintenance, and differentiation of the human trophoblast lineage by modulating chromatin architecture and gene expression.

## Introduction

The placenta is a transient organ containing trophoblast cells derived from the trophectoderm (TE) of the early embryo^1^. It performs several critical functions throughout gestation, including facilitating maternal-fetal exchanges, remodeling maternal spiral arteries, secreting pregnancy-related hormones, and acting as a protective barrier^2–4^. Indeed, trophoblast defects can lead to pathologies such as miscarriage, preeclampsia, and intrauterine growth restrictions^5–7^. However, the placenta also remains one of the least understood organs. Recently, a culture condition that allows for the derivation and maintenance of bona fide hTSCs from the blastocyst or first trimester placenta was developed^8^, which provides an invaluable tool to study human trophoblast development. Subsequent studies from our laboratory and others showed that hTSCs with similar biological and molecular properties can also be derived from naïve hPSCs or somatic cells via direct reprogramming^9–13^. Nevertheless, efforts to systematically identify crucial regulators of hTSCs have so far been lacking.

Here, we employ a pooled genome-wide CRISPR-Cas9 knockout screen, an increasingly powerful tool to uncover and characterize genes controlling a variety of biological processes^14–22^, to comprehensively determine the essential genes (EGs) and growth-restricting genes (GRGs) of hTSCs. Using a combination of chromatin accessibility, gene expression, and genome-wide location analyses, we further show that one such regulator, the transcription factor *TEAD1*, plays major role during progressive stages of human trophoblast development. Overall, this work provides important insights into the regulation of hTSCs and will be a valuable resource for future studies of trophoblast biology.

## Results and discussion

### A genome-wide CRISPR-Cas9 knockout screen identifies hTSC EGs

**Fig. 1:**
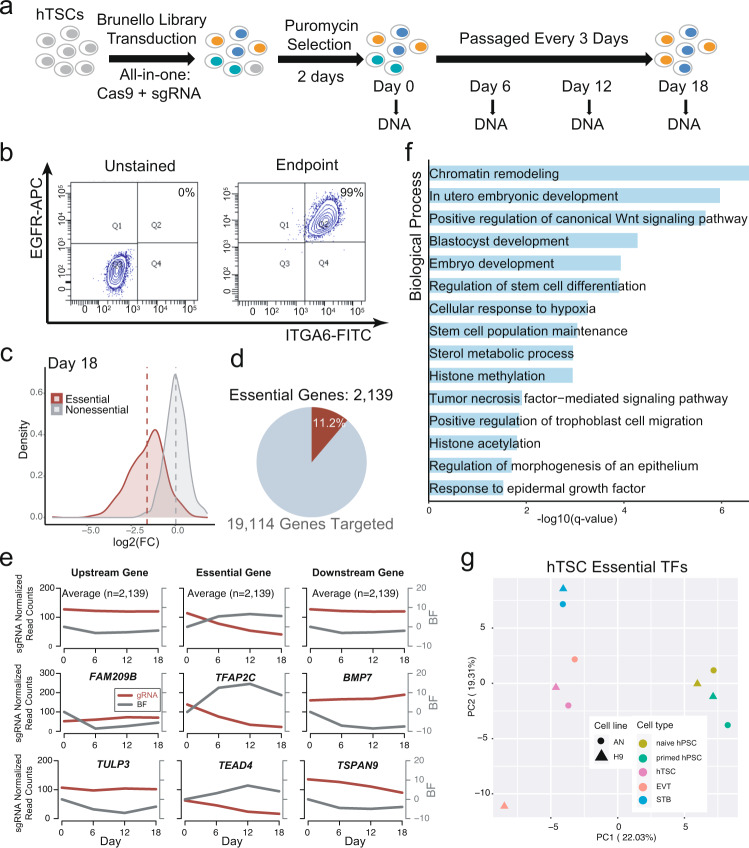
A genome-wide CRISPR-Cas9 knockout screen identifies hTSC EGs. **a** The experimental scheme of the CRISPR screen. **b** Flow cytometry analysis for hTSC markers ITGA6 and EGFR in BT5 hTSCs following the CRISPR screen endpoint. **c** Fold change distribution of sgRNAs targeting core essential and nonessential genes^26^ at day 18 of the screen. Note that the Log2 fold change (L2FC) of the core EGs gradually decreased relative to day 6 and day 12, while the L2FC of the nonessential genes largely remained unchanged across different timepoints. **d** Percentage of essential genes (EGs) identified among all targeted genes. **e** The mean of sgRNA normalized read counts and Bayes Factors (BFs) of all or selected hTSC EGs and their neighboring up- and downstream genes over time. The results are representative of two independently transduced screening experiments. **f** Selected GO Biological Processes terms that are significantly enriched among all hTSC EGs. **g** PCA featuring published AN and H9 primed hPSCs, naïve hPSCs, naïve hTSCs, EVT, and STB RNA-seq samples^10^ using the gene expression data of hTSC essential transcription factors^10^.

We transduced the blastocyst-derived BT5 hTSC line^8^ with the Brunello human CRISPR knockout pooled lentiviral library, which contains 76,441 single guide RNAs (sgRNAs) targeting 19,114 genes^23^. Following puromycin selection, the cells were cultured and passaged for 18 days, with genomic DNA samples harvested for deep sequencing at days 0, 6, 12, and 18 (Fig. 1a). Two replicates with independent transductions showed good reproducibility (Supplementary Fig. 1a) and the identity of the hTSCs at day 18 was confirmed by homogeneous expression of the hTSC-specific cell surface markers EGFR and ITGA6 (Fig. 1b and Supplementary Fig. 1b)^10^. We analyzed the enrichment of sgRNAs targeting reference essential and non-essential genes and calculated a precision-recall curve, which indicated that the screen showed excellent performance (Fig. 1c and Supplementary Fig. 1c, d). Furthermore, analysis at intermediate timepoints revealed the lack of a bottlenecking effect (Supplementary Fig. 2a and Supplementary Data 1). In total, the screen identified 2139 EGs (Fig. 1d, Supplementary Fig. 1e, and Supplementary Data 1).

To confirm the specificity of our screen, we plotted the mean normalized sgRNA read counts of all EGs over time together with Bayes Factor (BF), a classifier of gene essentiality^24–26^. As expected, the mean normalized sgRNA reads of all EGs steadily declined while their BF scores increased. In contrast, the gRNA enrichment and BF scores of neighboring genes remained unchanged (Fig. 1e). Amongst the hTSC EGs were several well-established trophoblast regulators, such as *TFAP2C*^10,27–31^ and *TEAD4*^32–35^, as well as genes without a previously reported association with trophoblast, such as *TCAF1*, *USP47*, and *TSC22D2* (Fig. 1e and Supplementary Fig. 3a). Biological processes and pathways enriched among the hTSC EGs included WNT, EGF, and histone acetylation (Fig. 1f and Supplementary Data 2), all of which are modulated by inhibitors or growth factors contained within hTSC medium^8^. We also detected enrichment in components of mTOR signaling and the pentose phosphate pathway (Supplementary Fig. 3b and Supplementary Data 2), which were previously implicated in mouse TE specification^36^.

Consistent with their role in promoting hTSC fitness, EGs identified in our screen were expressed much more abundantly than nonessential genes in hTSCs (Supplementary Fig. 3c). We performed principal component analysis (PCA) using published RNA-sequencing (RNA-seq) data from naïve and primed hPSCs and various trophoblast cell types and found that hTSC EGs (*n* = 2139) and essential TFs (*n* = 124) accurately demarcated pluripotent and trophoblast identities, despite representing only minor fractions of all genes (Fig. 1g, Supplementary Fig. 3d, e, and Supplementary Data 1)^10^. We also compared the expression of hTSC EGs in different trophoblast cell types at 10 and 12 days post fertilization (d.p.f.) in 3D human embryos cultured through implantation stages^37^. According to our prior analysis, these are the stages of early trophoblast development to which hTSCs most closely correspond^10^. At both timepoints, the hTSC EGs were statistically significantly upregulated in the cytotrophoblast (CTB) relative to the terminally differentiated extravillous trophoblasts (EVT) and syncytiotrophoblasts (STB) (Supplementary Fig. 3f, g). Similar expression patterns of hTSC EGs could be observed in trophoblast cells derived in vitro (Supplementary Fig. 3h). These transcriptional data further support the notion that hTSC EGs are central to the identity of hTSCs.

### Identification and characterization of hTSC-specific EGs

**Fig. 2:**
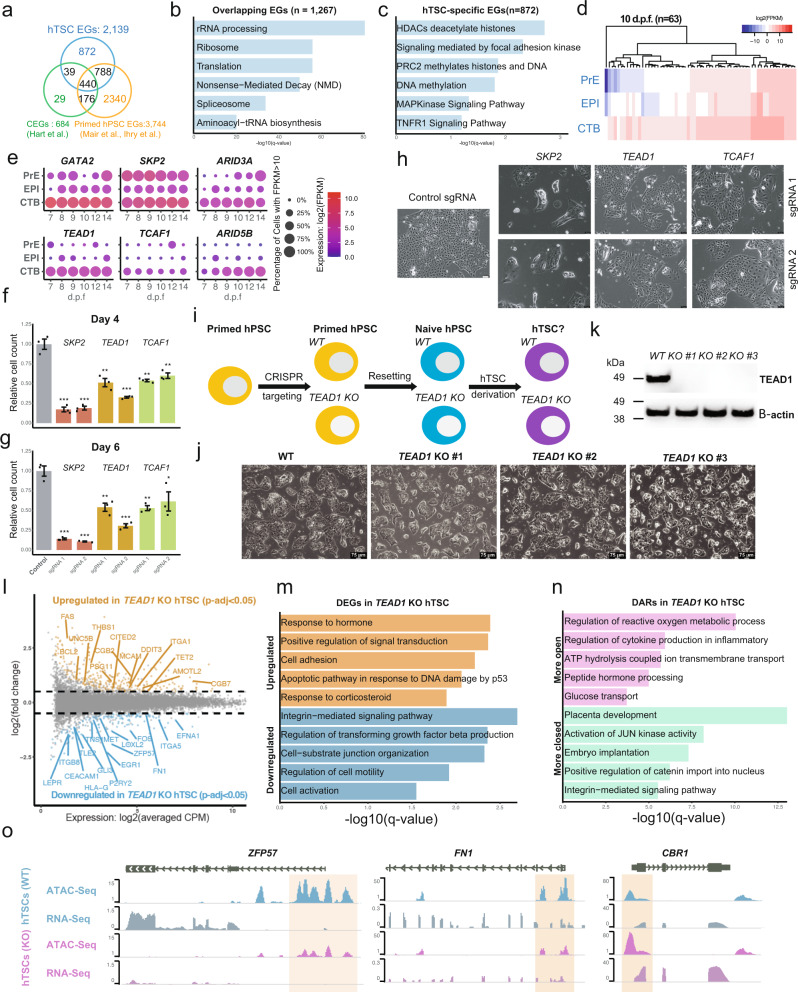
Identification and analysis of hTSC-specific EGs. **a** Overlap of hTSC EGs with core^26^ and primed hPSC EGs^19,20^. **b** Selected pathways that are significantly enriched among hTSC EGs that overlap with core and primed hPSC EGs. **c** Selected pathways that are significantly enriched among hTSC-specific EGs. **d** The expression of 63 CTB-enriched hTSC-specific EGs in the CTB, EPI, and PrE of 10 d.p.f. human embryo scRNA-seq data^37^. **e** The expression of selected CTB-enriched hTSC-specific EGs in the CTB, EPI, and PrE of human embryo scRNA-seq data^37^. **f**–**g** Live cell counts relative to the mean of control sgRNA-transduced H9 hTSCs 4 (**f**) or 6 (**g**) days after seeding. Error bar represents standard error of three biological replicates. Two-tailed student’s t test was used for statistical analysis. “*” indicates a *p*-value<0.05, “**” indicates a *p*-value<0.01, and “***” indicates a *p*-value<0.001. The exact *p*-values from left to right are 0.000323207, 0.000310896, 0.004363306, 0.000530435, 0.002394918, and 0.005800072 (**f**). The exact *p*-values from left to right are 0.000193944, 0.00015954, 0.005236037, 0.00056065, 0.00285678, and 0.048627293 (**g**). **h** Phase contrast images of H9 hTSCs transduced with control or targeting sgRNAs at 4 days following seeding. The scale bars indicate 75 μm. The images are representative of three independent replicates in an experiment. **i** Experimental scheme to assess the requirement of TEAD1 during hTSC derivation and maintenance. **j** Phase contrast images of H9 WT and *TEAD1* KO hTSCs. The scale bars indicate 75 μm. The images are representative of two independent experiments. **k** Western blot confirming the absence TEAD1 protein in *TEAD1* KO hTSCs. The result is representative of two independent experiments. **l** Differential gene expression analysis between H9 WT and *TEAD1* KO hTSCs. WT contains two samples, and *TEAD1* KO contains two samples each from three independent clones. **m** Selected GO biological processes enriched among DEGs significantly upregulated or downregulated in *TEAD1* KO hTSCs relative to WT hTSCs. **n** Selected GO biological processes enriched among DARs significantly more open or closed in *TEAD1* KO hTSCs relative to WT hTSCs. **o** WT and *TEAD1* KO hTSC RNA-seq and ATAC-seq data shown in the vicinity of selected genes.

To better define regulators specific to hTSCs, we referenced our hTSC EGs to core EGs that largely contain common housekeeping genes^26^, as well as EGs identified through genetic screens in primed hPSCs (Fig. 2a)^19,20^. Since hTSCs and primed hPSCs correspond to post-implantation CTB and epiblast (EPI), respectively^10,38^, we reasoned that such a comparison may help identify essential regulators unique to these extraembryonic stem cells. While the hTSC EGs overlapped considerably with the core and especially primed hPSC EGs, we also identified 872 EGs specific to hTSCs (Fig. 2a and Supplementary Data 1), which were mainly localized to the nucleus and cytosol (Supplementary Fig. 4a). Pathways enriched in EGs shared between hTSCs and other cell types included housekeeping functions such as ribosome and spliceosome (Fig. 2b and Supplementary Data 2), while those unique to hTSC-specific EGs included HDAC and TNF signaling pathways (Fig. 2c and Supplementary Data 2)^8,39^. We then sought to further distinguish hTSC-specific regulators based on gene expression in the 10 and 12 d.p.f. human embryo, since genes with increased expression in CTBs relative to other embryonic and extraembryonic lineages more likely represent critical regulators of the trophoblast lineage in vivo (Fig. 2d, Supplementary Fig. 4b, and Supplementary Data 1)^10,37^. These genes included known trophoblast regulators such as *ARID3A*^40^, *CTNNB1*^8^, *GATA2*^31,41^, and *SKP2*^42^, as well as genes like *ARID5B*, *TCAF1*, and *TEAD1*, which have not previously been associated with trophoblast biology (Fig. 2e and Supplementary Fig. 4c). Screening data from intermediate timepoints showed that while core EGs became essential early in the screen, EGs more relevant to hTSC biology achieved essentiality at later time points (Supplementary Fig. 4d and Supplementary Data 1). We then validated the essentiality of *SKP2*, *TEAD1*, and *TCAF1* via proliferation assays in hTSCs derived from H9 naïve hPSCs^10^ to ensure reproducibility in a different genetic background than the one used for the screen. hTSCs transduced with targeting sgRNAs displayed significantly fewer cells compared to cells transduced with non-targeting sgRNAs (Fig. 2f–h and Supplementary Fig. 4e, f). For most of these targets, the fraction of sgRNA-transduced cells that contain frameshift mutations declined over time, which indicates that they were outcompeted by cells that are wild-type or contain in-frame mutations. These results demonstrate that our screen has identified EGs that are functionally relevant for the maintenance of hTSCs.

### Investigating the role of TEAD1 in hTSC specification and maintenance

We were particularly intrigued by the hTSC-specific essential regulator *TEAD1*. While its paralog *TEAD4* has been shown to be instrumental for mouse and human TE specification^32,34,35,43^, *TEAD1* was reportedly dispensable for mouse placentation^34^. However, *TEAD1* ranked more highly (#129) in our screen for hTSC EGs compared to *TEAD4* (#1407), and its downregulation has been associated with recurrent spontaneous abortion^44^, suggesting that it may have a human-specific role in trophoblast regulation. To explore this hypothesis, we utilized CRISPR-Cas9-mediated genome editing to generate stable *TEAD1* homozygous knockout (KO) cell lines in the H9 primed hPSC background (Fig. 2i). We then reset wildtype (WT) control and *TEAD1* KO primed hPSCs to the naïve state using our chemically defined conditions^45,46^ (Fig. 2i). The removal of *TEAD1* did not impair the ability to generate naïve hPSCs as evidenced by flow cytometry analysis for the naïve-specific cell surface markers CD75^47^ and SUSD2^48^ (Supplementary Fig. 5a). However, when we assayed the ability of these different genotypes to give rise to hTSCs (Fig. 2i), *TEAD1* KO cells displayed significantly impaired proliferation (Supplementary Fig. 5b–c). Analysis of hTSC specification dynamics by flow cytometry revealed that *TEAD1* KO cells adopt an hTSC identity with reduced kinetics compared to WT cells early in the derivation process (Supplementary Fig. 5d), although stable *TEAD1* KO hTSC cell lines can still be obtained (Fig. 2j, k and Supplementary Fig. 5d). These findings indicate that *TEAD1* promotes the efficient derivation of hTSCs from the naïve state.

We proceeded to investigate the consequences of the loss of *TEAD1* in hTSCs. *TEAD1* KO hTSCs had slightly increased G0/G1 phase and decreased S phase populations compared to WT hTSCs based on cell cycle analysis (Supplementary Fig. 5e), although levels of Annexin V-positive apoptotic cells were similar between the two genotypes (Supplementary Fig. 5f). We then performed RNA-seq on WT and *TEAD1* KO hTSCs to characterize their global gene expression changes. There were 162 genes significantly downregulated in *TEAD1* KO hTSCs (*p*-adjust < 0.05) (Fig. 2l), including many trophoblast regulators such as *FOS*^49,50^, *FN1*^51^, *ITGB8*^52^, *MET*^53^, and *LOXL2*^54^. Genes downregulated in *TEAD1* KO hTSCs were enriched for GO terms such as integrin-mediated signaling pathway, regulation of TGF-β production, and regulation of cell motility (Fig. 2l, m). Interestingly, a large number of genes downregulated in *TEAD1* KO hTSCs are known to promote EVT differentiation; in fact, the EVT marker *HLA-G* was the most downregulated gene (Fig. 2l). On the other hand, there were 251 genes significantly upregulated in *TEAD1* KO hTSCs (*p*-adjust < 0.05), including the trophoblast regulators *AMOTL2*^55^, *TET2*^56^, and *CITED2*^57^ (Fig. 2l). Many STB marker genes such as *CGB2*, *CGB7*, and *PSG11* were also upregulated in *TEAD1* KO hTSCs (Fig. 2l). GO analysis revealed that genes upregulated in KO were enriched for terms such as response to hormone, cell adhesion, and apoptosis (Fig. 2m). We also used the Assay for Transposase Accessible Chromatin with high-throughput sequencing (ATAC-seq) to compare the chromatin accessibility landscape in *TEAD1* WT and KO hTSCs. There were 3988 and 4116 peaks that significantly lost or gained chromatin accessibility upon the loss of TEAD1, respectively (*p*-adjust < 0.05). Peaks with decreased ATAC-seq signal were enriched for GO terms such as placenta development, activation of JUN kinase activity, and embryo implantation, while those with increased signal were enriched for cytokine regulation, transmembrane transport, and peptide hormone processing (Fig. 2n). A number of key trophoblast regulators displayed significant chromatin accessibility changes consistent with reduced or increased expression in *TEAD1* KO hTSCs, including *ZFP57*^58^, *FN1*^51^ (downregulated), and *CBR1*^59^ (upregulated) (Fig. 2o).

**Fig. 3:**
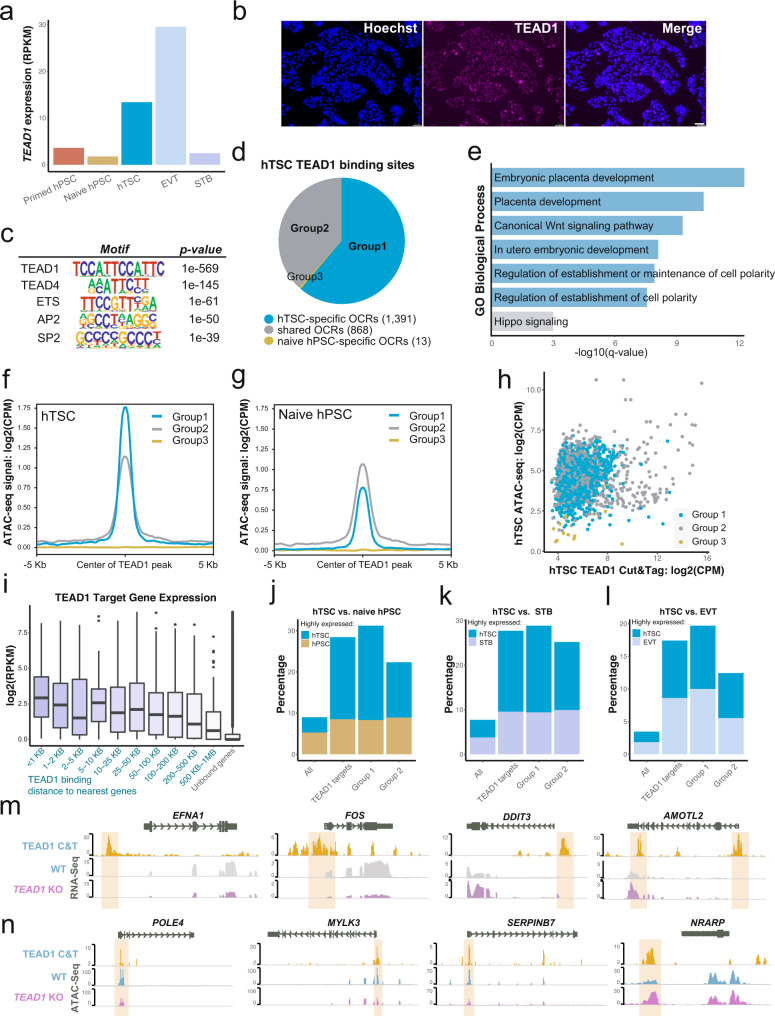
Investigation of TEAD1 targets in hTSCs. **a** TEAD1 gene expression in published AN and H9 pluripotent and trophoblast cell types^10^. **b** Immunofluorescence staining for TEAD1 in H9 hTSCs. The scale bars indicate 75 μm. The experiment was performed once. **c** Selected TF binding motifs significantly enriched among TEAD1 CUT&Tag peaks and their *p*-values according to HOMER Motif Analysis. **d** TEAD1 CUT&Tag peaks overlapping with naïve hPSC or hTSC ATAC-seq peaks were categorized into three groups: those overlapping with hTSC-specific open chromatin regions (OCRs) (group 1), shared OCRs (group 2), or naïve hPSC-specific OCRs (group 3). **e** Top GO biological processes significantly enriched among group 1 (blue) and group 2 (gray) TEAD1 CUT&Tag peaks and their *p*-values. **f** The AN and H9 hTSC ATAC-seq signal over group 1-3 TEAD1 CUT&Tag peaks^10^. **g** The AN and H9 naïve hPSC ATAC-seq signal over groups 1–3 TEAD1 CUT&Tag peaks^10^. **h** CUT&Tag and hTSC ATAC-seq signal of group 1-3 TEAD1 CUT&Tag peaks. **i** Expression of genes in H9 hTSCs^10^, binned by the distance of TEAD1 CUT&Tag peaks to TSS. Two independent samples were used for analysis. Boxplot presents the 25th, median, and 75th quartiles, the whiskers extend 1.5 of interquartile ranges, and the dots are outside values >1.5 times and <3 times the interquartile range beyond either end of the box. **j**–**l** Percentage of all genes, all TEAD1 target genes, group 1 TEAD1 target genes, and group 2 (excluding those already in group 1) TEAD1 target genes that are significantly up- or downregulated in AN and H9 hTSCs vs. naïve hPSCs (**j**), hTSCs vs. STBs (**k**), hTSCs vs EVTs (**l**)^10^. **m** TEAD1 CUT&Tag and WT and *TEAD1* KO hTSC RNA-seq data shown in the vicinity of selected genes. **n** TEAD1 CUT&Tag and WT and *TEAD1* KO hTSC ATAC-seq data shown in the vicinity of selected genes.

*TEAD1* transcripts were upregulated during the transition from naïve hPSCs into hTSCs (Fig. 3a) and immunofluorescence analysis indicated that TEAD1 acquires a nuclear localization in hTSCs, which is consistent with its well-established role as a transcriptional co-factor for the Hippo effectors YAP/TAZ^60^ (Fig. 3b). To further investigate how this transcription factor regulates the hTSC state, we assayed its genome-wide DNA binding sites in H9 hTSCs via Cleavage Under Targets and Tagmentation (CUT&Tag)^61^. This analysis identified 3566 reproducible peaks in hTSCs, which were enriched for motifs such as TEAD1/4, ETS, and AP2, and GO terms related to placental development (Fig. 3c and Supplementary Fig. 6a–c). These peaks were associated with 2,568 genes, including a number of hTSC EGs (Supplementary Fig. 6d). We then intersected TEAD1 targets with open chromatin regions (OCRs) identified by ATAC-seq in naïve hPSCs and hTSCs^10^. Of the 2,272 TEAD1 peaks that overlapped with OCRs in either cell state, most were located at accessible chromatin regions that were specific to hTSCs (group 1) or shared between naïve hPSCs and hTSCs (group 2) (Fig. 3d). Top GO terms enriched at group 1 peaks included placental development and the WNT signaling pathway, whereas those enriched at group 2 peaks included Hippo signaling (Fig. 3e). While group 1 TEAD1 targets exhibited a high ATAC-seq signal in hTSCs (Fig. 3f), group 2 peaks showed a similar ATAC-seq signal in both cell types (Fig. 3f–h). The precocious opening in naïve hPSCs of such a large set of chromatin regions bound by TEAD1 in hTSCs may partly explain why naïve hPSCs display an enhanced potential for trophoblast differentiation^10,12,13,62^.

The binding of TEAD1 was associated with enhanced gene expression in hTSCs, particularly when the transcriptional start site was located within 1 kb from a TEAD1 peak (Fig. 3i). Expression of TEAD1 target genes, especially those associated with group 1 peaks, was significantly elevated in hTSCs relative to naïve hPSCs or STBs, one of two specialized trophoblast cell types that can be derived from hTSCs in vitro^8,10^ (Fig. 3j, k and Supplementary Fig. 6e). Similar trends were observed in vivo (Supplementary Fig. 6f, g), suggesting that *TEAD1* helps to specify and regulate hTSCs by activating CTB-specific transcriptional programs. However, TEAD1 target genes were not biased toward up- or downregulation in EVTs versus hTSCs (Fig. 3l and Supplementary Fig. 6h), which may point to an additional role for TEAD1 during differentiation into the EVT lineage. Finally, to uncover the direct regulation of gene expression and chromatin accessibility by TEAD1, we referenced group 1 and 2 TEAD1 CUT&Tag targets to differentially expressed genes (DEGs) and differentially accessible regions (DARs) between WT and *TEAD1* KO hTSCs. Integration with RNA-seq data indicated that 26 and 34 TEAD1 target genes were down- and upregulated in *TEAD1* KO hTSCs, respectively (Supplementary Fig. 6i). For example, the direct TEAD1 targets *FOS*^49,50,63^ and *EFNA1*^64,65^ were downregulated in *TEAD1* KO hTSCs, while *AMOTL2*^55^ and *DDIT3*^66^ were upregulated in *TEAD1* KO hTSCs (Fig. 3m). Meanwhile, integration with ATAC-seq data indicated that 141 and 14 TEAD1 targets displayed reduced or increased chromatin accessibility in *TEAD1* KO hTSCs, respectively (Supplementary Fig. 6j). For example, chromatin accessibility was reduced at TEAD1 binding sites within the *POLE4*, *MYLK3*, and *SERPINB7* loci, and increased at a TEAD1 binding site within the *NRARP* locus^67^ (Fig. 3n). Collectively, these data indicate that TEAD1 maintains hTSC identity by regulating proper cell cycling, modulating key trophoblast regulators, and suppressing the STB program via direct or indirect mechanisms.

### Investigating the role of TEAD1 in EVT and STB differentiation

**Fig. 4:**
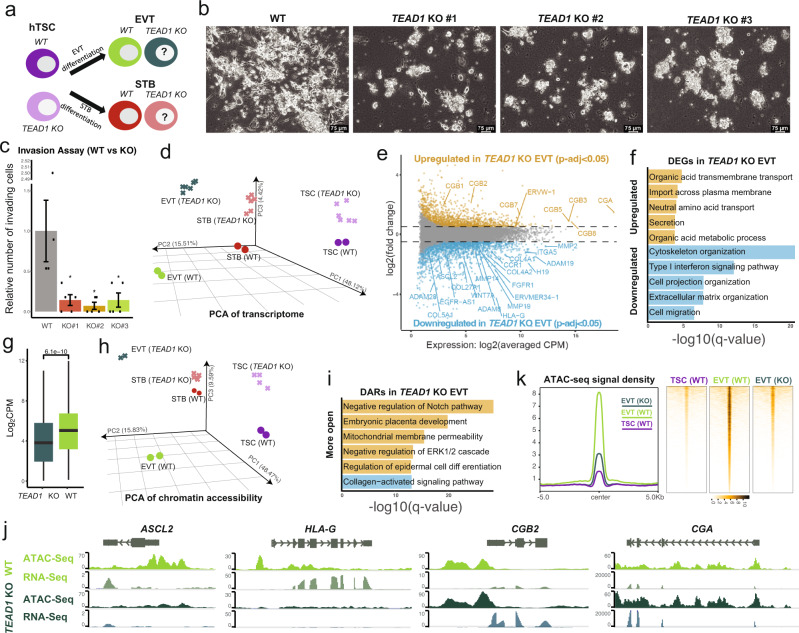
Investigating the role of TEAD1 in EVT differentiation. **a** Experimental scheme to assess the requirement of TEAD1 during EVT and STB differentiation. **b** Phase contrast images of H9 WT EVTs and *TEAD1* KO hTSCs that have undergone EVT differentiation. The scale bars indicate 75 μm. The images are representative of four independent experiments. **c** The relative number of invading cells following Matrigel invasion assay of H9 WT EVTs and *TEAD1* KO hTSCs that have undergone EVT differentiation. Error bars indicate ±1 standard error of five technical replicates. The center of the error bar indicates the mean. One-tailed student’s t test was used for statistical analysis.‘*‘ indicates a *p*-value <0.05. The exact *p*-values were 0.028856521 (#1), 0.020949253 (#2), and 0.029918938 (#3). **d** Principal component analysis (PCA) of WT and *TEAD1* KO hTSCs, EVTs, and STBs based on RNA-seq data. **e** Scatter plot showing the differential gene expression analysis between H9 WT and *TEAD1* KO EVTs. WT contains two RNA-seq samples, and *TEAD1* KO contains two RNA-seq samples each from three independent clones. **f** Selected GO biological processes that are enriched among DEGs significantly upregulated or downregulated in *TEAD1* KO “EVTs” relative to WT EVTs. **g** The expression of EVT-specific genes (*n* = 462)^37^ in H9 WT and *TEAD1* KO EVTs. WT contains two RNA-seq samples, and *TEAD1* KO contains two RNA-seq samples each from three independent clones. Two-tailed Wilcoxon Rank Sum Test was used for statistical analysis. Boxplot presents the 25th, median, and 75th quartiles, the whiskers extend 1.5 of interquartile ranges. **h** Principal component analysis (PCA) of WT and *TEAD1* KO hTSCs, EVTs, and STBs based on ATAC-seq data. **i** Selected GO biological processes that are enriched among DARs significantly more open or closed (blue) in *TEAD1* KO “EVTs” relative to WT EVTs. **j** WT and *TEAD1* KO EVT RNA-seq and ATAC-seq data shown in the vicinity of selected genes. **k** Levels of WT hTSC and WT and *TEAD1* KO EVT ATAC-seq signal over regions that are specifically open in WT EVTs. Each condition contains two ATAC-seq samples.

Given the differential RNA-seq and ATAC-seq signals of EVT- and STB-associated genes in WT and *TEAD1* KO hTSCs (Fig. 2l–o) and the expression levels of TEAD1 and its target genes in different trophoblast cell types (Fig. 3a, j–l, and Supplementary Fig. 6f–h), we hypothesized that TEAD1 may promote the differentiation of hTSCs into the EVT lineage, but present a barrier to STB differentiation (Fig. 4a). To test this hypothesis, we first performed lineage-directed EVT differentiation on WT and *TEAD1* KO hTSCs by treating the cells with Neuregulin (NRG1) and the TGF-β inhibitor A83-01 in the presence of Matrigel^8,10^. Unlike WT cells, *TEAD1* KO cells subjected to this EVT differentiation protocol failed to acquire the typical spindle-like EVT morphology and were far less invasive in a Matrigel-coated transwell assay (Fig. 4b, c). We profiled the transcriptomes of WT and *TEAD1* KO samples by RNA-seq and all samples were clearly separated by cell type and genotype in the PCA (Fig. 4d). *TEAD1* KO “EVTs” displayed significant downregulation of 1,715 genes and upregulation 1,328 genes compared to WT EVTs (*p*-adjust < 0.05) (Fig. 4e). Downregulated DEGs included key EVT markers such as *ASCL2*, *ITGA5*, and *HLA-G* (Fig. 4e), and were enriched for GO terms like cytoskeleton organization, cell projection organization, and cell migration (Fig. 4f). The expression of EVT-specific genes reported by the human embryo scRNA-seq data^37^ was also significantly lower in *TEAD1* KO compared to WT EVTs (Fig. 4g). On the contrary, DEGs that were upregulated in *TEAD1* KO EVTs consisted of a large number of STB regulators such as *CGA*, *CGB*, and *ERVW-1*, and were enriched for GO terms such as organic acid transmembrane transport, neutral amino acid transport, and secretion, which are typically associated with the function of STBs in mediating maternal-fetal exchange (Fig. 4e, f). We also profiled the chromatin accessibility landscapes of WT and *TEAD1* KO EVTs via ATAC-seq. All samples were again clearly separated by cell type and genotype in the PCA (Fig. 4h). There were 24,675 and 10,026 loci with decreased or increased ATAC-seq signal in *TEAD1* KO EVTs, respectively. Based on GO term enrichment analysis, the collagen-activated signaling pathway was enriched among genes with reduced chromatin accessibility in *TEAD1* KO cells, while terms including negative regulation of Notch signaling pathway^68^, embryonic placenta development, and negative regulation of ERK1 and ERK2 cascade^69^ were enriched among chromatin that gained accessibility (Fig. 4i). Similar to our observations in hTSCs, changes in ATAC-seq intensity in *TEAD1* KO EVTs were linked to changes in gene expression for a number of trophoblast regulators, including *ASCL2*^70^ and *HLA-G* (downregulated), and *CGA* and *CGB2* (upregulated) (Fig. 4j). Finally, when we probed the ATAC-seq signal at EVT-specific open chromatin, we discovered that *TEAD1* KO “EVTs” failed to properly open those regions (Fig. 4k). These data lead us to conclude that *TEAD1* is critical for EVT differentiation, while potentially hindering STB differentiation, and that loss of *TEAD1* dysregulates EVT-specific gene expression and accessible chromatin.

To determine whether *TEAD1* KO hTSCs may have an enhanced potential for STB differentiation, we performed lineage-directed STB differentiation on WT and *TEAD1* KO hTSCs by applying the cyclic AMP agonist Forskolin together with epidermal growth factor (EGF). Interestingly, *TEAD1* KO STBs were more abundant than WT STBs towards the end of the differentiation time course (Supplementary Fig. 7a). RNA-seq analysis revealed 1039 and 880 DEGs that were significantly downregulated or upregulated in *TEAD1* KO STBs, respectively (*p*-adjust < 0.05) (Supplementary Fig. 7b). Genes with significantly higher expression in *TEAD1* KO cells included the STB markers *CGB1*, *CGB2*, and *SLC38A3*^37^ (Supplementary Fig. 7b), and were enriched for GO terms such as ribonucleoside bisphosphate biosynthetic process, response to organic cyclic compound, and *in utero* embryonic development (Supplementary Fig. 7c). Conversely, genes significantly downregulated in *TEAD1* KO STBs were enriched for GO terms related to cytokine response and regulation of cell death (Supplementary Fig. 7c). We also performed ATAC-seq on these cells, which revealed 19,629 and 29,025 DARs that became significantly more closed or open in *TEAD1* KO STBs, respectively. Open DARs were enriched for glucose transport, and closed DARs were enriched for regulation of gonadotropin secretion (Supplementary Fig. 7d). We again found that DARs were correlated to gene expression changes for a number of key trophoblast regulators (Supplementary Fig. 7e). Genes that gained ATAC-seq and RNA-seq signals in *TEAD1* KO STBs included STB-specific genes such as *FIBCD1*^37^, while those that lost ATAC-seq and RNA-seq signals included hTSC-specific genes like *ITGA2*^71^, *KRT17*^72^, and *IFITM2*^73^ (Supplementary Fig. 7e). These data suggest that TEAD1 indeed acts as a barrier for STB differentiation and that *TEAD1* KO STBs are potentially more mature than WT STBs. Taken together, we postulate that TEAD1 performs a variety of functions during the induction, maintenance, and differentiation of hTSCs by modulating the expression and chromatin accessibility of target trophoblast regulators.

### Identification and characterization of hTSC GRGs

**Fig. 5:**
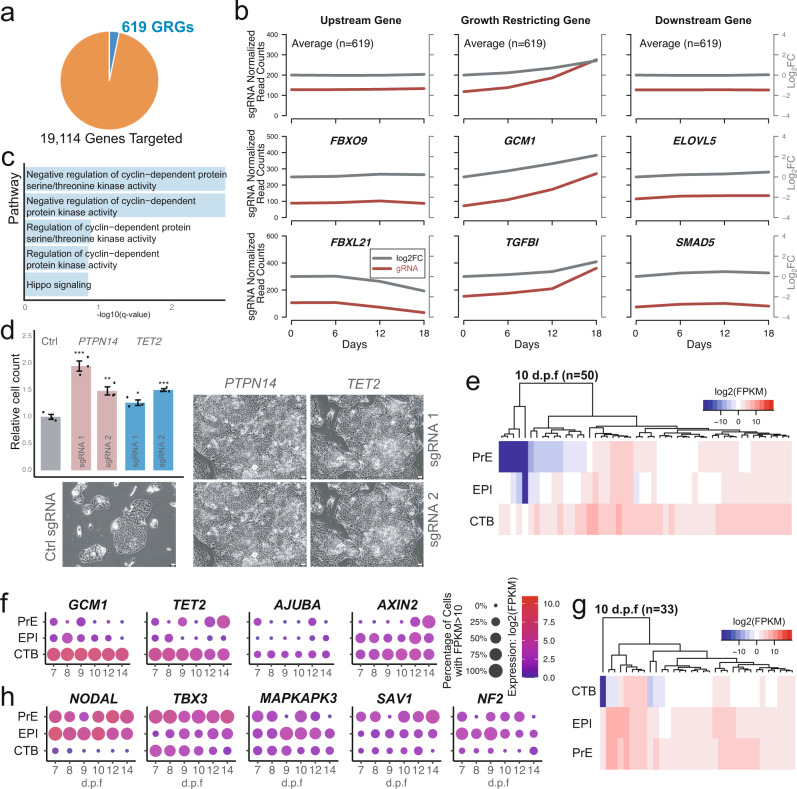
Identification and characterization of hTSC GRGs. **a** Growth-restricting genes (GRGs) identified among all targeted genes. **b** The mean of sgRNA normalized read counts and Log2 fold changes of all or selected hTSC GRGs and their neighboring up- and downstream genes over time. The results are representative of two independently transduced screening experiments. **c** Top 5 most enriched GO Biological Processes terms among all hTSC GRGs. **d** Top left, live cell counts relative to the mean of control sgRNA transduced H9 hTSCs 6 days after seeding. Error bar represents the standard error of three biological replicates. Two-tailed student’s t test was used for statistical analysis. “*” indicates a *p*-value < 0.05, “**” indicates a *p*-value < 0.01, and “***” indicates a *p*-value < 0.001. The exact p-values from left to right are 0.000870208, 0.005413584, 0.018757677, and 0.000646371. Rest of the panel, phase contrast images of H9 naïve hTSCs transduced with control or targeting sgRNAs at 6 days following seeding. The scale bars indicate 75 μm. The images are representative of three independent replicates in an experiment. **e** Heatmap showing the expression of 50 CTB-enriched GRGs in the CTB, EPI, and PrE of published 10 d.p.f. human embryo scRNA-seq data^37^. **f** Dot plot showing the expression of selected CTB-enriched GRGs in the CTB, EPI, and PrE of published human embryo scRNA-seq data^37^. **g** Heatmap showing the expression of 33 CTB-depleted GRGs in the CTB, EPI, and PrE of published 10 d.p.f. human embryo scRNA-seq data^37^. **h** Dot plot showing the expression of selected CTB-depleted GRGs in the CTB, EPI, and PrE of published human embryo scRNA-seq data^37^.

Since the failure to restrict cellular growth often leads to diseases such as cancer, identification of GRGs is equally important for understanding regulatory mechanisms in hTSCs, especially given their potential relevance to choriocarnicoma^74^. Our screen identified 619 GRGs in hTSCs (Fig. 5a, Supplementary Fig. 8a, and Supplementary Data 3), which mainly showed nuclear and cytosolic localizations (Supplementary Fig. 8b). The mean normalized read counts and log2 fold changes of all sgRNAs targeting GRGs steadily increased throughout the screen, while those targeting neighboring genes remained unchanged (Fig. 5b). Among the GRGs in hTSCs were *GCM1*, which encodes a transcription factor involved in STB differentiation^75–77^, and *TGFBI*, which is induced by TGFβ signaling and encodes a secreted extracellular matrix protein^78^. Consistent with the growth-restricting function of *TGFBI* in hTSCs, pharmacological inhibition of TGFβ signaling is integral to hTSC derivation and maintenance^8^ (Fig. 5b). Other GRGs included the genes encoding the phosphatase PTPN14, the 5-methylcytosine hydroxylase TET2, and the orphan receptor NR6A1 (Supplementary Fig. 8c). hTSC GRGs were the most enriched in pathways including negative regulation of cyclin-dependent kinases and negative regulation of Hippo signaling (that prevent YAP/TAZ nuclear translocation), such as *SAV1*, *LATS1*, *AMOTL2*, and *AJUBA* (Fig. 5c and Supplementary Data 2). These findings highlight the crucial role of Hippo signaling in regulating hTSCs. We validated the growth-restricting function of two of the GRGs, *PTPN14* and *TET2*, via proliferation assays in H9 hTSCs following sgRNA transduction (Fig. 5d and Supplementary Fig. 8d, e). As expected, the fraction of sgRNA-transduced cells bearing frameshift mutations in these target genes increased over time. It has been reported that PTPN14 directly inhibits *YAP* in cancer cells in a density-dependent manner^79–81^. Interestingly, TET2 reportedly cooperates with TET1 to maintain mTSC self-renewal^56^, suggesting a potentially disparate role in mouse versus human trophoblast.

We proceeded to uncover GRGs that have unique expression patterns in CTBs relative to other lineages in human embryos cultured through implantation stages^37^. First, we identified GRGs uniquely upregulated in CTBs at 10 and 12 d.p.f., which represents the stage of in vivo trophoblast development most closely aligned with hTSCs (Fig. 5e, Supplementary Fig. 8f, and Supplementary Data 3). These included the genes encoding GCM1^75,76^, the mTSC cell cycle modulators TET2 and E2F8^56,82^, the negative Hippo regulator AJUBA, and the negative WNT regulator AXIN2 (Fig. 5f and Supplementary Fig. 8g). We also identified GRGs uniquely depleted in CTBs at 10 or 12 d.p.f. (Fig. 5g, Supplementary Fig. 8h, and Supplementary Data 3), such as *NODAL*, *TBX3*, and *MAPKAPK3* (Fig. 5h). The protein products of these genes include important components of the pluripotency network^83–85^, although *TBX3* has also been implicated in human STB development^86^. CTB-depleted GRGs also included genes encoding the negative Hippo regulators SAV1 and NF2 (Fig. 5h), which ensure ICM specification and prevent ectopic TE formation^87^. We found that hTSC GRGs mostly belonged to three different categories. The first were primarily housekeeping genes, exemplified by the enrichment of cell-cycle related pathways (Fig. 5c). The second category of GRGs included genes such as *AJUBA* and *AXIN2* that antagonize pathways essential for hTSCs. The third included GRGs that promote other cellular identities, for instance, *GCM1* (STB) and *NODAL* (EPI). Hence, two key functions of hTSC GRGs are to prevent the over-proliferation of hTSCs and the ectopic activation of hTSC-specific transcriptional programs in other cell types. Overall, our analysis revealed that hTSC GRGs are a diverse group of genes that together ensure the balanced development of the early human embryo.

### Analysis of hTSC EGs and GRGs implicated in mouse placentation

**Fig. 6:**
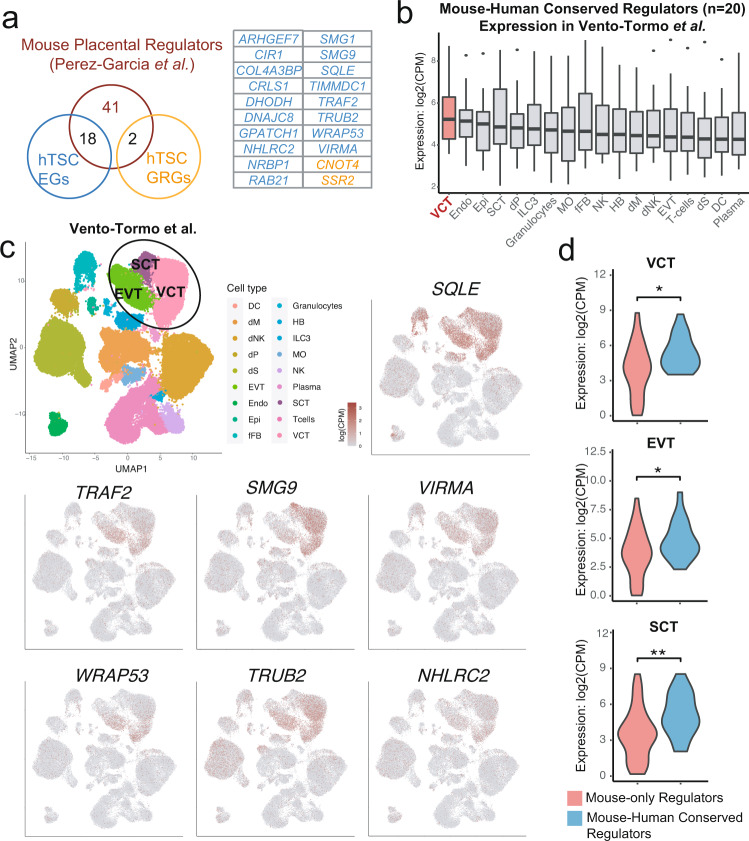
Comparison of hTSC EGs and GRGs with genes required for mouse placentation. **a** Overlap of hTSC EGs and GRGs with a published list of embryonic lethal genes known to cause placental defects in mouse^90^. **b** Expression of genes that lead to mouse placentation defects and overlap with hTSC EG/GRG lists (*n* = 20) in different cell types found in published human maternal-fetal interface scRNA-seq dataset^91^. Cell types were ranked based on mean gene expression. Boxplot presents the 25th, median, and 75th quartiles, the whiskers extend 1.5 of interquartile ranges, and the dots are outside values >1.5 times and <3 times the interquartile range beyond either end of the box. **c** Expression visualization of selected mouse-human conserved regulators (Fig. 6a) in the human maternal-fetal interface scRNA-seq dataset^91^. **d** Expression of genes required for mouse placentation that do not (*n* = 41) and do (*n* = 20) overlap with hTSC EGs and GRGs in the VCTs (CTBs), EVTs, and SCTs (STBs) of published human maternal-fetal interface scRNA-seq dataset^91^. Two-tailed Wilcoxon Rank Sum Test was used for statistical analysis. ‘*’ indicates a *p*-value < 0.05 and ‘**’ indicates a *p*-value < 0.01. The exact *p*-values are 0.011 (VCT), 0.047 (EVT), and 0.0033 (SCT). VCT: 9479 cells; EVT: 3626 cells; SCT: 1261 cells.

It is well-established that given the evolutionary diversity of the mammalian placenta, animal models inadequately recapitulate human placental development^88,89^. Therefore, we anticipated that few hTSC EGs and GRGs would be conserved regulators of mouse placental development. To address this question, we compared hTSC EGs and GRGs with regulators of mouse placentation identified by a large-scale analysis of mouse gene knockouts that cause intrauterine lethality^90^. Of the 61 genes that led to mouse placental defects^90^, 18 were identified as EGs and 2 as GRGs in hTSCs in this study (Fig. 6a). We found that these 20 conserved placental regulators were expressed more highly in villous cytotrophoblast (VCT or CTB) than in other placental, decidual, or immune cell types in scRNA-seq data sampled from the human maternal-fetal interface^91^ (Fig. 6b, c and Supplementary Fig. 9a). These 20 conserved regulators were also expressed more highly than mouse-specific placental regulators (*n* = 41) in various human trophoblast cell types (Fig. 6d)^91^, while there was no significant expression difference in various mouse trophoblast cell types (Supplementary Fig. 9b)^92^. These data suggest that our screen has identified conserved placental regulators that are robustly expressed during human placental development. The conserved regulators include *TRAF2* (Fig. 6c), which encodes a member of the TNF signaling pathway that promotes the activation of NF-κB and MAPK pathways downstream^93^, as well as multiple mitochondrial genes such as *TRUB2*^94^, *TIMMDC1*^95^, *CRLS1*^96,97^, and *DHODH*^98^ (Fig. 6c and Supplementary Fig. 9a), highlighting the importance of mitochondrial function and oxidative phosphorylation in trophoblast development^99^. Several other conserved regulators with strong enrichment in trophoblast cells include *SQLE*, *SMG9*, *VIRMA*, *WRAP53*, *NHLRC2*, and *GPATCH1* (Fig. 6c and Supplementary Fig. 9a). We also confirmed our findings by comparing our hTSC regulators to an independent list of mouse placenta regulators curated by the Jackson Laboratory (Supplementary Fig. 9c–e)^100^. Lastly, we observed a sizable overlap between hTSC EGs/GRGs and genes associated with pregnancy-related diseases (Supplementary Fig. 9f, g)^101,102^, which suggests that hTSCs could provide a powerful cellular model system to study trophoblast-related pathologies.

In conclusion, we present the findings from a genome-wide CRISPR-Cas9 knockout screen in hTSCs. By identifying and characterizing hTSC EGs and GRGs, we systematically uncovered potential regulators of the hTSC state. Genetic deletion studies indicated that the removal of one such regulator, *TEAD1*, impaired the generation and maintenance of hTSCs and prevented the faithful differentiation of hTSCs into the invasive EVT lineage. Furthermore, integrated analysis of ATAC-seq, CUT&Tag, and RNA-seq data demonstrated that TEAD1 regulates the gene expression and chromatin accessibility of many trophoblast regulators in hTSCs, suggesting that this transcription factor plays an important role during progressive stages of human trophoblast development. The hTSC EGs and GRGs also overlapped with pregnancy disease-associated genes and regulators of placentation during mouse development in vivo^90,100^, which provides a framework for elucidating conserved and species-specific regulators of trophoblast development. Overall, our study provides a valuable resource for future research into the genetic circuitry controlling placental development and disease.

## Methods

### Ethics statement

All human embryonic stem cell experiments performed in this study have been approved by Embryonic Stem Cell Research Oversight Committee at Washington University School of Medicine.

### Culture of hTSCs

hTSCs were cultured as previously described^8,10^. Prior to seeding, 6-well plates were coated with 5 μg/mL Collagen IV (Corning, 354233) at 37 °C overnight. Cells were cultured in 2 mL hTSC medium [DMEM/F12 (Gibco, 11320) supplemented with 0.1 mM 2-mercaptoethanol (Millipore Sigma, 8.05740), 0.2% FBS (Millipore Sigma, ES-009-B), 0.5% Penicillin-Streptomycin (Gibco, 15140), 0.3% BSA (Gibco, 15260), 1% ITS-X (Gibco, 51500), 1.5 μg/ml L-ascorbic acid (Wako, 013-12061), 50 ng/ml EGF (Peprotech, AF-100-15), 2 μM CHIR99021 (Stemgent, 04-0004), 0.5 μM A83-01 (BioVision, 1725), 1 μM SB431542 (BioVision, 1674), 0.8 mM VPA (Sigma, P4543), and 5 μM Y-27632 (Stemgent, 04-0012)] and in 5% CO_2_ and 20% O_2_. Tissue culture media were filtered using a 0.22 μm filter. Media were changed every 2 days, and cells were passaged using TrypLE Express (Gibco, 12604). Unless otherwise specified, hTSCs within 30 passages were used for experiments. The cell culture is regularly tested and confirmed negative for mycoplasma contamination.

### Culture of naïve and primed hPSCs

Primed hPSCs were cultured in mTeSR1 (STEMCELL Technologies, 85850) on Matrigel (Corning, 354277) coated wells and passaged using ReLeSR (STEMCELL Technologies, 05872) every 4–6 days. Primed hPSCs were cultured in 5% CO_2_ and 20% O_2_. Naïve hPSCs were cultured on mitomycin C-inactivated mouse embryonic fibroblast (MEF) feeder cells and were passaged by a brief PBS wash followed by single-cell dissociation using 5 min treatment with TrypLE Express. Naïve hPSCs used for ATAC-seq experiments were cultured in the 5i/L/A media as previously described^46,103^. 500 mL of 5i/L/A was generated by supplementing N2B27 [240 mL DMEM/F12, 240 mL Neurobasal (Gibco, 21103), 5 mL N2 100X supplement (Gibco, 17502), 10 mL B27 50X supplement (Gibco, 17504), 1X GlutaMAX (Gibco, 35050), 1X MEM NEAA (Gibco, 11140), 0.1 mM β-mercaptoethanol, 1% penicillin-streptomycin, and 50 µg/ml BSA Fraction V] with the following small molecules and cytokines: 1 μM PD0325901 (Stemgent, 04-0006), 1 μM IM-12 (Enzo, BML-WN102), 0.5 μM SB590885 (Tocris, 2650), 1 μM WH4-023 (A Chemtek, H620061), 10 μM Y-27632, 10 μg recombinant human LIF (Peprotech, 300-05), and 10 ng/mL Activin A (Peprotech, 120-14). To increase the growth rate, control and *TEAD1* KO naïve hPSCs were expanded in PXGLY medium [N2B27 supplemented with 1 μM PD0325901, 2 μM XAV939 (Selleckchem, S1180), 2 μM Gö6983 (Tocris, 2285), 10 μM Y-27632, and 10 ng/mL human LIF]^104^ prior to hTSC derivation. Naïve hPSCs were cultured in 5% O_2_, 5% CO_2_. For primed-to-naïve hPSC conversion, 2.5 × 10^5^ single primed cells were seeded on a six-well plate with MEF feeder layer in 2 mL mTeSR1 supplemented with 10 μM Y-27632. Two days later, medium was switched to 5i/L/A. After 6 to 10 days from seeding, the cells were expanded polyclonally using TrypLE Express on a MEF feeder layer. Tissue culture media were filtered using a 0.22 μm filter. Media were changed every 1–2 days. Naïve hPSCs before passage 10 were used for experiments. The cell culture is regularly tested and confirmed negative for mycoplasma contamination.

### Derivation of hTSCs from naïve hPSCs

hTSCs were derived from naïve hPSCs according to our recently described methodology^10,105^. Briefly, naïve hPSCs were single-cell dissociated by TrypLE Express, and 0.25 × 10^6^ cells were seeded on a six-well plate with MEF feeder layer and cultured in 2 mL naïve media for 1 day in 5% CO_2_ and 5% O_2_. On the following day, the cells were cultured in hTSC medium in 5% CO_2_ and 20% O_2_, and media were replaced every 2 days. Upon reaching 80–100% confluency, the cells were passaged at a ratio of 1:2 to 1:4 to six-well plates pre-coated with 5 μg/mL Collagen IV, and cultured from this point on as regular hTSCs. Cell numbers were quantified at every passage to obtain the cumulative cell count for the TEAD1 knockout experiment. Trypan Blue (Invitrogen, T10282) was used to exclude dead cells.

### EVT and STB differentiation from hTSCs

Differentiation of hTSCs into terminal cell types was performed as previously described^8^, with minor modifications. Prior to differentiation, hTSCs were grown to about 80% confluency, and then single-cell dissociated using TrypLE Express. For EVT differentiation, six-well plates were coated with 1 μg/mL Collagen IV overnight. On day 0, 0.75 × 10^5^ hTSCs were seeded per well in 2 mL EVT basal medium [DMEM/F12 supplemented with 0.1 mM β-mercaptoethanol, 0.5% penicillin-streptomycin, 0.3% BSA, 1% ITS-X, 7.5 μM A83-01, 2.5 μM Y27632] supplemented with 4% KSR (Gibco, 10828) and 100 ng/mL NRG1 (Cell signaling, 5218SC). Matrigel was added to a 2% final concentration shortly after resuspending hTSCs in the medium. At day 3, the media were replaced with 2 mL EVT basal medium supplemented with 4% KSR, and Matrigel was added to a 0.5% final concentration. At day 6, the media were replaced with 2 mL EVT basal medium, and Matrigel was added to a 0.5% final concentration. At day 8, the cells were ready for downstream analysis.

For STB differentiation, 2.5 × 10^5^ hTSCs were seeded per well in 3 mL 3D STB medium [DMEM/F12 supplemented with 0.1 mM β-mercaptoethanol, 0.5% penicillin-streptomycin, 0.3% BSA, 1% ITS-X, 2.5 μM Y-27632, 50 ng/ml EGF, 2 μM Forskolin (Sigma-Aldrich, F3917), and 4% KSR] in an ultra-low attachment six-well plate. At day 3, another 3 mL of 3D STB medium was added per well. At day 6, the cells were transferred to a 15 mL tube and gravity sedimented for 15 min, and the cells at the bottom of the tube were collected and used for downstream analysis.

### TEAD1 CRISPR targeting

Guide RNAs (gRNAs) aimed at introducing out-of-frame indels to trigger nonsense-mediated decay of *TEAD1* were designed and validated in K562 cells by the Genome Engineering and iPSC center (GEIC) at Washington University. In short, synthetic gRNAs were complexed with recombinant Cas9 protein and nucleofected into K562 cells. Transfected cells were harvested and lysed 48–72 h post-nucleofection. Each target region was PCR amplified, indexed, and analyzed on a MiSeq for insertion-deletion (indel) rate, indicative of cleavage activity. The most efficient gRNA sequence (TAGCCAGATACATCAAACTCNGG) was selected for nucleofection into primed hPSCs. 1.5 × 10^6^ primed H9 hPSCs were transfected with 0.5 µg pmaxGFP control vector, 300 pmol gRNA, and 192 pmol Cas9 protein. gRNA complexes targeting *TEAD1* were transfected into primed hPSCs by nucleofection using the Amaxa P3 Primary Cell 4D-Nucleofector X Kit and 4D-Nucleofector device with program CA-137 (Lonza). 3 days after nucleofection, the cells were single cell dissociated and sorted for GFP-expressing (~5%) cells using a MoFlo flow cytometry system. Clonal lines were analyzed by NGS for presence of indels around the cut site. Samples that exhibited a mixed indel ratio were discarded. Primed *TEAD1*^*+/+*^ (WT) and *TEAD1*^*−/−*^ hPSC clones were selected for subsequent analysis.

### Cloning

Individual sgRNA lentiviral expression vector cloning was performed as previously described^17^. Briefly, LentiCRISPR v2 (Addgene, #52961) plasmid was digested with Esp3I restriction enzyme (Thermo Fisher, FD0454), dephosphorylated with FastAP (Thermo Fisher, EF0651), and gel purified. sgRNA oligos (sequence taken from the Brunello library and listed below) were phosphorylated with T4 PNK (NEB, M0201S) and annealed. The digested plasmid and annealed oligos were then ligated using Quick Ligase (NEB, M2200S) and transformed into One Shot StbI3 Chemically Competent cells (Thermo Scientific, C737303). Single colonies were then picked, and the identities of the clones were verified using Sanger sequencing.

**Table:** 

TEAD1 CRISPR KO gRNA 1 F	CACCGACTGCCATTCATAACAAGCT
TEAD1 CRISPR KO gRNA 1 R	AAACAGCTTGTTATGAATGGCAGTC
TEAD1 CRISPR KO gRNA 2 F	CACCGAGATACATCAAACTCAGGAC
TEAD1 CRISPR KO gRNA 2 R	AAACGTCCTGAGTTTGATGTATCTC
SKP2 CRISPR KO gRNA 1 F	CACCGAAGACTTTGTGATTGTCCGC
SKP2 CRISPR KO gRNA 1 R	AAACGCGGACAATCACAAAGTCTTC
SKP2 CRISPR KO gRNA 2 F	CACCGTGGCAGACCTTAGACCTCAC
SKP2 CRISPR KO gRNA 2 R	AAACGTGAGGTCTAAGGTCTGCCAC
TCAF1 CRISPR KO gRNA 1 F	CACCGAGCACCGGTTAATTACGAGT
TCAF1 CRISPR KO gRNA 1 R	AAACACTCGTAATTAACCGGTGCTC
TCAF1 CRISPR KO gRNA 2 F	CACCGCCTTTGGCCAAAATCCTCGA
TCAF1 CRISPR KO gRNA 2 R	AAACTCGAGGATTTTGGCCAAAGGC
PTPN14 CRISPR KO gRNA 1 F	CACCGATTACGATGTACATTGGACC
PTPN14 CRISPR KO gRNA 1 R	AAACGGTCCAATGTACATCGTAATC
PTPN14 CRISPR KO gRNA 2 F	CACCGTGTGCTTACCGTGTGAAAGA
PTPN14 CRISPR KO gRNA 2 R	AAACTCTTTCACACGGTAAGCACAC
TET2 CRISPR KO gRNA 1 F	CACCGGATTCCGCTTGGTGAAAACG
TET2 CRISPR KO gRNA 1 R	AAACCGTTTTCACCAAGCGGAATCC
TET2 CRISPR KO gRNA 2 F	CACCGGTTCCAAAAACCCTCACACC
TET2 CRISPR KO gRNA 2 R	AAACGGTGTGAGGGTTTTTGGAACC

### Lentivirus production

HEK293T cells were expanded in fibroblast medium [DMEM (Millipore Sigma, #SLM-021-B) supplemented with 10% FBS, 1X GlutaMAX (Gibco, 35050), and 1% penicillin-streptomycin]. 1 day prior to transfection, 1/10 of the cells in a confluent T150 flask were plated into a 10 cm cell culture dish. Transfection was performed using FuGENE 6 (Promega, E2691) according to manufacturer’s protocol. Briefly, 30 μL FuGENE 6 was added to 400 μL DMEM/F12, which was then mixed with psPAX2 (Addgene, #12260, 3.75 μg), pMD2.G (Addgene, #12259, 1.25 μg), and the donor vector (5 μg). The mixture was incubated at room temperature for 20 min, during which time fresh fibroblast medium without penicillin-streptomycin was changed on the HEK293T cells. Following incubation, the mixture was added dropwise to the HEK293T cells and mixed. The cells were then incubated at 37 °C in 5% CO_2_ and 20% O_2_ overnight. The morning of day 1 after transfection, the media were replaced with fibroblast medium with penicillin-streptomycin. Lentivirus was harvested on the mornings of day 2 and 3 after transfection.

### CRISPR screen

Human Brunello CRISPR knockout pooled library lentiviral prep was a gift from David Root and John Doench (Addgene, #73179-LV). Prior to the screen, the transduction efficiency of the lentiviral prep was experimentally determined using BT5 hTSCs. A puromycin (Sigma, P8833) kill curve was also performed on BT5 hTSCs, and it was determined that 2 μg/mL was the minimal concentration required to kill all the cells. Two biological replicates of BT5 hTSCs were separately transduced with the Brunello pooled library lentivirus at low transduction efficiency (<50%) in the presence of 0.5 μg/mL polybrene (Millipore Sigma, TR-1003-G). Enough cells were transduced to achieve a representation of at least 500 cells per sgRNA, taking into account the transduction efficiency. One day after transduction, the media were replaced with puromycin-containing fresh hTSC medium. Two days after puromycin selection, the cells were dissociated and counted. Some of these cells were frozen in liquid nitrogen for genomic DNA isolation to serve as the day 0 (control) timepoint, while others were seeded to begin the screen, each maintaining a representation of at least 500 cells per sgRNA. From this point on, the media were changed every 2 days, and the cells were passaged every 3 days, with cell counts taken at every passage to maintain sufficient representation. Cells at the day 6, 12, and 18 timepoints were also frozen in liquid nitrogen for genomic DNA isolation, again maintaining sufficient representation of 500 cells per sgRNA. Some cells at the day 18 timepoint (endpoint) were also subject to flow cytometry analysis to confirm their hTSC identity.

### Genomic DNA isolation and sequencing

Genomic DNA (gDNA) was isolated using the QIAamp DNA Blood Maxi Kit (Qiagen, 51192) per the manufacturer’s instructions. The gDNA concentrations were quantitated by Nanodrop. For PCR amplification, 252 μg of DNA was amplified per sample using the MyTaq Red Mix (Bioline, 25043). Reactions were set up in 384-well plates, using 200 ng of gDNA in 12 μL of reaction mix per well. Primers used for gDNA amplification were Fwd 5′ tgtggaaaggacgaaacacc 3′ and Rev 5′ cggactagccttattttaacttgc 3′. PCR cycling condition is as follows: an initial 2 min at 94 °C; followed by 15 s at 94 °C, 15 s at 56 °C, 30 s at 72 °C, for 30 cycles; and a final 5 min extension at 72 °C. P5/P7 primers were synthesized at Integrated DNA Technologies (IDT). PCR products were purified using the Agencourt AMPure XP SPRI beads (Beckman Coulter, A63880) according to the manufacturer’s instructions. Samples were sequenced on a NovaSeq S4 2 × 150 platform (Illumina).

Reads were counted by first searching for the CACCG sequence in the primary read file that appears in the lentiviral vector 5′ to all sgRNA inserts. The next 20 nucleotides are the sgRNA insert, which was then mapped to a reference file of all possible sgRNAs present in the library^106^. The read was then assigned to a specific sample according to the barcode included in the P7 primer.

### CRISPR screen analysis

Reads of every sgRNA in the Human CRISPR Knockout Pooled Library (Brunello)^23^ were calculated by using the MAGeCK count module (v0.5.9.2)^107^ as previously reported^108^. The sgRNAs reads counts of all genes were inputted into the BAGEL package (v.91)^25^ and “BAGEL-calc_foldchange.py” script was used to calculate the fold change (FC) at day 6, day 12, and day 18, against the baseline at day 0. Mean of the log2 Bayes Factors (BF), recall, precision, and FDR were further calculated by following the manual instruction of the “BAGEL.py pr” module of the BAGEL2 package^109^. The core essential and nonessential gene lists required for BAGEL were obtained from previous studies^24,26^. EGs in CRISPR screening were defined as the depleted genes at day 18 compared to the day 0 baseline, with FDR < 0.05. 2139 genes with reliable levels of expression (RPKM > 0) in BT5 hTSCs^10^ were considered as EGs in this study. GRGs were identified as described previously^21^. Briefly, read counts of each gene were deduced by summing up the read counts of corresponding sgRNAs. DESeq2 was used to identify the GRGs as those with enriched sgRNA read counts at day 18 compared to the day 0 baseline, with *p*_adj_ < 0.01 and log_2_FC > 1. 619 genes with reliable levels of expression (RPKM > 0) in BT5 hTSCs^10^ were considered as GRGs in this study. GENCODE human gene annotation (v27, hg38) was used to determine the genomic coordinates of EGs and GRGs in the human genome, and to identify their upstream and downstream neighboring genes.

### Individual candidate gene validation

A puromycin kill curve was performed on hTSCs derived from H9 naïve hPSCs, and it was determined that 1 μg/mL was the minimal concentration required to kill all the cells. hTSCs were transduced with lentivirus expressing Cas9 and a single targeting (cloned as described above) or control (Addgene, #107402) sgRNA. One day after transduction, the media were replaced with puromycin-containing fresh hTSC medium. Two days after puromycin selection, the cells were dissociated and counted. For each condition, 50,000 cells were seeded per well in 6 wells. This is considered day 0 of the validation experiment, and media were replaced every 2 days. The total number of live cells in each well were counted on day 4 and/or day 6 of the experiment, with 3 wells (biological triplicates) counted at each timepoint. Trypan Blue was used to exclude dead cells. Two-tailed student’s t-test was used for statistical analysis. Additionally, cells were analyzed by NGS for presence of indels around the cut site at day 0, 6, 12, and 18 of the validation experiments.

### Gene Ontology, pathway, and cellular compartment analysis

The Gene Ontology (GO) analysis and pathway analysis of EGs and GRGs were performed by using ToppFun^110^. Protein localization data from Subcellular Localization Database (http://compartments.jensenlab.org/About)^111^ were used to determine the subcellular localization/compartment of identified EGs and GRGs. Gene-compartment assignment was determined by using the maximal confidence score method as described previously^22^. Genes that have the same maximal confidence scores in multiple compartments were assigned to each compartment independently. Hypergeometric test was further used to calculate the statistical significance of the enrichment/depletion of EGs and GRGs in each cellular compartment.

### Re-analysis of published transcriptomic data

Gene-level expression data (FPKM) of the CTB, EVT, PrE, EPI, and STB were obtained from a published study of human embryos cultured through implantation stages in vitro (GEO accession: GSE136447)^37^. The expression of select EGs and GRGs was obtained to generate the expression heatmaps and dot-plots in distinct embryonic and extraembryonic cell types at different developmental stages.

Gene-level RNA-seq expression data (reads counts) of cultured STB, EVT, hTSC, and naïve hPSC were downloaded from our prior study (accession: GSE138688)^10^. DEGs (hTSC vs naïve hPSC, hTSC vs STB, hTSC vs EVT) were identified using DESeq2 with criteria as absolute log_2_FC > 1, *p*_adj_ < 0.01, and CPM > 1 of libraries of either condition. To generate PCA plots, the genes with low expression (CPM < 1) in all libraries were removed to ensure high specificity. The transcription factors in EGs were confirmed by using the human TF list retrieved from Animal TFDB 3.0 (http://bioinfo.life.hust.edu.cn/AnimalTFDB/)^112^.

Gene-level single-cell RNA-seq data (FPKM) of human first trimester maternal-fetal interface were obtained through EMBL-EBI ArrayExpress (accession: EMTAB6678 and EMTAB6701)^91^. UMAP visualization of processed scRNA-seq data was generated with the coordinates of cell barcodes provided by the previous study^91^.

Gene-level single nuclei RNA-seq expression data (reads counts) of mouse placental labyrinth development from all nuclei or subclustered trophoblast nuclei were downloaded from a previous study (accession: GSE152248). The expression of selected hTSC EGs and GRGs was obtained to generate boxplots and violin plots across distinct cell types.

### Flow cytometry

Cells were single-cell dissociated using TrypLE Express and washed once in FACS buffer [PBS supplemented with 5% FBS]. The cells were then resuspended in 100 μL fresh FACS buffer, and incubated with antibodies for 30 min on ice. The following antibodies were used: anti-SUSD2-PE, 1:100 (BioLegend, 327406); anti-CD75-eFluor 660, 1:100 (Thermo Fisher, 50-0759-42); anti-ITGA6-FITC, 1:100 (Miltenyi, 130-097-245); anti-EGFR-APC, 1:20 (BioLegend, 352905); anti-Annexin V-FITC, 1:10 (Thermo Fisher, BMS147FI). Following antibody incubation, the cells were washed once with FACS buffer, resuspended in fresh FACS buffer, and passed through a cell strainer. Unstained cells that have undergone the same procedures were used as controls. Cell cycle analysis was performed using the Vybrant DyeCycle Violet Ready Flow Reagent (Thermo Fisher, R37172) according to the manufacturer’s instructions. 100 µM Verapamil (Sigma-Aldrich, V4629) was added during incubation to prevent dye efflux. Flow cytometry was performed using a BD LSRFortessa X-20 and the data were analyzed using the FACSDiva v9.0 or FlowJo v10.8.1 software.

### Immunofluorescence staining

Immunostaining was performed directly in the wells. Cells were fixed with 4% paraformaldehyde for 20 min at room temperature, then washed with PBS 3 times. The cells were permeabilized with 0.1% Triton X-100 (Sigma, T8787) in PBS for 5 min, then blocked with blocking buffer [PBS supplemented with 0.5% BSA and 0.1% Triton X-100] for 1 h. Cells were incubated with the primary antibody diluted in the blocking buffer overnight at 4 °C. The following primary antibody was used: anti-TEAD1, 1:100 (Cell Signaling Technology, 12292S). The cells were washed 3 times in PBS, then incubated with the secondary antibody diluted in blocking buffer for 1 h at room temperature. The following secondary antibody was used: donkey anti-rabbit-Alexa 647, 1:500 (Invitrogen, A-31573). The nuclei were stained with Hoechst 33258 (Thermo Fisher, H3569). Cells were washed 3 times in PBS, then imaged with a Leica DMi-8 fluorescence microscope. Some images were globally adjusted for brightness and/or contrast.

### Western blotting

Cells were harvested and resuspended in radioimmunoprecipitation assay (RIPA) buffer, then incubated on ice for 20 min. Whole cell extract concentration was measured by Bradford assay (Pierce, 23236). Proteins were balanced and subject to SDS-PAGE analysis. Primary antibodies used were TEAD1 (1:1000, Cell Signaling Technology, 12292S) and beta-actin (1:1000, Cell Signaling Technology, 4970). The membrane was imaged using the Thermo Fisher iBright Imaging System.

### Quantitative real-time PCR

Total RNA was isolated using the E.Z.N.A. total RNA kit I (Omega, D6834), and cDNA synthesis was performed from total RNA using the high capacity cDNA reverse transcription kit (Applied Biosystems, 4368814). Real-time PCR was performed using PowerUp SYBR green master mix (Applied Biosystems, A25743) on the StepOnePlus Real-Time PCR System (Applied Biosystems). All analyses were done in triplicate. Gene expression was normalized to RPLP0. Error bars represent the standard error (SE) of the mean of triplicate reactions. Two-tailed student’s t-test was performed for statistical analysis. Primer sequences are included in the following primer table:

**Table:** 

Gene	Primer sequence (5′− 3′)
RPLP0-F	GCTTCCTGGAGGGTGTCC
RPLP0-R	GGACTCGTTTGTACCCGTTG
SKP2-F	ATGCCCCAATCTTGTCCATCT
SKP2-R	CACCGACTGAGTGATAGGTGT
TEAD1-F	CCTGGCTATCTATCCACCATGTG
TEAD1-R	TTCTGGTCCTCGTCTTGCCTGT
TCAF1-F	TTGCCCACAGAAAATGTTGA
TCAF1-R	CAGATAGGCCAGGCTGGTAG
PTPN14-F	CGACTTCTGGCAGATGGTGT
PTPN14-R	GTGGCTTTTGGTTCGTCCAC
TET2-F	CAGCACATTGGTATGCACTC
TET2-R	TTTCCTTTGTCGGCAAGTCT

### Matrigel invasion assay

In vitro invasion assay was performed in Matrigel-coated transwell inserts with 8.0 μm pores (Corning, 354480). EVTs were single cell dissociated, and seeded at a density of 2 × 10^5^ cells per well into the upper chamber of Matrigel-coated transwells in 200 μL EVT basal medium. The lower chamber was filled with 800 μL of the same type of medium containing 20% FBS. Cells were cultured at 37 °C in 5% CO_2_ and 20% O_2_. After 36 h, cells in the upper chamber were carefully removed with a cotton swab. The lower chamber was fixed with 4% paraformaldehyde, washed with PBS, and then stained with crystal violet. Invaded cells were imaged on a Leica DMi1 microscope. Thereafter, the stained cells from five random fields were counted to calculate the relative fold change in the number of invading cells. One-tailed student’s t-test was performed for statistical analysis.

### RNA-seq

Total RNA was isolated using the E.Z.N.A. total RNA kit I. Library construction was performed using the SMARTer Directional cDNA Library Construction Kit (Clontech, 634933). Libraries were sequenced on an Illumina NovaSeq S4 2 × 150 platform. The data were processed as previously described^10^. Raw reads of RNA-seq libraries were processed using the RNA-seq pipeline, which consists of data processing, quality control, integrative analysis, and data visualization. In this pipeline, RNA-seq reads were aligned to the human genome hg38 with STAR version 2.5.4b^113^. Gene counts were derived from the number of uniquely aligned unambiguous reads by Subread:featureCount (version 1.4.6)^114^, with hg38 gene annotation ENCODE V27^115^. Unwanted variables among gene counts were removed using RUVr function of RUVSeq normalization package^116^, with the estimated number of factors of unwanted variation *k* as 1. Then the normalized gene counts were imported into the R/Bioconductor package DESeq2^117^. Genes with CPM > 1.0 were converted into a DESeq2 dataset and then regularized log-transformed using the rlog function from the DESeq2 package. Adjusted *p*-values for DEGs were determined by DESeq2 using the R stats function p.adjust with the Benjamini and Hochberg correction to determine the false discovery rate. Absolute log2(fold change of expression change) >0.5 and adjusted *p*-value< 0.05 were required to consider a gene as differentially expressed. Lists of WT vs. *TEAD1* KO DEGs could be found in Supplementary Data 4.

### ATAC-seq

ATAC-seq was performed as previously described with minor modifications^118^. Cells were harvested by TrypLE Express dissociation and centrifuged at 500 × *g* for 5 min at 4 °C. The supernatant was aspirated. Cells were washed once with cold PBS containing 0.04% BSA. Cell pellets of hTSC and EVT were resuspended in 300 μL DNase I (Thermo Fisher, EN0521) solution [20 mM Tris pH 7.4, 150 mM NaCl, 1X reaction buffer with MgCl_2_, 0.1 U/μL DNase I] on ice for 5 min. After DNase I treatment, 1 mL PBS containing 0.04% BSA was added and cells were centrifuged at 500 × *g* for 5 min at 4 °C. Another two washes were performed. Cell pellets of DNase I treated hTSC, EVT, and non-DNase I treated STB were then lysed in 100 µL ATAC-seq RSB [10 mM Tris pH 7.4, 10 mM NaCl, 3 mM MgCl_2_] containing 0.1% NP40, 0.1% Tween-20, and 0.01% Digitonin by pipetting up and down and incubating on ice for 3 min (hTSC and EVT) or 10 min (STB). 1 mL of ATAC-seq RSB containing 0.1% Tween-20 was added and mixed with the lysis reaction. The STB cells were then filtered through a 30 μm cell strainer. Nuclei were then pelleted by centrifuging at 600 × *g* for 5 min at 4 °C. Supernatant was removed, and the nuclear pellets were resuspended in 20 µL 2× TD buffer [20 mM Tris pH 7.6, 10 mM MgCl_2_, 20% Dimethyl Formamide]. Nuclei were counted, and 50,000 counted nuclei were then transferred to a tube with 2× TD buffer filled up to 25 µL. 25 µL of transposition mix [2.5 µL Transposase (100 nM final) (Illumina, 20034197, 16.5 µL PBS, 0.5 µL 1% Digitonin, 0.5 µL 10% Tween-20, 5 µL H2O) was then added. Transposition reactions were mixed and incubated at 37 °C for 30 min with gentle tapping every 10 min. Reactions were cleaned up with the Zymo DNA Clean and Concentrator-5 kit (Zymo Research, D4014). The ATAC-seq library was prepared by amplifying for 9 cycles on a PCR machine. The PCR reaction was purified with Ampure XP beads using double size selection following the manufacturer’s protocol, in which 27.5 µL beads (0.55X sample volume) and 50 µL beads (1.55X sample volume) were used based on 50 µL PCR reaction. The ATAC-seq libraries were quantitated by Qubit assays and sequenced on an Illumina NextSeq 500 platform. Data analysis was performed on data generated in this study and ATAC-seq data that we previously generated from hTSCs derived from naïve hPSCs^10^ (GSE138761). The data were processed as previously described^119^. Raw reads of ATAC-seq libraries were aligned to human reference genome (hg38) and further processed by using AIAP^119^, which consists of four steps: data processing, quality control, integrative analysis, and data visualization. The narrow peak files of all ATAC-seq libraries were further merged by using the merge function of BEDTools suite^120^, and the read counts of each ATAC-seq peak were quantified by using BEDTools coverage command. hTSC-specific and hPSC-specific DARs were identified by using DESeq2^117^ with the cutoff as *p*_adj_ < 1e−5 and absolute log2(fold change)>2 as previously described^10^. Batch effects among read counts under ATAC-seq peaks of the WT and *TEAD1* KO hTSC, EVT, and STB samples were removed using RUVr function of the RUVseq R package, with the estimated number of factors of unwanted variation *k* as 1. Cell type-specific DARs were identified by DESeq2 with the cutoff as *p*_adj_ < 0.01 and absolute log2(fold change)>1. *TEAD1* KO-specific and WT-specific DARs were identified among hTSCs, EVTs and STBs using DESeq2 with the cutoff as *p*_adj_ < 0.05 and absolute log2(fold change) > 0.5. GREAT tool (v4.0.4) was used to identify the enriched GO terms and pathways of the genes around *TEAD1* KO-specific and WT-specific DARs.

### CUT&Tag

CUT&Tag was performed as previously described^61^. Briefly, 200,000 cells per sample were washed in Wash Buffer [1 mL 1 M HEPES pH 7.5 (Sigma Aldrich, H3375), 1.5 mL 5 M NaCl (Sigma Aldrich, S5150), 12.5 μL 2 M Spermidine (Sigma Aldrich, S2501), 1 Roche Complete Protease Inhibitor EDTA-Free tablet (Sigma Aldrich, 5056489001), and bring the final volume to 50 mL with dH_2_O], captured with Concanavalin A beads (Bangs Laboratories, BP531), and incubated with primary antibody at 4 °C overnight. The cells were then incubated with secondary antibody at room temperature for an hour. The following antibodies were used: TEAD1 (1:100, Cell Signaling Technology, 12292S); guinea pig-anti-rabbit (1:100, Antibodies online, ABIN101961). After washing off the unbound antibodies with Dig-Wash Buffer [mix 400 μL 5% Digitonin (EMD Millipore, 300410) with 40 mL Wash Buffer], the pA-Tn5 adapter complex (kindly provided by Dr. Steve Henikoff) was added at 1:200 and incubated at room temperature for an hour. The cells were washed again with Dig-300 Buffer [1 mL 1 M HEPES pH 7.5, 3 mL 5 M NaCl, 12.5 μL 2 M Spermidine, 100 μL 5% Digitonin, 1 Roche Complete Protease Inhibitor EDTA-Free tablet, and bring the final volume to 50 mL with dH_2_O], and incubated in 300 μL Tagmentation Buffer [5 mL Dig-300 Buffer and 50 μL 1 M MgCl_2_ (Sigma Aldrich, M8266)] per sample at 37 °C for an hour. The reaction was stopped and the DNA solubilized by adding 10 μL 0.5 M EDTA (Research Organics, 3002E), 3 μL 10% SDS (Sigma Aldrich, L4509), and 2.5 μL Proteinase K (Thermo Fisher, EO0492) per sample, and incubated at 50 °C for an hour. The DNA was then extracted, excess RNA digested with RNase A (Thermo Fisher, EN0531), and PCR amplified with i5 and i7 indexing primers. The PCR product was cleaned up with AMPure XP beads, and the size distribution and concentration were confirmed using Tapestation. The libraries were then sequenced on an Illumina NovaSeq S4 2 × 150 platform.

TEAD1 CUT&Tag raw data of each biological replicate were processed with the human reference genome hg38 and further processed by using AIAP(v1.1)^119^, which consists of four steps: data processing, quality control, integrative analysis, and data visualization. The enriched TEAD1 peaks were identified by using MACS2 peak calling function with the *q*-value cutoff 1e−5. The peak files of all replicates were merged by using the merge function of BEDTools suite. Only the TEAD1 peaks identified in at least 2 replicates were considered as high-quality binding sites and were further assigned to their nearest neighboring genes for the downstream analysis.

HOMER (v4.11.1)^121^ was used to calculate the motif enrichment (Supplementary Data 5) and genomic enrichment under the high-quality TEAD1 binding regions. GREAT tool (v4.0.4)^122^ was used to identify the enriched GO terms and pathways of the genes around TEAD1 binding peaks in human hTSCs.

ATAC-seq signals on TEAD1 binding regions were calculated by using deepTools^123^ with parameter detailed as “computeMatrix reference-point—referencePoint center -a 5000 -b 5000 -bs 100 –missingDataAsZero”, and the averaged ATAC-seq signals were subsequently plotted by using plotHeatmap in deepTools package.

### Reporting summary

Further information on research design is available in the Nature Research Reporting Summary linked to this article.

## Supplementary information

Supplementary Information

Editorial Assessment Report

List of Supplementary Data

Supplementary Data 1

Supplementary Data 2

Supplementary Data 3

Supplementary Data 4

Supplementary Data 5

Reporting Summary

## Data Availability

The data that support this study are available from the corresponding authors upon reasonable request. The CRISPR screen sequencing data, TEAD1 CUT&Tag data, naïve hPSC ATAC-seq data, and the WT and *TEAD1* KO hTSC, EVT, and STB RNA-seq and ATAC-seq data generated in this study are available under the GEO accession number GSE172329. The WT primed hPSC, naïve hPSC, hTSC, EVT, and STB RNA-seq data and the WT hTSC ATAC-seq data were retrieved from GSE138762; the human embryo scRNA-seq data was retrieved from GSE136447; the human maternal-fetal interface scRNA-seq data was retrieved from E-MTAB-6701 and E-MTAB-6678; the mouse placenta snRNA-seq data was retrieved from GSE152248; the human reference genome was retrieved from GENCODE v27. Source data are provided with this paper.

## Source data

Source Data

## Code availability

All custom scripts used in this study are deposited in https://github.com/Zhang-lab/CRISPR/.

## References

[1] Shahbazi MN, Zernicka-Goetz M (2018). Deconstructing and reconstructing the mouse and human early embryo. Nat. Cell Biol..

[2] James JL, Carter AM, Chamley LW (2012). Human placentation from nidation to 5 weeks of gestation. Part I: What do we know about formative placental development following implantation?. Placenta.

[3] Burton Graham J, Fowden Abigail L (2015). The placenta: A multifaceted, transient organ. Philos. Trans. R. Soc. B: Biol. Sci..

[4] Burton GJ, Woods AW, Jauniaux E, Kingdom JC (2009). Rheological and physiological consequences of conversion of the maternal spiral arteries for uteroplacental blood flow during human pregnancy. Placenta.

[5] Norwitz ER (2006). Defective implantation and placentation: Laying the blueprint for pregnancy complications. Reprod. BioMed. Online.

[6] Moffett A, Loke C (2006). Immunology of placentation in eutherian mammals. Nat. Rev. Immunol..

[7] Koot YE, Teklenburg G, Salker MS, Brosens JJ, Macklon NS (2012). Molecular aspects of implantation failure. Biochim. Biophys. Acta.

[8] Okae H (2018). Derivation of human trophoblast stem cells. Cell Stem Cell.

[9] Io, S et al. Capturing human trophoblast development with naive pluripotent stem cells in vitro. *Cell Stem Cell* **28**, 1023–1039.e13 (2021). 10.1016/j.stem.2021.03.013 33831365

[10] Dong, C et al. Derivation of trophoblast stem cells from naive human pluripotent stem cells. *Elife* **9**, e52504 (2020). 10.7554/eLife.52504 PMC7062471 32048992

[11] Liu X (2020). Reprogramming roadmap reveals route to human induced trophoblast stem cells. Nature.

[12] Cinkornpumin JK (2020). Naive human embryonic stem cells can give rise to cells with a trophoblast-like transcriptome and methylome. Stem Cell Rep..

[13] Castel G (2020). Induction of human trophoblast stem cells from somatic cells and pluripotent stem cells. Cell Rep..

[14] Koike-Yusa H, Li Y, Tan EP, Velasco-Herrera MDC, Yusa K (2014). Genome-wide recessive genetic screening in mammalian cells with a lentiviral CRISPR-guide RNA library. Nat. Biotechnol..

[15] Chen S (2015). Genome-wide CRISPR screen in a mouse model of tumor growth and metastasis. Cell.

[16] Hart T (2015). High-resolution CRISPR screens reveal fitness genes and genotype-specific cancer liabilities. Cell.

[17] Shalem O (2014). Genome-scale CRISPR-Cas9 knockout screening in human cells. Science.

[18] Wang T, Wei JJ, Sabatini DM, Lander ES (2014). Genetic screens in human cells using the CRISPR-Cas9 system. Science.

[19] Ihry RJ (2019). Genome-scale CRISPR screens identify human pluripotency-specific genes. Cell Rep..

[20] Mair B (2019). Essential gene profiles for human pluripotent stem cells identify uncharacterized genes and substrate dependencies. Cell Rep..

[21] Yilmaz A, Peretz M, Aharony A, Sagi I, Benvenisty N (2018). Defining essential genes for human pluripotent stem cells by CRISPR-Cas9 screening in haploid cells. Nat. Cell Biol..

[22] Yilmaz A, Braverman-Gross C, Bialer-Tsypin A, Peretz M, Benvenisty N (2020). Mapping gene circuits essential for germ layer differentiation via loss-of-function screens in haploid human embryonic stem cells. Cell Stem Cell.

[23] Doench JG (2016). Optimized sgRNA design to maximize activity and minimize off-target effects of CRISPR-Cas9. Nat. Biotechnol..

[24] Hart T, Brown KR, Sircoulomb F, Rottapel R, Moffat J (2014). Measuring error rates in genomic perturbation screens: gold standards for human functional genomics. Mol. Syst. Biol..

[25] Hart T, Moffat J (2016). BAGEL: A computational framework for identifying essential genes from pooled library screens. BMC Bioinform..

[26] Hart T (2017). Evaluation and design of genome-wide CRISPR/SpCas9 knockout screens. G3: Genes|Genomes|Genet..

[27] Auman HJ (2002). Transcription factor AP-2γ is essential in the extra-embryonic lineages for early postimplantation development. Development.

[28] Werling U, Schorle H (2002). Transcription factor gene AP-2γ essential for early murine development. Mol. Cell. Biol..

[29] Biadasiewicz K (2011). Transcription factor AP-2α promotes EGF-dependent invasion of human trophoblast. Endocrinology.

[30] Kuckenberg P, Kubaczka C, Schorle H (2012). The role of transcription factor Tcfap2c/TFAP2C in trophectoderm development. Reprod. BioMed. Online.

[31] Krendl C (2017). GATA2/3-TFAP2A/C transcription factor network couples human pluripotent stem cell differentiation to trophectoderm with repression of pluripotency. Proc. Natl Acad. Sci. USA.

[32] Yagi R (2007). Transcription factor TEAD4 specifies the trophectoderm lineage at the beginning of mammalian development. Development.

[33] Nishioka N (2009). The Hippo signaling pathway components Lats and Yap pattern Tead4 activity to distinguish mouse trophectoderm from inner cell mass. Dev. Cell.

[34] Nishioka N (2008). Tead4 is required for specification of trophectoderm in pre-implantation mouse embryos. Mech. Dev..

[35] Saha B (2020). TEAD4 ensures postimplantation development by promoting trophoblast self-renewal: An implication in early human pregnancy loss. Proc. Natl Acad. Sci. USA.

[36] Chi F, Sharpley MS, Nagaraj R, Roy SS, Banerjee U (2020). Glycolysis-independent glucose metabolism distinguishes TE from ICM fate during mammalian embryogenesis. Dev. Cell.

[37] Xiang L (2020). A developmental landscape of 3D-cultured human pre-gastrulation embryos. Nature.

[38] Nichols J, Smith A (2009). Naive and primed pluripotent states. Cell Stem Cell.

[39] Knöfler M, Mösl B, Bauer S, Griesinger G, Husslein P (2000). TNF-α/TNFRI in primary and immortalized first trimester cytotrophoblasts. Placenta.

[40] Rhee C (2017). ARID3A is required for mammalian placenta development. Dev. Biol..

[41] Home P (2017). Genetic redundancy of GATA factors in the extraembryonic trophoblast lineage ensures the progression of preimplantation and postimplantation mammalian development. Development.

[42] Yamauchi Y (2020). Skp2 contributes to cell cycle progression in trophoblast stem cells and to placental development. Genes Cells.

[43] Gerri, C et al. Initiation of a conserved trophectoderm program in human, cow, and mouse embryos. *Nature* **587**, 443–447 (2020). 10.1038/s41586-020-2759-x PMC7116563 32968278

[44] Wang Y (2002). [Expression of lethal gene mRNA on placenta villi in patients with spontaneous abortion]. Zhonghua Fu Chan Ke Za Zhi.

[45] Theunissen TW (2016). Molecular criteria for defining the naive human pluripotent state. Cell Stem Cell.

[46] Theunissen TW (2014). Systematic identification of culture conditions for induction and maintenance of naive human pluripotency. Cell Stem Cell.

[47] Collier AJ (2017). Comprehensive cell surface protein profiling identifies specific markers of human naive and primed pluripotent states. Cell Stem Cell.

[48] Bredenkamp, N., Stirparo, G. G., Nichols, J., Smith, A. & Guo, G. The cell-surface marker Sushi Containing Domain 2 facilitates establishment of human naive pluripotent stem cells. *Stem Cell Rep.* **12**, 1212–1222 (2019). 10.1016/j.stemcr.2019.03.014 PMC6565611 31031191

[49] Doneda L, Bulfamante G, Grimoldi MG, Volpi L, Larizza L (1997). Localization of fos, jun, kit and SCF mRNA in human placenta throughout gestation using in situ RT-PCR. Early Pregnancy.

[50] Renaud SJ, Kubota K, Rumi MA, Soares MJ (2014). The FOS transcription factor family differentially controls trophoblast migration and invasion. J. Biol. Chem..

[51] Ji J (2020). Fibronectin 1 inhibits the apoptosis of human trophoblasts by activating the PI3K/Akt signaling pathway. Int. J. Mol. Med..

[52] Tilburgs T (2015). Human HLA-G+ extravillous trophoblasts: Immune-activating cells that interact with decidual leukocytes. Proc. Natl Acad. Sci. USA.

[53] Ueno M (2013). c-Met-dependent multipotent labyrinth trophoblast progenitors establish placental exchange interface. Dev. Cell.

[54] Xu X-H (2019). Downregulation of lysyl oxidase and lysyl oxidase-like protein 2 suppressed the migration and invasion of trophoblasts by activating the TGF-β/collagen pathway in preeclampsia. Exp. Mol. Med..

[55] Zhao B (2011). Angiomotin is a novel Hippo pathway component that inhibits YAP oncoprotein. Genes Dev..

[56] Chrysanthou S (2018). A critical role of TET1/2 proteins in cell-cycle progression of trophoblast stem cells. Stem Cell Rep..

[57] Imakawa K (2016). CITED2 modulation of trophoblast cell differentiation: Insights from global transcriptome analysis. Reproduction.

[58] Hanna CW, Kelsey G (2014). The specification of imprints in mammals. Heredity.

[59] Waclawik A, Ziecik AJ (2007). Differential expression of prostaglandin (PG) synthesis enzymes in conceptus during peri-implantation period and endometrial expression of carbonyl reductase/PG 9-ketoreductase in the pig. J. Endocrinol..

[60] Lin KC, Park HW, Guan KL (2017). Regulation of the Hippo pathway transcription factor TEAD. Trends Biochem. Sci..

[61] Kaya-Okur HS (2019). CUT&Tag for efficient epigenomic profiling of small samples and single cells. Nat. Commun..

[62] Guo, G. et al. Human naïve epiblast cells possess unrestricted lineage potential. *Cell Stem Cell* **28**, 1040–1056.e6 (2021). 10.1016/j.stem.2021.02.025 PMC8189439 33831366

[63] Kubota K, Kent LN, Rumi MAK, Roby KF, Soares MJ (2015). Dynamic regulation of AP-1 transcriptional complexes directs trophoblast differentiation. Mol. Cell. Biol..

[64] Goldman-Wohl D (2004). Eph and ephrin expression in normal placental development and preeclampsia. Placenta.

[65] Fujiwara H (2013). Eph-ephrin A system regulates human choriocarcinoma-derived JEG-3 cell invasion. Int. J. Gynecol. Cancer.

[66] Yung HW (2008). Evidence of placental translation inhibition and endoplasmic reticulum stress in the etiology of human intrauterine growth restriction. Am. J. Pathol..

[67] Lamar E (2001). Nrarp is a novel intracellular component of the Notch signaling pathway. Genes Dev..

[68] Haider S, Pollheimer J, Knöfler M (2017). Notch signalling in placental development and gestational diseases. Placenta.

[69] Yang W, Li Q, Pan Z (2014). Sphingosine-1-phosphate promotes extravillous trophoblast cell invasion by activating MEK/ERK/MMP-2 signaling pathways via S1P/S1PR1 axis activation. PLoS One.

[70] Varberg, K. M. et al. ASCL2 reciprocally controls key trophoblast lineage decisions during hemochorial placenta development. *Proc. Natl Acad. Sci. USA* **118**, e2016517118 (2021). 10.1073/pnas.2016517118 PMC7958375 33649217

[71] Lee, C. Q. E. et al. Integrin α2 marks a niche of trophoblast progenitor cells in first trimester human placenta. *Development* **145**, dev162305 (2018). 10.1242/dev.162305 PMC6124543 29540503

[72] Azar C (2018). RNA-Seq identifies genes whose proteins are transformative in the differentiation of cytotrophoblast to syncytiotrophoblast, in human primary villous and BeWo trophoblasts. Sci. Rep..

[73] Zani A (2019). Interferon-induced transmembrane proteins inhibit cell fusion mediated by trophoblast syncytins. J. Biol. Chem..

[74] Cheung AN, Zhang HJ, Xue WC, Siu MK (2009). Pathogenesis of choriocarcinoma: Clinical, genetic and stem cell perspectives. Future Oncol..

[75] Mi S (2000). Syncytin is a captive retroviral envelope protein involved in human placental morphogenesis. Nature.

[76] Yu C (2002). GCMa regulates the syncytin-mediated trophoblastic fusion. J. Biol. Chem..

[77] Hughes M (2004). The Hand1, Stra13, and Gcm1 transcription factors override FGF signaling to promote terminal differentiation of trophoblast stem cells. Dev. Biol..

[78] Skonier J (1992). cDNA cloning and sequence analysis of beta ig-h3, a novel gene induced in a human adenocarcinoma cell line after treatment with transforming growth factor-beta. DNA Cell Biol..

[79] Wang W (2012). PTPN14 is required for the density-dependent control of YAP1. Genes Dev..

[80] Huang JM (2013). YAP modifies cancer cell sensitivity to EGFR and survivin inhibitors and is negatively regulated by the non-receptor type protein tyrosine phosphatase 14. Oncogene.

[81] Liu X (2013). PTPN14 interacts with and negatively regulates the oncogenic function of YAP. Oncogene.

[82] Ouseph MadhuM (2012). Atypical E2F repressors and activators coordinate placental development. Dev. Cell.

[83] Vallier L, Alexander M, Pedersen RA (2005). Activin/Nodal and FGF pathways cooperate to maintain pluripotency of human embryonic stem cells. J. Cell Sci..

[84] Russell R (2015). A dynamic role of TBX3 in the pluripotency circuitry. Stem Cell Rep..

[85] Niwa H, Ogawa K, Shimosato D, Adachi K (2009). A parallel circuit of LIF signalling pathways maintains pluripotency of mouse ES cells. Nature.

[86] Lv B (2019). Single-cell RNA sequencing reveals regulatory mechanism for trophoblast cell-fate divergence in human peri-implantation conceptuses. PLoS Biol..

[87] Cockburn K, Biechele S, Garner J, Rossant J (2013). The Hippo pathway member Nf2 is required for inner cell mass specification. Curr. Biol..

[88] Carter AM (2007). Animal models of human placentation—a review. Placenta.

[89] Roberts RM, Green JA, Schulz LC (2016). The evolution of the placenta. Reproduction.

[90] Perez-Garcia V (2018). Placentation defects are highly prevalent in embryonic lethal mouse mutants. Nature.

[91] Vento-Tormo R (2018). Single-cell reconstruction of the early maternal–fetal interface in humans. Nature.

[92] Marsh, B. & Blelloch, R. Single nuclei RNA-seq of mouse placental labyrinth development. *Elife* **9**, e60266 (2020). 10.7554/eLife.60266 PMC7669270 33141023

[93] Shi, J.-H. & Sun, S.-C. Tumor necrosis factor receptor-associated factor regulation of nuclear factor κB and mitogen-activated protein kinase pathways. *Front. Immunol.* **9**, 1849 (2018). 10.3389/fimmu.2018.01849 PMC6094638 30140268

[94] Antonicka H (2017). A pseudouridine synthase module is essential for mitochondrial protein synthesis and cell viability. EMBO Rep..

[95] Guarani V (2014). TIMMDC1/C3orf1 functions as a membrane-embedded mitochondrial complex I assembly factor through association with the MCIA complex. Mol. Cell Biol..

[96] Lee, R. G. et al. Cardiolipin is required for membrane docking of mitochondrial ribosomes and protein synthesis. *J. Cell Sci.* **133**, jcs240374 (2020). 10.1242/jcs.240374 32576663

[97] Kasahara T (2020). Cardiolipin is essential for early embryonic viability and mitochondrial integrity of neurons in mammals. Faseb J..

[98] Boukalova S (2020). Dihydroorotate dehydrogenase in oxidative phosphorylation and cancer. Biochim. Biophys. Acta Mol. Basis Dis..

[99] Houghton FD (2006). Energy metabolism of the inner cell mass and trophectoderm of the mouse blastocyst. Differentiation.

[100] Smith CL, Eppig JT (2009). The mammalian phenotype ontology: Enabling robust annotation and comparative analysis. Wiley Interdiscip. Rev. Syst. Biol. Med..

[101] Köhler S (2018). Expansion of the human phenotype ontology (HPO) knowledge base and resources. Nucleic Acids Res..

[102] Wu G, Bazer FW, Wallace JM, Spencer TE (2006). BOARD-INVITED REVIEW: Intrauterine growth retardation: Implications for the animal sciences1. J. Anim. Sci..

[103] Fischer LA, Khan SA, Theunissen TW (2022). Induction of human naive pluripotency using 5i/L/A medium. Methods Mol. Biol..

[104] Bredenkamp, N. et al. Wnt inhibition facilitates RNA-mediated reprogramming of human somatic cells to naive pluripotency. *Stem Cell Rep.* **13**, 1083–1098 (2019). 10.1016/j.stemcr.2019.10.009 PMC6915845 31708477

[105] Dong C, Theunissen TW (2022). Generating trophoblast stem cells from human naive pluripotent stem cells. Methods Mol. Biol..

[106] Sanson KR (2018). Optimized libraries for CRISPR-Cas9 genetic screens with multiple modalities. Nat. Commun..

[107] Li W (2014). MAGeCK enables robust identification of essential genes from genome-scale CRISPR/Cas9 knockout screens. Genome Biol..

[108] Li W (2015). Quality control, modeling, and visualization of CRISPR screens with MAGeCK-VISPR. Genome Biol..

[109] Kim E, Hart T (2021). Improved analysis of CRISPR fitness screens and reduced off-target effects with the BAGEL2 gene essentiality classifier. Genome Med..

[110] Chen J, Bardes EE, Aronow BJ, Jegga AG (2009). ToppGene Suite for gene list enrichment analysis and candidate gene prioritization. Nucleic Acids Res..

[111] Binder, J. X. et al. COMPARTMENTS: Unification and visualization of protein subcellular localization evidence. *Database* **2014**, bau012 (2014). 10.1093/database/bau012 PMC3935310 24573882

[112] Hu H (2019). AnimalTFDB 3.0: A comprehensive resource for annotation and prediction of animal transcription factors. Nucleic Acids Res..

[113] Dobin A (2012). STAR: Ultrafast universal RNA-seq aligner. Bioinformatics.

[114] Liao Y, Smyth GK, Shi W (2013). The Subread aligner: Fast, accurate and scalable read mapping by seed-and-vote. Nucleic Acids Res..

[115] Harrow J (2012). GENCODE: The reference human genome annotation for The ENCODE Project. Genome Res..

[116] Risso D, Ngai J, Speed TP, Dudoit S (2014). Normalization of RNA-seq data using factor analysis of control genes or samples. Nat. Biotechnol..

[117] Love MI, Huber W, Anders S (2014). Moderated estimation of fold change and dispersion for RNA-seq data with DESeq2. Genome Biol..

[118] Corces MR (2017). An improved ATAC-seq protocol reduces background and enables interrogation of frozen tissues. Nat. Methods.

[119] Liu, S. et al. AIAP: A quality control and integrative analysis package to improve ATAC-seq data analysis. *Genomics, Proteomics & Bioinformatics* **19**, 641–651 (2021). 10.1016/j.gpb.2020.06.025 PMC9040017 34273560

[120] Quinlan AR, Hall IM (2010). BEDTools: A flexible suite of utilities for comparing genomic features. Bioinformatics.

[121] Heinz S (2010). Simple combinations of lineage-determining transcription factors prime cis-regulatory elements required for macrophage and B cell identities. Mol. Cell.

[122] McLean CY (2010). GREAT improves functional interpretation of cis-regulatory regions. Nat. Biotechnol..

[123] Ramírez F (2016). deepTools2: A next generation web server for deep-sequencing data analysis. Nucleic Acids Res..

