# Supplementary material for: A genome-wide CRISPR-Cas9 knockout screen identifies essential and growth-restricting genes in human trophoblast stem cells
Source: Nat Commun. 2022 May 10;13:2548. doi: 10.1038/s41467-022-30207-9 (PMC9090837; doi:10.1038/s41467-022-30207-9)
Supplement: Supplementary file 1 — Supplementary Information [file 41467_2022_30207_MOESM1_ESM.pdf]

A genome-wide CRISPR-Cas9 knockout screen identifies essential and growth-restricting genes in human trophoblast stem cells

Chen Dong, Shuhua Fu, Rowan M. Karvas, Brian Chew, Laura A. Fischer, Xiaoyun Xing, Jessica Harrison, Pooja Popli, Ramakrishna Kommagani, Ting Wang, Bo Zhang, and Thorold W. Theunissen

Supplementary Information File

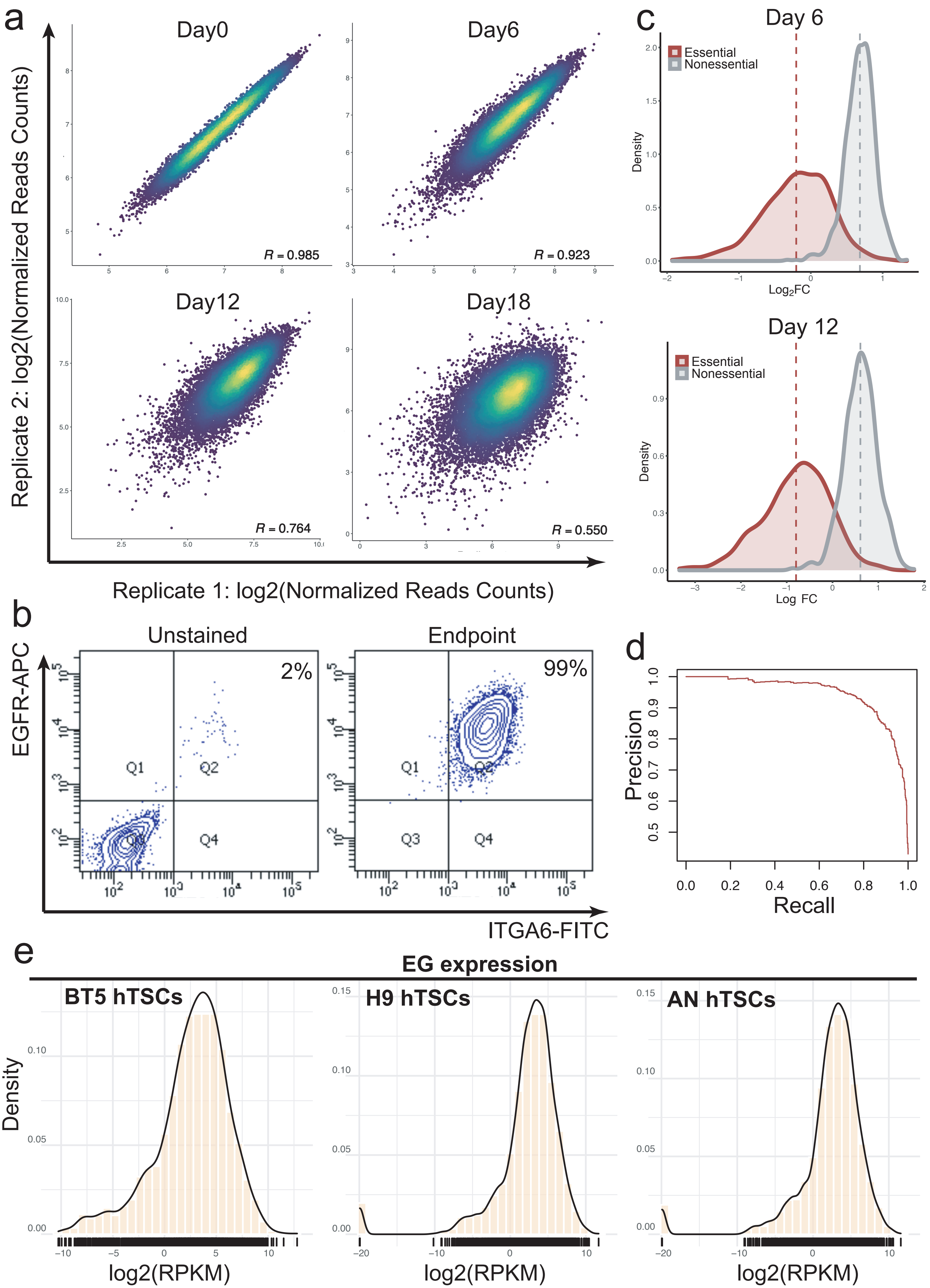
a
Day0
Day6
Day12
Day18
R = 0.985
R = 0.923
R = 0.764
R = 0.550
Replicate 2: log2(Normalized Reads Counts)
Replicate 1: log2(Normalized Reads Counts)
c
Day 6
Day 12
Essential
Nonessential
Density
Log2FC
b
Unstained
Endpoint
2%
99%
Q1
Q2
Q3
Q4
EGFR-APC
ITGA6-FITC
d
Precision
Recall
e
EG expression
BT5 hTSCs
H9 hTSCs
AN hTSCs
Density
log2(RPKM)

Supplementary Fig. 1: Quality control analyses of the CRISPR screen. a, Scatter plot of Log2 normalized read counts between the two biological replicate experiments at various timepoints (R=Pearson's correlation coefficient). **b**, Flow cytometry analysis for hTSC markers ITGA6 and EGFR in the second biological replicate of BT5 hTSCs following the CRISPR screen endpoint. **c**, Fold change distribution of sgRNAs targeting essential and nonessential genes[1] at day 6 and 12 of the screen. **d**, Precision-recall curve of the screen at the endpoint. Note that the data maintained a high precision (specificity) even at high recall (sensitivity), which is indicative of a successful screen. **e**, The mRNA expression distribution of all hTSC EGs in published BT5, H9, and AN hTSC RNA-seq data[2]. Note that the expression distribution of all hTSC EGs are similar regardless of genetic background.

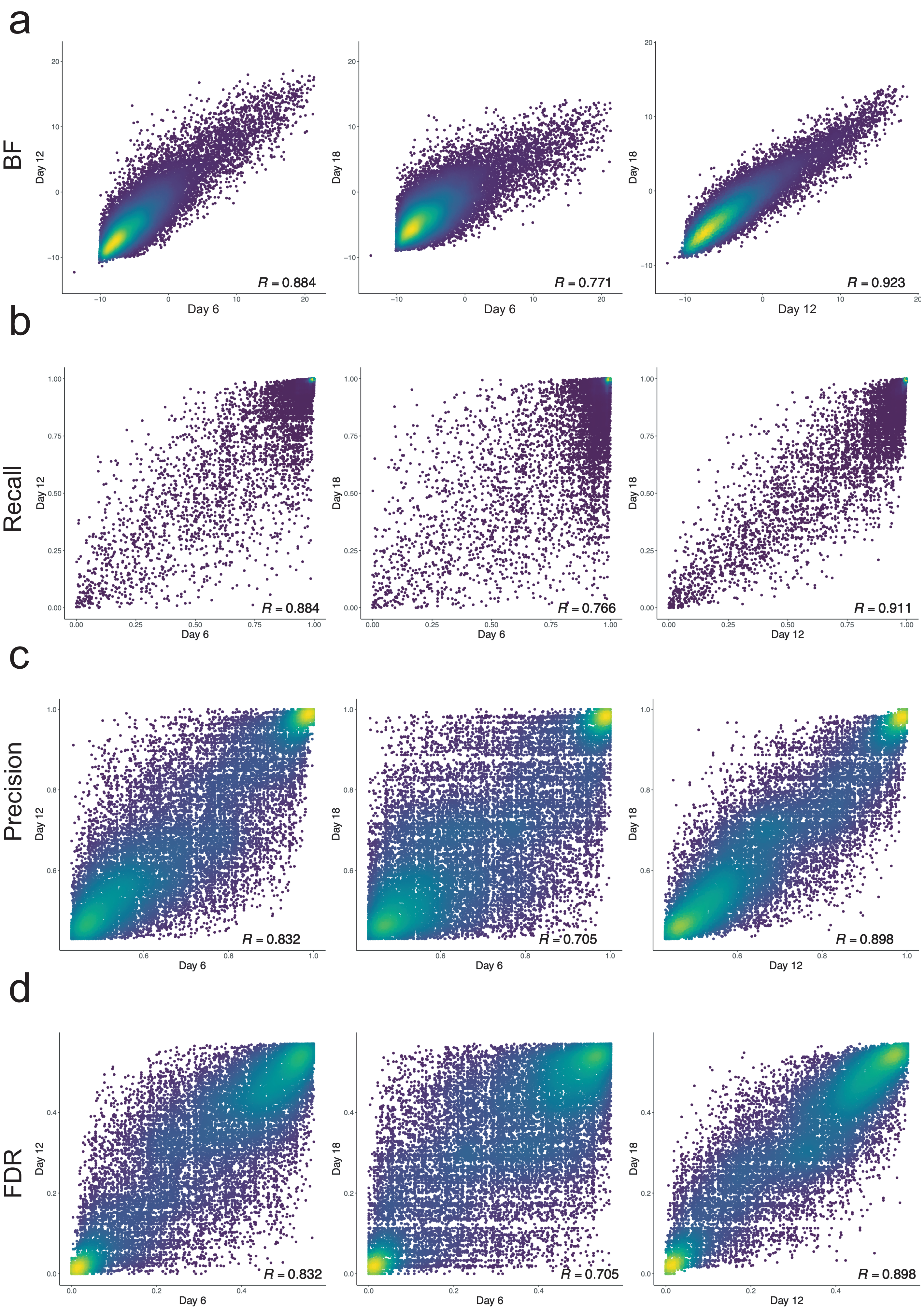

a
BF
Day 12
Day 6
R = 0.884
Day 18
Day 6
R = 0.771
Day 18
Day 12
R = 0.923
b
Recall
Day 12
Day 6
R = 0.884
Day 18
Day 6
R = 0.766
Day 18
Day 12
R = 0.911
c
Precision
Day 12
Day 6
R = 0.832
Day 18
Day 6
R = 0.705
Day 18
Day 12
R = 0.898
d
FDR
Day 12
Day 6
R = 0.832
Day 18
Day 6
R = 0.705
Day 18
Day 12
R = 0.898

Supplementary Fig. 2: Correlation between screening timepoints. Scatter plot of Bayes Factor (BF) (**a**), Recall value (**b**), Precision value (**c**), and false discovery rate (FDR) (**d**) between various screening timepoints (R=Pearson's correlation coefficient). Note the good correlation between adjacent time points.

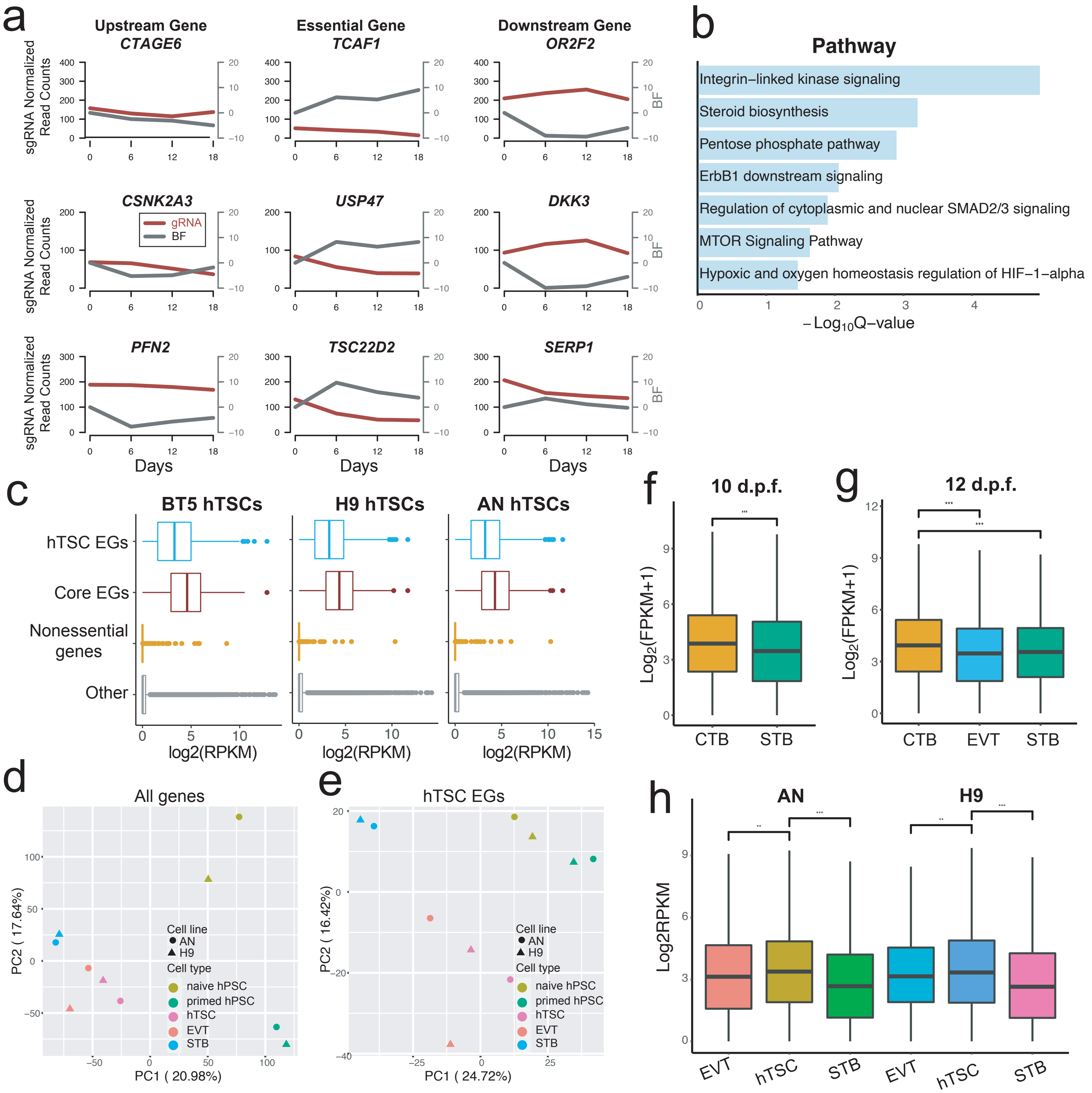
a
Upstream Gene
CTAGE6
Essential Gene
TCAF1
Downstream Gene
OR2F2
sgRNA Normalized Read Counts
BF
CSNK2A3
USP47
DKK3
gRNA
BF
PFN2
TSC22D2
SERP1
Days
b
Pathway
Integrin−linked kinase signaling
Steroid biosynthesis
Pentose phosphate pathway
ErbB1 downstream signaling
Regulation of cytoplasmic and nuclear SMAD2/3 signaling
MTOR Signaling Pathway
Hypoxic and oxygen homeostasis regulation of HIF−1−alpha
−Log10Q−value
c
BT5 hTSCs
H9 hTSCs
AN hTSCs
hTSC EGs
Core EGs
Nonessential genes
Other
log2(RPKM)
f
10 d.p.f.
Log2(FPKM+1)
CTB
STB
g
12 d.p.f.
Log2(FPKM+1)
CTB
EVT
STB
d
All genes
PC2 (17.64%)
PC1 (20.98%)
Cell line
AN
H9
Cell type
naive hPSC
primed hPSC
hTSC
EVT
STB
e
hTSC EGs
PC2 (16.42%)
PC1 (24.72%)
Cell line
AN
H9
Cell type
naive hPSC
primed hPSC
hTSC
EVT
STB
h
AN
H9
Log2RPKM
EVT
hTSC
STB
EVT
hTSC
STB

Supplementary Fig. 3: Characterization of hTSC EGs. a, The mean sgRNA normalized read counts and BFs of selected hTSC EGs and their neighboring genes over time. Results are representative of two independent experiments. **b**, Selected pathways significantly enriched among hTSC EGs. **c**, Expression levels of hTSC EGs, core EGs[1], nonessential genes[1], and all other genes not in the above three categories in BT5, H9, and AN hTSC RNA-seq data[2]. Two independent samples per cell line were used for analysis. Boxplot presents the 25th, median, and 75th quartiles, the whiskers extend 1.5 of interquartile ranges, and the dots are outside values >1.5 times and <3 times the interquartile range beyond either end of the box. **d**-**e**, PCA featuring AN and H9 primed hPSC, naïve hPSC, hTSC, EVT, and STB RNA-seq samples using the expression data of all genes (**d**) and hTSC EGs (**e**)[2]. **f**, Expression of hTSC EGs in the CTB and STB of 10 d.p.f. human embryo[3]. Two-tailed Wilcoxon Rank Sum Test was used for statistical analysis. '**' indicates a p-value<0.01. The exact p-value is 1.4e-08. Boxplot presents the 25th, median, and 75th quartiles, the whiskers extend 1.5 of interquartile ranges. Cell numbers: CTB (30); STB (30). **g**, Expression of hTSC EGs in the CTB, EVT, and STB of 12 d.p.f. human embryo[3]. Two-tailed Wilcoxon Rank Sum Test was used for statistical analysis. '***' indicates a p-value<0.001. The exact p-values are 7.5e-11 (CTB vs. EVT), 5.5e-08 (CTB vs. STB). Boxplot presents the 25th, median, and 75th quartiles, the whiskers extend 1.5 of interquartile ranges. Cell numbers: CTB (24; EVT (4); STB (29). **h**, Expression of hTSC EGs in AN and H9 hTSC, EVT, and STB RNA-seq data[2]. The hTSC samples represent four independent samples across two genetic

backgrounds, and the EVT and STB samples each represent two independent samples across two genetic backgrounds. Boxplot presents the 25th, median, and 75th quartiles, the whiskers extend 1.5 of interquartile ranges. Two-tailed Wilcoxon Rank Sum Test was used for statistical analysis. ‘**’ and ‘***’ indicate p-values <0.01 and <0.001. The exact p-values from left to right are 0.001, <2.22e-16, 0.0098, and <2.22e-16.

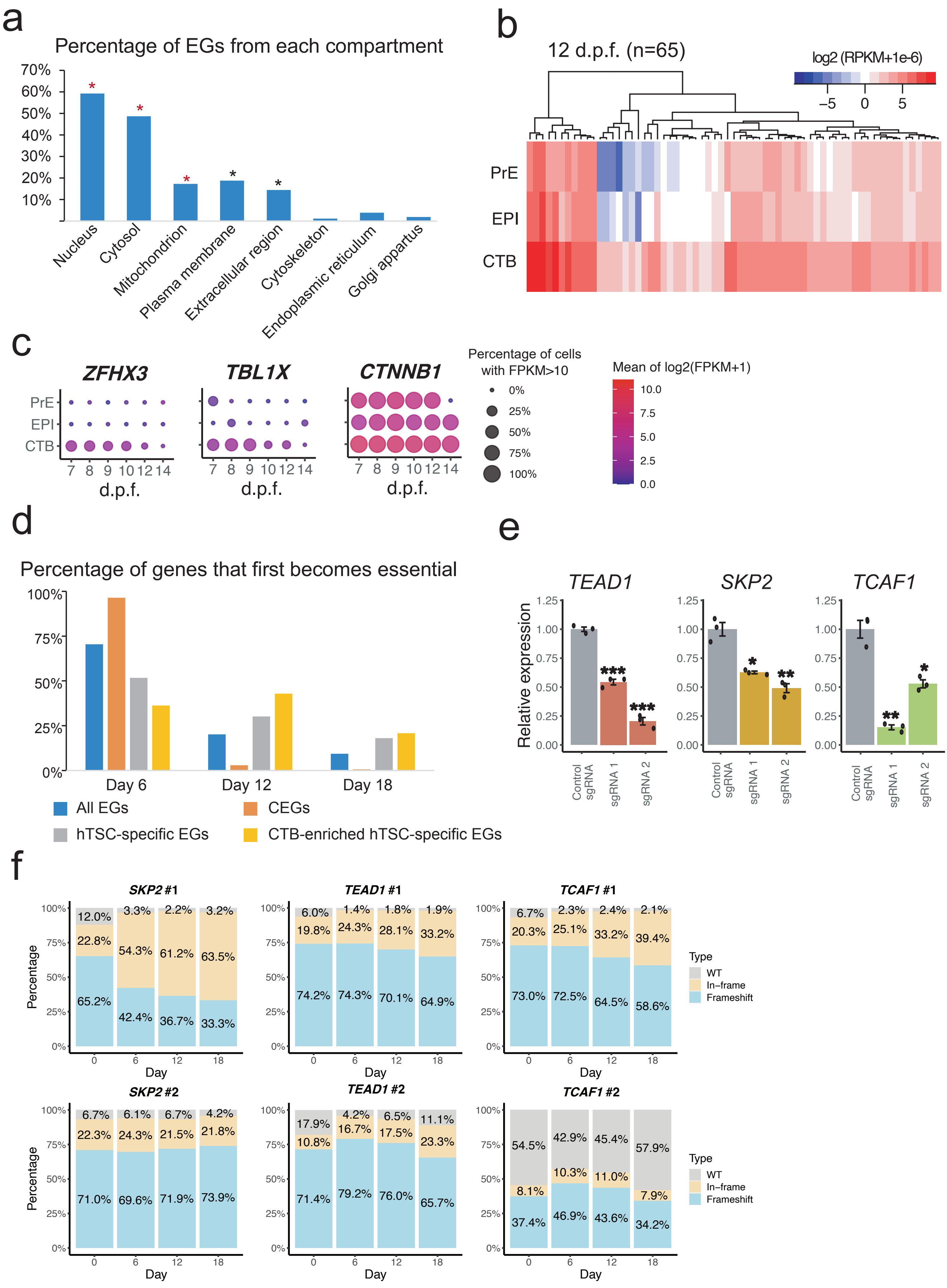

a
Percentage of EGs from each compartment
70%
60%
50%
40%
30%
20%
10%
Nucleus
Cytosol
Mitochondrion
Plasma membrane
Extracellular region
Cytoskeleton
Endoplasmic reticulum
Golgi appartus
b
12 d.p.f. (n=65)
log2 (RPKM+1e-6)
−5
0
5
PrE
EPI
CTB
c
ZFHX3
TBL1X
CTNNB1
PrE
EPI
CTB
7 8 9 10 12 14
d.p.f.
Percentage of cells with FPKM>10
0%
25%
50%
75%
100%
Mean of log2(FPKM+1)
10.0
7.5
5.0
2.5
0.0
d
Percentage of genes that first becomes essential
100%
75%
50%
25%
0%
Day 6
Day 12
Day 18
All EGs
CEGs
hTSC-specific EGs
CTB-enriched hTSC-specific EGs
e
TEAD1
SKP2
TCAF1
Relative expression
1.25
1.00
0.75
0.50
0.25
0.00
Control sgRNA
sgRNA 1
sgRNA 2
f
SKP2 #1
12.0% 3.3% 2.2% 3.2%
22.8% 54.3% 61.2% 63.5%
65.2% 42.4% 36.7% 33.3%
TEAD1 #1
6.0% 1.4% 1.8% 1.9%
19.8% 24.3% 28.1% 33.2%
74.2% 74.3% 70.1% 64.9%
TCAF1 #1
6.7% 2.3% 2.4% 2.1%
20.3% 25.1% 33.2% 39.4%
73.0% 72.5% 64.5% 58.6%
SKP2 #2
6.7% 6.1% 6.7% 4.2%
22.3% 24.3% 21.5% 21.8%
71.0% 69.6% 71.9% 73.9%
TEAD1 #2
17.9% 4.2% 6.5% 11.1%
10.8% 16.7% 17.5% 23.3%
71.4% 79.2% 76.0% 65.7%
TCAF1 #2
54.5% 42.9% 45.4% 57.9%
8.1% 10.3% 11.0% 7.9%
37.4% 46.9% 43.6% 34.2%
Percentage
100%
75%
50%
25%
0%
0 6 12 18
Day
Type
WT
In−frame
Frameshift

Supplementary Fig. 4: Identification and characterization of hTSC-specific EGs. A, The percentage of hTSC-specific EGs localized to each subcellular compartment among all hTSC-specific Egs[4]. If the same gene is predicted to be localized in multiple compartments, it is counted multiple times. The red and blue "*" indicate that relative to their representations in the entire library, the hTSC-specific EGs are significantly more or less enriched in that specific compartment, respectively (one-sided hypergeometric test, p-value<0.05). The exact p-values can be found in the Source Data file. **b,** Heatmap showing the expression of 65 CTB-enriched hTSC-specific EGs in the CTB, EPI, and PrE of published 12 d.p.f. human embryo scRNA-seq data[3]. **c,** Dot plot showing the expression of selected CTB-enriched hTSC-specific EGs in the CTB, EPI, and PrE of published human embryo scRNA-seq data[3]. **d**, The percentage of all hTSC EGs, core EGs that are also essential in our screen[1], hTSC-specific EGs, and CTB-enriched hTSC-specific EGs that first become essential at each timepoint. **e**, Quantitative gene expression analysis for *SKP2, TEAD1, and TCAF1* in hTSCs derived from H9 naïve hPSCs transduced with control sgRNA as well as sgRNAs targeting *SKP2, TEAD1,* and *TCAF1*. Error bars indicate ±1 SE of three technical replicates. The center of the error bar indicates the mean. Two-tailed student's t test was used for statistical analysis. '*' indicates a p-value<0.05, '**' indicates a p-value<0.01, and '***' indicates a p-value<0.001. The exact p-values from left to right are 0.000174819, 0.000138908, 0.020187053, 0.003199302, 0.005227982, and 0.013576238. **f**, The percentage of NGS reads that

contain WT, in-frame mutations, and frameshift mutations in H9 hTSCs transduced with sgRNAs targeting *SKP2*, *TEAD1*, and *TCAF1*.

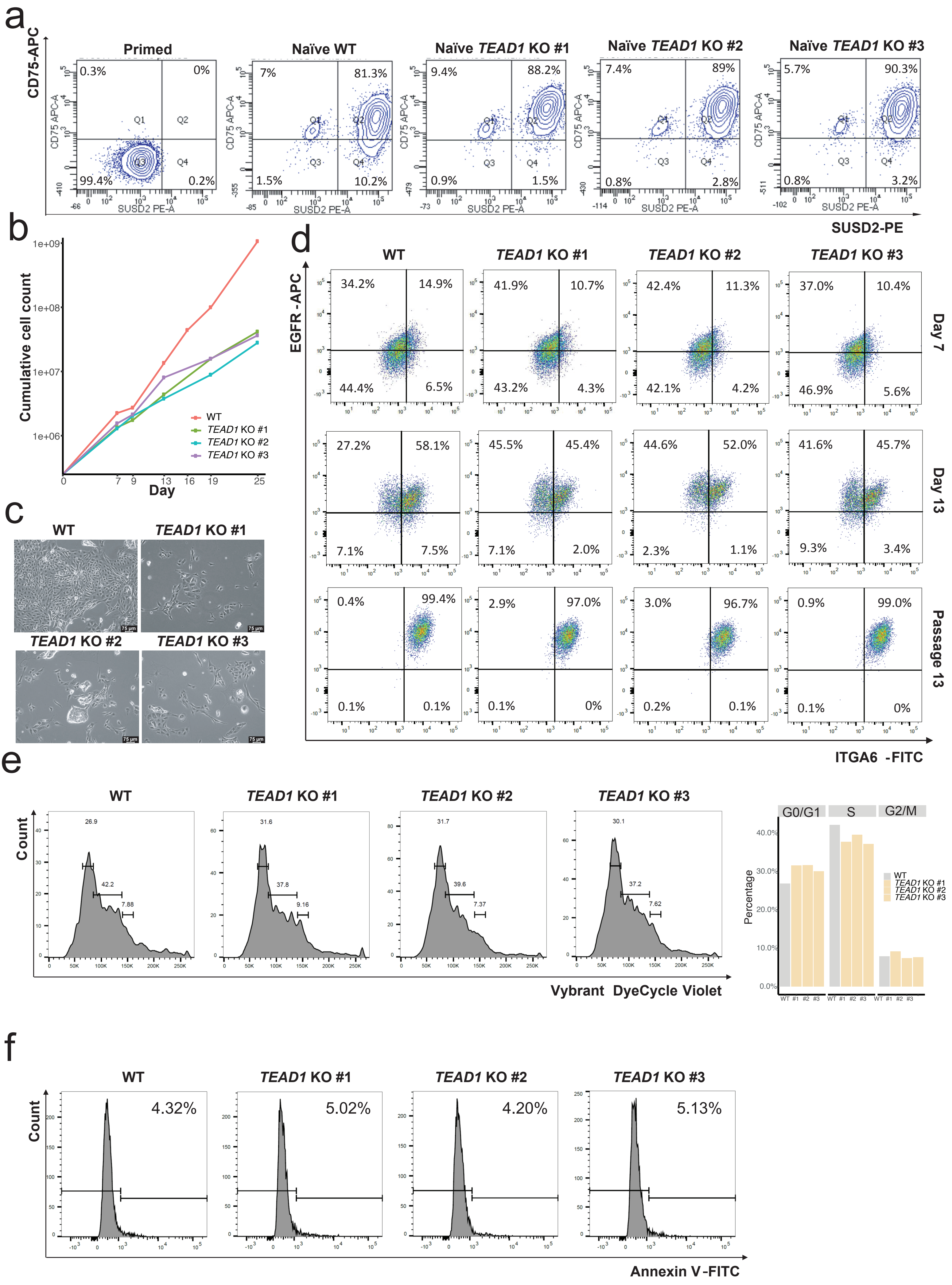
a
CD75-APC
Primed
Naïve WT
Naïve TEAD1 KO #1
Naïve TEAD1 KO #2
Naïve TEAD1 KO #3
0.3%
0%
99.4%
0.2%
7%
81.3%
1.5%
10.2%
9.4%
88.2%
0.9%
1.5%
7.4%
89%
0.8%
2.8%
5.7%
90.3%
0.8%
3.2%
CD75 APC-A
SUSD2 PE-A
SUSD2-PE
b
Cumulative cell count
Day
WT
TEAD1 KO #1
TEAD1 KO #2
TEAD1 KO #3
c
WT
TEAD1 KO #1
TEAD1 KO #2
TEAD1 KO #3
d
EGFR -APC
WT
TEAD1 KO #1
TEAD1 KO #2
TEAD1 KO #3
Day 7
Day 13
Passage 13
34.2%
14.9%
44.4%
6.5%
41.9%
10.7%
43.2%
4.3%
42.4%
11.3%
42.1%
4.2%
37.0%
10.4%
46.9%
5.6%
27.2%
58.1%
7.1%
7.5%
45.5%
45.4%
7.1%
2.0%
44.6%
52.0%
2.3%
1.1%
41.6%
45.7%
9.3%
3.4%
0.4%
99.4%
0.1%
0.1%
2.9%
97.0%
0.1%
0%
3.0%
96.7%
0.2%
0.1%
0.9%
99.0%
0.1%
0%
ITGA6 -FITC
e
Count
WT
TEAD1 KO #1
TEAD1 KO #2
TEAD1 KO #3
26.9
42.2
7.88
31.6
37.8
9.16
31.7
39.6
7.37
30.1
37.2
7.62
Vybrant DyeCycle Violet
G0/G1
S
G2/M
Percentage
WT
TEAD1 KO #1
TEAD1 KO #2
TEAD1 KO #3
f
Count
WT
TEAD1 KO #1
TEAD1 KO #2
TEAD1 KO #3
4.32%
5.02%
4.20%
5.13%
Annexin V-FITC

Supplementary Fig. 5: Investigating the role of TEAD1 in hTSC specification and maintenance. a, Flow cytometry analysis for naïve hPSC markers CD75 and SUSD2 in three independent *TEAD1* KO clones. **b**, Cumulative live cell counts of the first 25 days of hTSC derivation. This experiment represents the mean of two biological replicates. **c**, Phase contrast images of cells at day 17 of hTSC derivation. The scale bars indicate 75 μm. The images are representative of two independent experiments. **d**, Flow cytometry analysis for hTSC markers ITGA6 and EGFR during hTSC derivation. **e**, Cell cycle analysis on WT and *TEAD1* KO hTSCs. **f**, Flow cytometry analysis for apoptosis marker Annexin V in WT and *TEAD1* KO hTSCs.

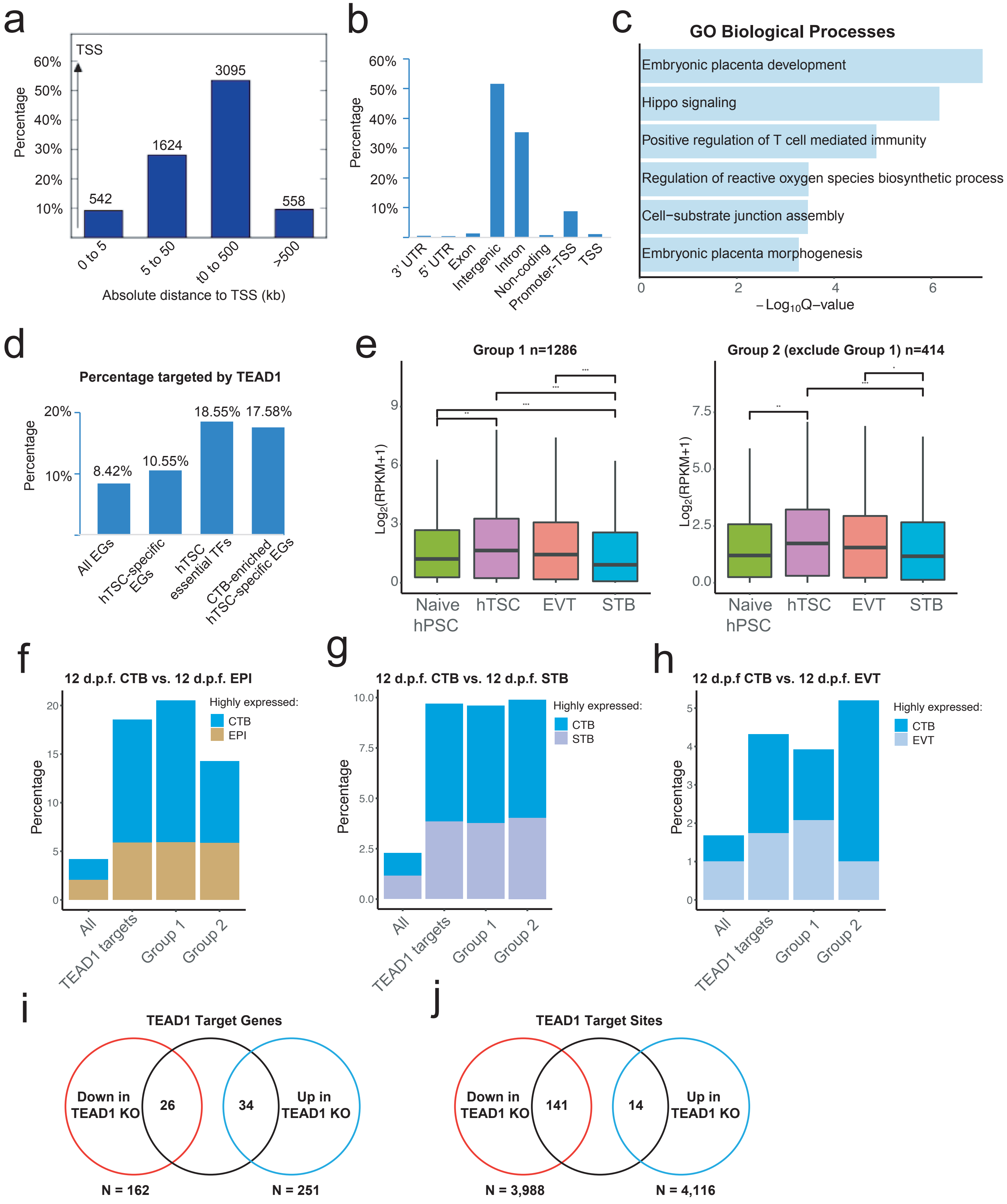

a
TSS
Percentage
60%
50%
40%
30%
20%
10%
542
1624
3095
558
0 to 5
5 to 50
t0 to 500
>500
Absolute distance to TSS (kb)
b
Percentage
3' UTR
5' UTR
Exon
Intergenic
Intron
Non-coding
Promoter-TSS
TSS
c
GO Biological Processes
Embryonic placenta development
Hippo signaling
Positive regulation of T cell mediated immunity
Regulation of reactive oxygen species biosynthetic process
Cell−substrate junction assembly
Embryonic placenta morphogenesis
−Log10Q−value
d
Percentage targeted by TEAD1
Percentage
8.42%
10.55%
18.55%
17.58%
All EGs
hTSC-specific EGs
hTSC essential TFs
CTB-enriched hTSC-specific EGs
e
Group 1 n=1286
Group 2 (exclude Group 1) n=414
Log2(RPKM+1)
Naive hPSC
hTSC
EVT
STB
f
12 d.p.f. CTB vs. 12 d.p.f. EPI
Highly expressed:
CTB
EPI
Percentage
All
TEAD1 targets
Group 1
Group 2
g
12 d.p.f. CTB vs. 12 d.p.f. STB
Highly expressed:
CTB
STB
h
12 d.p.f CTB vs. 12 d.p.f. EVT
Highly expressed:
CTB
EVT
i
TEAD1 Target Genes
Down in TEAD1 KO
26
34
Up in TEAD1 KO
N = 162
N = 251
j
TEAD1 Target Sites
Down in TEAD1 KO
141
14
Up in TEAD1 KO
N = 3,988
N = 4,116

Supplementary Fig. 6: Investigation of TEAD1 targets in hTSCs. a, Distribution of transcriptional start site (TSS) distance to the TEAD1 CUT&Tag peaks. **b**, Genomic distribution of TEAD1 CUT&Tag peaks. **c**, Top GO biological processes significantly enriched among TEAD1 CUT&Tag peaks and their p-values. **d**, Percentage of hTSC EGs (n=2,139), hTSC-specific EGs (n=872), hTSC essential transcription factors (n=124), and CTB-enriched hTSC-specific EGs (n=91) targeted by TEAD1. **e**, Expression of group 1 and group 2 (excluding those already in group 1) TEAD1 target genes in naïve hPSCs, hTSCs, EVTs, and STBs[2]. Two-tailed Wilcoxon Rank Sum Test was used for statistical analysis. '*' indicates a p-value<0.05, '**' indicates a p-value<0.01, and '***' indicates a p-value<0.001. The exact group 1 p-values are 0.0015 (naïve hPSC vs. hTSC), 0.1 (naïve hPSC vs. EVT), 5.5e-05 (naïve hPSC vs. STB), 0.19 (hTSC vs. EVT), and 4e-11 (hTSC vs. STB). The exact group 2 p-values are 0.0046 (naïve hPSC vs. hTSC), 0.21 (naïve hPSC vs. EVT), 0.32 (naïve hPSC vs. STB), 0.15 (hTSC vs. EVT), 0.00045 (hTSC vs. STB), and 0.033 (EVT vs. STB). Boxplot presents the 25th, median, and 75th quartiles, the whiskers extend 1.5 of interquartile ranges. **f-h**, Percentage of all genes, all TEAD1 target genes, group 1 TEAD1 target genes, and group 2 (excluding those already in group 1) TEAD1 target genes that are significantly up- or down regulated in published 12 d.p.f. CTB vs. 12 d.p.f. EPI (**f**), 12 d.p.f. CTB vs 12 d.p.f. STB (**g**), and 12 d.p.f. CTB vs 12 d.p.f. EVT (**h**)[3]. **i**, Overlap of Group 1 and 2 TEAD1 target genes with genes significantly upregulated or downregulated in *TEAD1* KO hTSCs relative to WT hTSCs. **j**, Overlap of Group 1 and 2 TEAD1 targets

with DARs significantly more open or closed in *TEAD1* KO hTSCs relative to WT hTSCs.

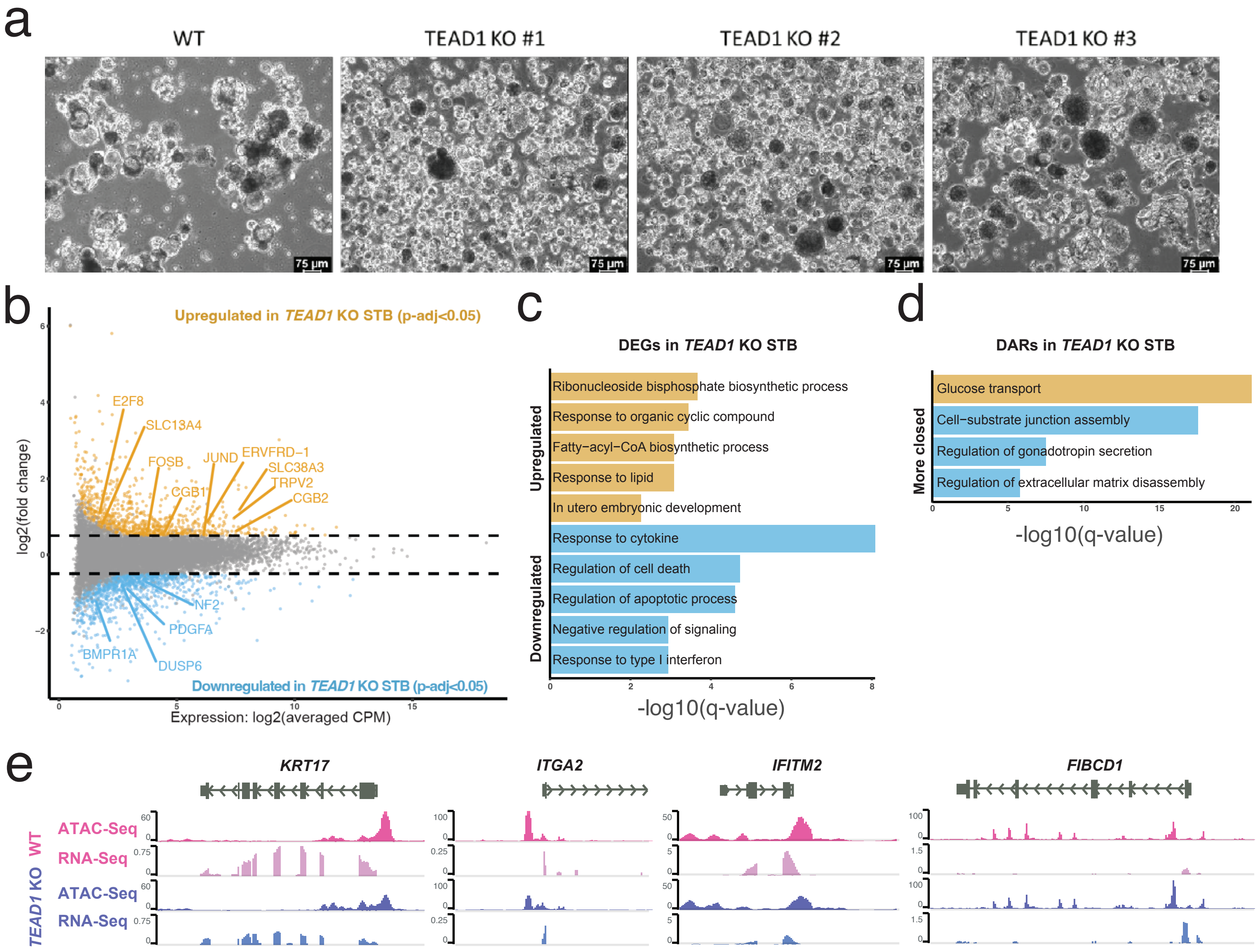
a
WT
TEAD1 KO #1
TEAD1 KO #2
TEAD1 KO #3
75 µm
b
Upregulated in TEAD1 KO STB (p-adj<0.05)
E2F8
SLC13A4
FOSB
JUND
ERVFRD−1
SLC38A3
TRPV2
CGB1
CGB2
NF2
PDGFA
BMPR1A
DUSP6
Downregulated in TEAD1 KO STB (p-adj<0.05)
log2(fold change)
Expression: log2(averaged CPM)
c
DEGs in TEAD1 KO STB
Upregulated
Ribonucleoside bisphosphate biosynthetic process
Response to organic cyclic compound
Fatty−acyl−CoA biosynthetic process
Response to lipid
In utero embryonic development
Downregulated
Response to cytokine
Regulation of cell death
Regulation of apoptotic process
Negative regulation of signaling
Response to type I interferon
-log10(q-value)
d
DARs in TEAD1 KO STB
More closed
Glucose transport
Cell−substrate junction assembly
Regulation of gonadotropin secretion
Regulation of extracellular matrix disassembly
-log10(q-value)
e
KRT17
ITGA2
IFITM2
FIBCD1
WT
ATAC-Seq
RNA-Seq
TEAD1 KO
ATAC-Seq
RNA-Seq

Supplementary Fig. 7: Investigating the role of TEAD1 in STB differentiation. a, Phase contrast images of H9 WT and *TEAD1* KO STBs. The scale bars indicate 75 μm. The images are representative of two independent experiments. **b**, Scatter plot showing the differential gene expression analysis between H9 WT and *TEAD1* KO STBs. WT represents two RNA-seq samples, and *TEAD1* KO represents two RNA-seq samples each from three independent clones. **c**, Selected GO biological processes that are enriched among DEGs significantly upregulated or downregulated in *TEAD1* KO STBs relative to WT STBs. **d**, Selected GO biological processes that are enriched among DARs significantly more open (yellow) or closed in *TEAD1* KO STBs relative to WT STBs. **e**, WT and *TEAD1* KO STB RNA-seq and ATAC-seq data shown in the vicinity of selected genes.

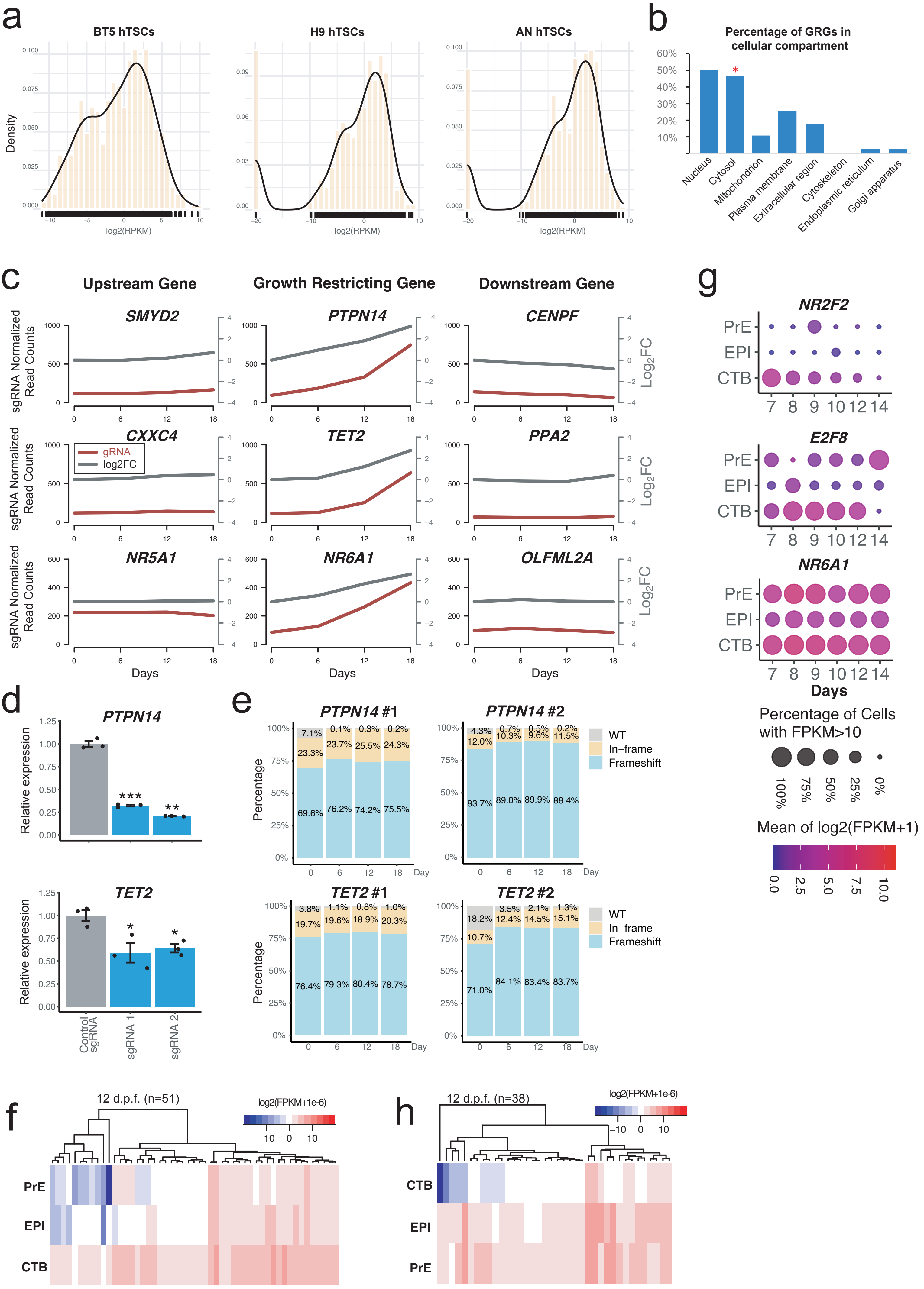
a
BT5 hTSCs
H9 hTSCs
AN hTSCs
Density
log2(RPKM)
b
Percentage of GRGs in cellular compartment
60%
50%
40%
30%
20%
10%
Nucleus
Cytosol
Mitochondrion
Plasma membrane
Extracellular region
Cytoskeleton
Endoplasmic reticulum
Golgi apparatus
c
Upstream Gene
Growth Restricting Gene
Downstream Gene
SMYD2
PTPN14
CENPF
CXXC4
TET2
PPA2
NR5A1
NR6A1
OLFML2A
sgRNA Normalized Read Counts
Log2FC
gRNA
log2FC
Days
d
PTPN14
TET2
Relative expression
Control sgRNA
sgRNA 1
sgRNA 2
e
PTPN14 #1
PTPN14 #2
TET2 #1
TET2 #2
Percentage
Day
WT
In−frame
Frameshift
7.1% 0.1% 0.3% 0.2%
23.3% 23.7% 25.5% 24.3%
69.6% 76.2% 74.2% 75.5%
4.3% 0.7% 0.5% 0.2%
12.0% 10.3% 9.6% 11.5%
83.7% 89.0% 89.9% 88.4%
3.8% 1.1% 0.8% 1.0%
19.7% 19.6% 18.9% 20.3%
76.4% 79.3% 80.4% 78.7%
18.2% 3.5% 2.1% 1.3%
10.7% 12.4% 14.5% 15.1%
71.0% 84.1% 83.4% 83.7%
f
12 d.p.f. (n=51)
log2(FPKM+1e-6)
PrE
EPI
CTB
g
NR2F2
E2F8
NR6A1
PrE
EPI
CTB
Days
Percentage of Cells with FPKM>10
100% 75% 50% 25% 0%
Mean of log2(FPKM+1)
0.0 2.5 5.0 7.5 10.0
h
12 d.p.f. (n=38)
log2(FPKM+1e-6)
CTB
EPI
PrE

Supplementary Fig. 8: Identification and characterization of hTSC GRGs. a, The mRNA expression distribution of all hTSC GRGs in published BT5, H9, and AN hTSC RNA-seq data[2]. Note that the expression distribution of all hTSC EGs is similar regardless of genetic background. **b**, The percentage of GRGs localized to each subcellular compartment among all GRGs[4]. If the same gene is predicted to be localized in multiple compartments, it is counted multiple times. The red "*" indicate that relative to their representations in the entire library, the GRGs are significantly more enriched in that specific compartment (one-sided hypergeometric test, p-value<0.05). The exact p-values can be found in the Source Data file. **c**, The mean of sgRNA normalized read counts and Log2 fold changes of selected hTSC GRGs and their neighboring up- and downstream genes over time. The results are representative of two independently transduced screening experiments. **d**, Quantitative gene expression analysis for *PTPN14* and *TET2* in H9 hTSCs transduced with control sgRNA as well as sgRNAs targeting *PTPN14* and *TET2*. Error bars indicate ±1 SE of three technical replicates. The center of the error bar indicates the mean. Two-tailed student's t test was used for statistical analysis. '*' indicates a p-value<0.05, '**' indicates a p-value<0.01, and '***' indicates a p-value<0.001. The exact p-values from left to right are 0.000926346, 0.001503466, 0.041293211, and 0.011697001. **e**, The percentage of NGS reads that contain WT, in-frame mutations, and frameshift mutations in H9 hTSCs transduced with sgRNAs targeting *PTPN14* and *TET2*. **f**, Heatmap showing the expression of 51 CTB-enriched GRGs in the CTB, EPI, and PrE of published 12 d.p.f. human embryo

scRNA-seq data[3]. **g**, Dot plot showing the expression of selected CTB-enriched GRGs in the CTB, EPI, and PrE of published human embryo scRNA-seq data[3]. **h**, Heatmap showing the expression of 38 CTB-depleted GRGs in the CTB, EPI, and PrE of published 12 d.p.f. human embryo scRNA-seq data[3].

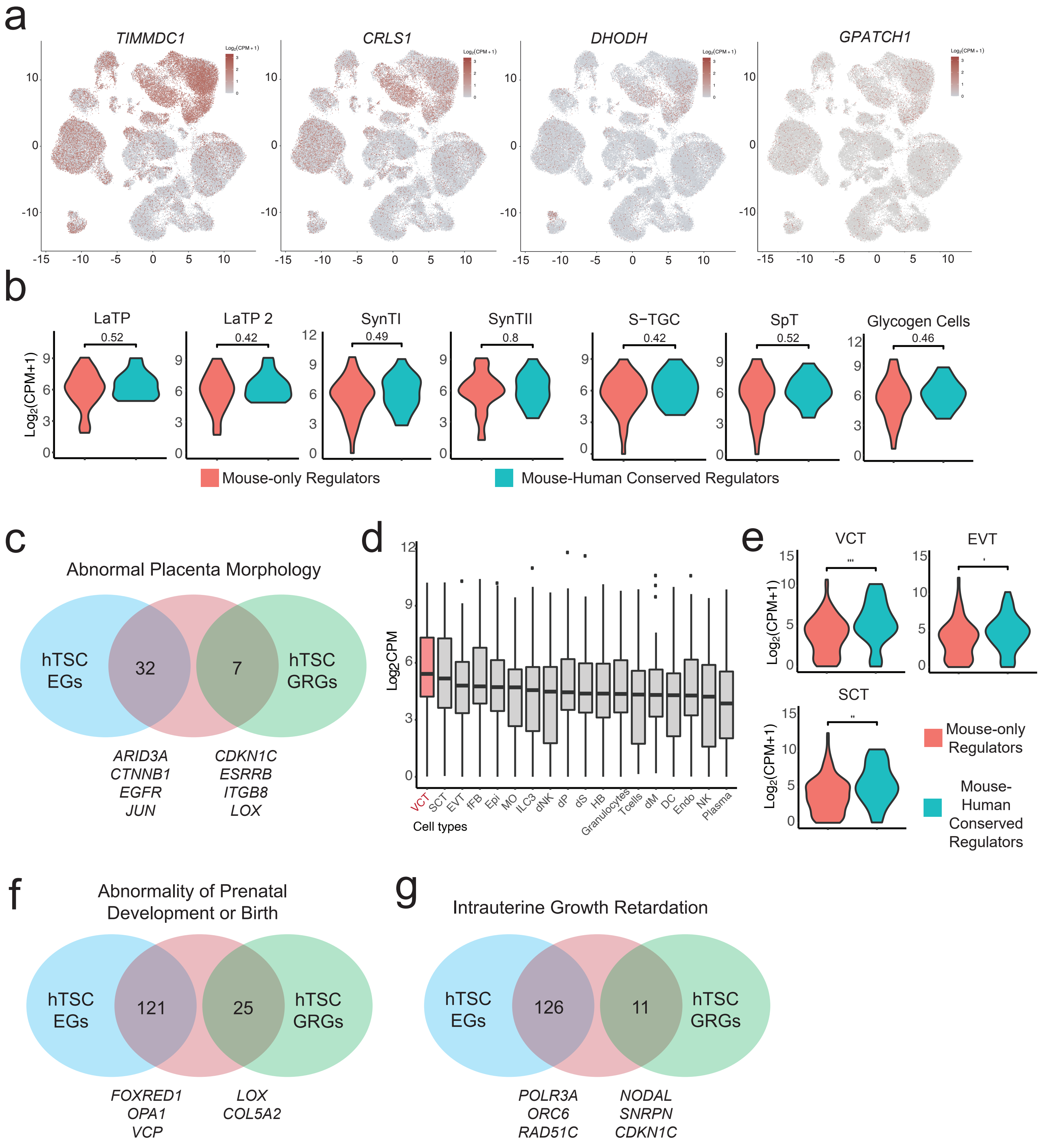
a
TIMMDC1
CRLS1
DHODH
GPATCH1
Log2(CPM+1)
b
LaTP
LaTP 2
SynTI
SynTII
S−TGC
SpT
Glycogen Cells
0.52
0.42
0.49
0.8
0.42
0.52
0.46
Log2(CPM+1)
Mouse-only Regulators
Mouse-Human Conserved Regulators
c
Abnormal Placenta Morphology
hTSC EGs
32
7
hTSC GRGs
ARID3A
CTNNB1
EGFR
JUN
CDKN1C
ESRRB
ITGB8
LOX
d
Log2CPM
VCT
SCT
EVT
fFB
Epi
MO
ILC3
dNK
dP
dS
HB
Granulocytes
Tcells
dM
DC
Endo
NK
Plasma
Cell types
e
VCT
EVT
SCT
Log2(CPM+1)
Mouse-only Regulators
Mouse-Human Conserved Regulators
f
Abnormality of Prenatal Development or Birth
hTSC EGs
121
25
hTSC GRGs
FOXRED1
OPA1
VCP
LOX
COL5A2
g
Intrauterine Growth Retardation
hTSC EGs
126
11
hTSC GRGs
POLR3A
ORC6
RAD51C
NODAL
SNRPN
CDKN1C

Supplementary Fig. 9: Analysis of hTSC EGs and GRGs implicated in mouse placentation and pregnancy-related diseases. a, Expression of selected mouse-human conserved placental regulators (Fig. 6a) in the human maternal-fetal interface[5]. **b**, Expression of genes required for mouse placentation that do not (n=41) and do (n=20) overlap with hTSC EGs/GRGs in mouse placenta snRNA-seq dataset[6]. Two-tailed Wilcoxon Rank Sum Test was used for statistical analysis. The exact p-values are indicated in the figures. Cell numbers: LaTP (786); LaTP 2(1,127); SynTI (2,906); SynTII (822); S-TGC (929); SpT (1,345); Glycogen Cells (2,373). LaTP: labyrinth trophoblast progenitors; SynT: syncytiotrophoblasts; S-TGC: sinusoidal trophoblast giant cells; SpT: spongiotrophoblast. **c**, Overlap of hTSC EGs/GRGs and genes associated with mouse Abnormal Placenta Morphology according to the Mammalian Phenotype Ontology database[7]. **d**, Expression of genes associated with mouse Abnormal Placenta Morphology and overlap with hTSC EGs/GRGs (n=39) in different cell types found in human maternal-fetal interface scRNA-seq dataset[5]. Cell types were ranked based on mean gene expression. Boxplot presents the 25th, median, and 75th quartiles, the whiskers extend 1.5 of interquartile ranges, and the dots are outside values >1.5 times and <3 times the interquartile range beyond either end of the box. **e**, Expression of genes associated with mouse Abnormal Placenta Morphology that do not (n=149) and do (n=39) overlap with hTSC EGs/GRGs in the VCTs, EVTs, and SCTs of human maternal-fetal interface scRNA-seq dataset[5]. Two-tailed Wilcoxon Rank Sum Test was used for statistical analysis. '*' indicates a p-value<0.05, '**' indicates a p-value<0.01, and

‘***’ indicates a p-value<0.001. The exact p-values are 0.00079 (VCT), 0.047 (EVT), and 0.0026 (SCT). Cell numbers: VCT (9,479); EVT (3,626); SCT (1,261). **f,** Overlap of hTSC EGs/GRGs and genes associated with abnormality of prenatal development or birth according to the human phenotype ontology database[8]. The EGs include preeclampsia-associated *FOXRED1, OPA1,* and *VCP*[9-11]. The GRGs include *LOX and COL5A2*, which were associated with preeclampsia[12] and preterm birth[13], respectively. **g**, Overlap of hTSC EGs/GRGs and genes associated with intrauterine growth retardation according to the human phenotype ontology database[8]. The EGs include *POLR3A*[14], *ORC6*[15], and *RAD51C*[16], while the GRGs include *NODAL*[17], *SNRPN*[18], *and CDKN1C*[19,20].

REFERENCES

1. Hart, T. *et al.* Evaluation and Design of Genome-Wide CRISPR/SpCas9 Knockout Screens. *G3: Genes|Genomes|Genetics* **7**, 2719-2727 (2017).
2. Dong, C. *et al.* Derivation of trophoblast stem cells from naive human pluripotent stem cells. *Elife* **9**(2020).
3. Xiang, L. *et al.* A developmental landscape of 3D-cultured human pre-gastrulation embryos. *Nature* **577**, 537-542 (2020).
4. Binder, J.X. *et al.* COMPARTMENTS: unification and visualization of protein subcellular localization evidence. *Database* **2014**(2014).
5. Vento-Tormo, R. *et al.* Single-cell reconstruction of the early maternal–fetal interface in humans. *Nature* **563**, 347-353 (2018).
6. Marsh, B. & Blelloch, R. Single nuclei RNA-seq of mouse placental labyrinth development. *Elife* **9**(2020).
7. Smith, C.L. & Eppig, J.T. The mammalian phenotype ontology: enabling robust annotation and comparative analysis. *Wiley Interdiscip Rev Syst Biol Med* **1**, 390-399 (2009).
8. Köhler, S. *et al.* Expansion of the Human Phenotype Ontology (HPO) knowledge base and resources. *Nucleic Acids Research* **47**, D1018-D1027 (2018).
9. Wang, H. *et al.* MiR-195 modulates oxidative stress-induced apoptosis and mitochondrial energy production in human trophoblasts via flavin adenine dinucleotide-dependent oxidoreductase domain-containing protein 1 and pyruvate dehydrogenase phosphatase regulatory subunit. *J Hypertens* **36**, 306-318 (2018).
10. Vishnyakova, P.A. *et al.* Mitochondrial role in adaptive response to stress conditions in preeclampsia. *Sci Rep* **6**, 32410 (2016).
11. Ozsoy, A.Z. *et al.* Altered expression of p97/Valosin containing protein and impaired autophagy in preeclamptic human placenta. *Placenta* **67**, 45-53 (2018).
12. Xu, X.H. *et al.* Downregulation of lysyl oxidase and lysyl oxidase-like protein 2 suppressed the migration and invasion of trophoblasts by activating the TGF-β/collagen pathway in preeclampsia. *Exp Mol Med* **51**, 1-12 (2019).
13. Anum, E.A., Hill, L.D., Pandya, A. & Strauss, J.F., 3rd. Connective tissue and related disorders and preterm birth: clues to genes contributing to prematurity. *Placenta* **30**, 207-15 (2009).
14. Wambach, J.A. *et al.* Bi-allelic POLR3A Loss-of-Function Variants Cause Autosomal-Recessive Wiedemann-Rautenstrauch Syndrome. *Am J Hum Genet* **103**, 968-975 (2018).
15. Shalev, S.A., Khayat, M., Etty, D.S. & Elpeleg, O. Further insight into the phenotype associated with a mutation in the ORC6 gene, causing Meier-Gorlin syndrome 3. *Am J Med Genet A* **167a**, 607-11 (2015).
16. Jacquinet, A. *et al.* Expanding the FANCO/RAD51C associated phenotype: Cleft lip and palate and lobar holoprosencephaly, two rare findings in Fanconi anemia. *Eur J Med Genet* **61**, 257-261 (2018).
17. Thulluru, H.K. *et al.* Maternal Nodal inversely affects NODAL and STOX1 expression in the fetal placenta. *Front Genet* **4**, 170 (2013).
18. Diplas, A.I. *et al.* Differential expression of imprinted genes in normal and IUGR human placentas. *Epigenetics* **4**, 235-40 (2009).
19. Suntharalingham, J.P. *et al.* Analysis of CDKN1C in fetal growth restriction and pregnancy loss. *F1000Res* **8**, 90 (2019).

20. Takahashi, S. *et al.* Loss of p57^{KIP2} expression confers resistance to contact inhibition in human androgenetic trophoblast stem cells. *Proceedings of the National Academy of Sciences* **116**, 26606-26613 (2019).
