# Supplementary material for: A genome-wide CRISPR-Cas9 knockout screen identifies essential and growth-restricting genes in human trophoblast stem cells
Source: Nat Commun. 2022 May 10;13:2548. doi: 10.1038/s41467-022-30207-9 (PMC9090837; doi:10.1038/s41467-022-30207-9)
Supplement: Supplementary file 2 — Editorial Assessment Report [file 41467_2022_30207_MOESM2_ESM.pdf]

Contents of this report

1. **Manuscript details**: overview of your manuscript and the editorial team.
2. **Review synthesis**: summary of the reviewer reports provided by the editors.
3. **Editorial recommendation**: personalized evaluation and recommendation from all 3 journals.
4. **Annotated reviewer comments**: the referee reports with comments from the editors.
5. **Open research evaluation**: advice for adhering to best reproducibility practices.

About the editorial process

Because you selected the **Nature Portfolio Guided Open Access** option, your manuscript was assessed for suitability in three of our titles publishing high-quality work across the spectrum of genetics research: ***Nature Genetics***, ***Nature Communications***, **and** ***Communications Biology***. More information about Guided Open Access can be found here.

Collaborative editorial assessment

Your editorial team discussed the manuscript to determine its suitability for the Nature Portfolio Guided OA pilot. Our assessment of your manuscript takes into account several factors, including whether the work meets the **technical standard** of the Nature Portfolio and whether the findings are of **immediate significance** to the readership of at least one of the participating journals in the Nature Portfolio Guided Open Access genetics cluster.

Peer review

Experts were asked to evaluate the following aspects of your manuscript:

- **Novelty** in comparison to prior publications;
- **Likely audience** of researchers in terms of broad fields of study and size;
- **Potential impact** of the study on the immediate or wider research field;
- **Evidence** for the claims and whether additional experiments or analyses could feasibly strengthen the evidence;
- **Methodological detail** and whether the manuscript is reproducible as written;
- Appropriateness of the **literature review**.

Editorial evaluation of reviews

Your editorial team discussed the potential suitability of your manuscript for each of the participating journals. They then discussed the revisions necessary in order for the work to be published, keeping each journal's specific editorial criteria in mind.

Journals in the Nature portfolio will support authors wishing to transfer their reviews and (where reviewers agree) the reviewers' identities to journals outside of Springer Nature.
If you have any questions about review portability, please contact our editorial office at guidedoa@nature.com.

Manuscript details

Tracking number	Submission date	Decision date	Peer review type
GUIDEDOA-21-00258	**Sep 10, 2021**	**Nov 15, 2021**	Single-blind

**Manuscript title**	**Author details**
A genome-wide CRISPR-Cas9 knockout screen identifies essential and growth-restricting genes in human trophoblast stem cells	Thorold Theunissen<br>**Affiliation:** Washington University Medical Center

Editorial assessment team

**Primary editor**	**Wei Li**<br>**Home journal:** *Nature Genetics*<br>**ORCID:** 0000-0002-7885-1775<br>**Email:** wei.li@nature.com
**Other editors consulted**	**Poonam Bheda,** *Nature Communications*, ORCID**:** 0000-0001-6587-9456<br>**George Inglis,** *Communications Biology*, ORCID: 0000-0002-9069-5242
**About your primary editor**	Wei Li obtained her Ph.D. in Genetics and Genomics from Shanghai Institutes for Biological Sciences, Chinese Academy of Sciences in Bin Han's plant genomics and multi-omics research group. For her postdoctoral work, Wei moved to The Rockefeller University and then Weill Cornell Medical College of Cornell University at Anthony A. Sauve's laboratory to study signaling networks in mammalian metabolic diseases such as diabetes. She joined the *Nature Genetics* team in 2017.

Editorial assessment and review synthesis

Editor's summary and assessment	The authors utilized a genome-wide CRISPR-Cas9 knockout screen to systematically identify and characterize essential genes (EGs) and growth-restricting genes (GRGs) in human trophoblast stem cells (hTSCs). They identified 2,139 genes essential for hTSC survival and growth. By cross-referencing the essential genes (EGs) to those of similar screens conducted in other cell types and single cell transcriptome data from early human embryos, they obtained a subset of EGs that is specifically essential to hTSCs and upregulated in the trophectoderm lineage in vivo. These hTSC-specific regulators included known and novel trophoblast regulators, such as ARID3A, GATA2, and TEAD1 (essential), and GCM1, PTPN14, and TET2 (growth-restricting). The transcription factor TEAD1 emerged as a leading candidate for further analysis. Using genetic experiments, they confirmed that *TEAD1* knockout significantly impaired hTSC derivation and proliferation. They also integrated multiple genomic methodologies (ATAC-seq, CUT&Tag, and RNA-seq data) to identify the genome-wide targets of TEAD1, demonstrating that it plays major roles in the specification and maintenance of the human trophoblast lineage.<br><br>While the editors jointly decided to send this manuscript out to review, we recognized that there were existing studies on this topic and that the study design was standard. We appreciate the resource value of these datasets and the identification of TEAD1 as an essential gene in hTSCs, though have some concerns about the functional validation and the biological insights obtained from the analyses.

Editorial synthesis of reviewer reports	For consideration at *Nature Communications*, please include mRNA-seq data for TEAD1-KO trophoblast stem cells (Reviewer 1), further investigate the role of TEAD1 in trophoblast stem cell specification/maintenance and temporal dynamics of differentiation, e.g. into EVT cells (Reviewers 1 and 2), as well as clarify the technical concerns on the analyses done with mixed populations of wild-type and TEAD1-KO cells (Reviewer 2).<br><br>For consideration at *Communications Biology*, we would ask that you incorporate the discussion points and changes to figures requested by Reviewer #1, further evaluate the *TEAD1*-KO cells as outlined by Reviewer #2, and include a dynamic temporal analysis of KO cells (per Reviewer #2), as well as address the lack of other requested experiments as limitations in the text.

Editorial recommendation

Nature Genetics<br><br>Revision not invited	The degree of conceptual advance has not matched the criteria for further consideration at *Nature Genetics*.
Nature Communications<br><br>Major revisions with extension of the work	Based on the collective referee advice, we would suggest addressing all experimental and technical concerns outlined by both reviewers. Please see our detailed guidance below.
Communications Biology<br><br>Major revisions	Given the positive feedback from the reviewers, we would that you focus on the experimental revisions requested by Reviewer #2, as well as incorporate the discussion points and figure edits outlined by Reviewer #1. While we agree that many of the experimental concerns outlined by the reviewers would benefit the manuscript, in several cases (as outlined below) it would be sufficient to discuss the lack of those experiments or datasets as limitations.

Next steps

**Editorial recommendation 1:**	Our top recommendation is to revise and resubmit your manuscript to ***Nature Communications.*** We feel all of the additional experiments required are reasonable and appropriate for a manuscript at *Nature Communications*.
**Editorial recommendation 2:**	You may also choose to revise and resubmit your manuscript to ***Communications Biology.*** This option might be best if the requested experimental revisions are not possible/feasible at this time.
**Note**	As stated on the previous page ***Nature Genetics*** is not inviting a revision at this time. Please keep in mind that the journal will not be able to consider any appeals of their decision through Guided Open Access.

Revision

To follow our recommendation, please upload the revised manuscript files using **the link provided in the decision letter**.

Revision checklist

- ☐ Cover letter, stating to which journal you are submitting
- ☐ Revised manuscript
- ☐ Point-by-point response to reviews
- ☐ Updated Reporting Summary and Editorial Policy Checklist
- ☐ Supplementary materials (if applicable)

Submission elsewhere

If you choose not to follow our recommendations, you can still take the reviewer reports with you.

Option 1: Transfer to another Nature Portfolio journal
Springer Nature provides authors with the ability to transfer a manuscript within the Nature Portfolio, without the author having to upload the manuscript data again. To use this service, **please follow the transfer link provided in the decision letter.** If no link was provided, please contact guidedOA@nature.com.

> *Note that any decision to opt in to In Review at the original journal is not sent to the receiving journal on transfer. You can opt in to In Review at receiving journals that support this service by choosing to modify your manuscript on transfer.*

Option 2: Portable Peer Review option for submission to a journal outside of Nature Portfolio
If you choose to submit your revised manuscript to a journal at another publisher, we can share the reviews with another journal outside of the Nature Portfolio if requested. You will need to request that the receiving journal office contacts us at guidedOA@nature.com. We have included editorial guidance below in the reviewer reports and open research evaluation to aid in revising the manuscript for publication elsewhere.

Annotated reviewer reports

The editors have included some additional comments on specific points raised by the reviewers below, to clarify requirements for publication in the recommended journal(s). However, please note that all points should be addressed in a revision, even if an editor has not specifically commented on them.

Reviewer #1 information	
**Expertise**	epigenomics, stem cell biology
**Editor's comments**	This reviewer points out a few limitations of the study design and provides suggestions for improvement. This reviewer also highlights other aspects of the presentation that need to be improved.

Reviewer #1 comments	
**Section**	**Annotated Reviewer Comments**
**Remarks to the Author: Overall significance**	Dong et al. identified genes required for self-renewal of human TS cells (EGs) and genes that restrict their growth (GRGs) using comprehensive CRISPR-Cas9 screening system and comparison with published datasets. They focused on TEAD1, which has not been previously reported to function in human trophoblasts, and demonstrated its involvement in self-renewal by analyzing KO cell lines. It was also shown that TEAD1 functions as a human TS cell-specific transcription factor by integrated analysis with genomic binding sites and open chromatin regions.<br><br>For GRGs, they demonstrated the function of several genes (*PTPN14*, *TET2*) in the suppression of proliferation using KO in hTS cells. Furthermore, comparison with data from other lineages of human embryo suggested the involvement of the identified genes in the trophoblast lineage.<br><br>Finally, the identified genes were compared with the mouse gene set to discuss interspecies conservation between humans and mice.<br><br>This study is suggestive for a better understanding of the genes that define human trophoblast, which is important for human placental development. The experimental procedures, selection of previous research data and analysis methods are also appropriate. It would be more impactful if they could find that TEAD1 is associated not only with the proliferation of cultured human TS cells, but also with the pathogenesis of certain diseases.<br><br>The results are original and significant, and will they be of interest to others in the community and the wider field.

**Remarks to the Author: Impact**	For the above reasons, I recommend *Nature Communications* of this paper, after major revisions.
**Remarks to the Author: Strength of the claims**	Specific comments are as follows.<br><br>1. The authors showed TEAD1 target genes using CUT&TAG and ATAC-seq data. Can the authors include mRNA-seq data of TEAD1-KO TSCs? It would be more representative and accurate to combine transcriptome profiling, CUT&TAG, and ATAC-seq data.<br><br>**This point would be necessary for consideration at *Nature Communications*.**<br><br>**This point would not be necessary for consideration at *Communications Biology* (but should be mentioned as a limitation).**<br><br>2. Has there been any reported association between TEAD1 and pregnancy-related diseases? If not, it would be nice to have a text that discusses the role of TEAD1 *in vivo* or some results using human tissues.<br><br>3. Given that *TEAD1* is highly expressed in EVT, its function in EVT cells or its involvement in the process of differentiation into EVT should be verified using human TS cells.<br><br>**As both Reviewers 1 and 2 requested additional data for TEAD1 function in EVT cells, this experiment is critical for consideration at *Nature Communications*.**<br><br>**This point would not be necessary for consideration at *Communications Biology* (but should be mentioned as a limitation).**<br><br>4. The authors have confirmed the phenotype of PTPN14 and TET2-KO TS cells. For TET2, there is a description in the text that explains the difference in its role from that of mice, but there is no description mentioning the function of PTPN14, which needs to be added.<br><br>5. Figure 4f, It would be nice if the genes in Fig4b, d (*TGFBI* and *PTPN14*) are also included.<br><br>6. Figure 5c, the selection criteria for the seven genes should be clearly stated. Also, there should be a statement in the text that mentions each gene whose expression is shown in the figure.<br><br>7. Fig 5, The reader may also be interested in human-specific genes that are not shared with the 61 genes in mice. It would be nice to have a figure comparing the

	expression levels of such genes in mice and humans, as shown in Figure 5d.<br><br>Minor comments<br>1. Line 62: Although the authors replicated the CRISPR screening with independent transduction, they used only one hTSC line, BT5. I think this experiment is not biologically replicated but technically replicated.<br><br>2. Refs 1 and 26 are identical.<br><br>3. The origin of hTSCs used for the CUT&TAG experiment is unclear. Were they derived from hPSCs?<br><br>4. In Figure 5, the authors considered only genes identified in Ref 71. There are many more genes reported to be essential for mouse placental development. It would be better to use databases, such as http://www.informatics.jax.org/, to identify mouse placental regulators.<br><br>5. Line 834: CDKN1C is already reported to negatively regulate hTSC growth (Takahashi et al., PNAS 2019, https://doi.org/10.1073/pnas.1916019116). This paper should be cited.<br><br>6. Line 190-191: Please specify and briefly why it is important to study GRGs in TSCs. Are there any reports about the association between GRGs and placenta diseases?
**Remarks to the Author: Reproducibility**	There are no particular problems.

Reviewer #2 information

**Expertise**	stem cell biology, CRISPR-Cas9 screen
**Editor's comments**	This reviewer has major concerns regarding the functional characterization and other aspects of the experiments and/or analyses, which should be thoroughly addressed for further consideration.

Reviewer #2 comments

**Section**	**Annotated Reviewer Comments**
**Remarks to the Author: Overall significance**	In this manuscript Dong et al perform a genome-wide CRISPR screen to uncover new trophoblast stem cell regulators. The mechanisms that regulate the specification, maintenance, and differentiation of the human trophoblast remain very poorly characterised. The authors take advantage of the recently developed conditions to culture human trophoblast stem cells in vitro, to identify novel molecular regulators, in particular essential and growth-restricting genes. As far as this reviewer knows, this is the first systematic study focused on the identification of trophoblast stem cell regulators. As such, it will be very interesting for those studying human embryogenesis, and specifically placenta development. The results of the screen represent a powerful resource for the community, and the data generated can be used in the future to identify novel regulators and characterise their mechanism of action. The manuscript is very clearly written and the data is well presented. The weakest section of the manuscript is the functional characterisation, and I think a number of additional studies need to be performed before the manuscript is accepted for publication, as outlined below.
**Remarks to the Author: Impact**	I think this paper is suitable for *Nature Communications*. The data generated will be useful for the field.
**Remarks to the Author: Strength of the claims**	Both in the results and the discussion the authors conclude that they have identified new regulators of specification, maintenance and differentiation of the trophoblast. This is a major overstatement. In detail, there are the following issues with the data presented:<br><br>1. In figure 2h and f the authors transfected human trophoblast stem cells with specific guide RNAs and analysed cell numbers as a readout. This reviewer understands that single clones were not picked, given that the efficiency of gene depletion is relatively low (Supplementary Figure 4e). Therefore, the culture most likely consists of a mix of WT and KO cells. Given that they are

analysing potentially essential genes, the results are likely to depend on when exactly the cells were analysed. Upon prolonged passaging one would expect WT cells to take over, as in the screen. Is this the phenotype they observe in this experimental setting?

Please clarify this point, for further consideration at *Nature Communications* or *Communications Biology*.

2. To understand how TEAD1 regulates trophoblast stem cell maintenance, the experiments shown in Figure 2h and f are definitely not enough. The authors should generate a line of TEAD1 KO trophoblast stem cells, and analyse them in detail: what are the levels of proliferation, apoptosis and differentiation? In other words, why is TEAD1 supposedly essential for trophoblast stem cell maintenance?

The evidence supporting TEAD1 role in regulating trophoblast stem cell maintenance are required to be extended for further consideration at *Nature Communications*.

At a minimum, this point should be addressed as a limitation for further consideration at *Communications Biology*, and the functional role of TEAD1 carefully qualified throughout the text.

3. The authors also mention briefly the potential role of TEAD1 during trophoblast stem cell differentiation. This is not really addressed in the experiments presented. Using the line of TEAD1 KO trophoblast stem cells, the authors could trigger differentiation to the syncytiotrophoblast and the extravillous trophoblast lineages, and compare the behaviour of WT and TEAD1 KO trophoblast stem cells.

As both Reviewers 1 and 2 requested additional data for TEAD1 function in EVT cells, this experiment is critical for consideration at *Nature Communications*.

This point would not be required for further consideration at *Communications Biology*.

4. The experiments presented in Figures 2i-m are very difficult to interpret. Rather than deleting TEAD1 in trophoblast stem cells, the authors decide to perform this experiment in human embryonic stem cells, and then trigger their differentiation towards the trophoblast lineage. These experiments are therefore assessing specification, rather than maintenance. However, key controls and experiments are missing. Figure 2l shows a decrease in proliferation by day 13. What is exactly happening at this stage? Are the WT human embryonic stem cells already converted into the trophoblast lineage? And the TEAD1 KO cells? Are the dynamics of the conversion the same? Are the

	TEAD1 KO cells blocked at some point during the transition? Figure 2m shows a clear decrease in proliferation in the KO clones. What is the identity of these cells? Figure 2n shows an increase in the syncytiotrophoblast markers SDC1 and CGB for two of the KO clones, but multi-nucleated cells with the clear phenotype of syncytiotrophoblast are not observed in figure 2m. In summary, the dynamics of trophoblast differentiation and the identity of the cells generated should be investigated in detail in the KO clones. Clone 3 is behaving quite differently. The specificity of the phenotypes could be validated by performing a rescue experiment (re-expression of TEAD1).<br><br>**These points would be required for further consideration at *Nature Communications* or *Communications Biology*.**<br><br>5. Figure 4d: in this case, as for figures 2h and f, the dynamics of the process are very important, as the cultures most likely consist of a mix of WT and KO cells. Do the KO cells eventually take over and eliminate the WT cells? Showing a dynamic temporal analysis would be very informative.<br><br>**This point would be required for further consideration at *Nature Communications* or *Communications Biology*.**

Open research evaluation

Data availability
**Data availability statement**
Thank you for including a Data Availability statement. We noticed that not all datasets reported in the paper are included in this statement. The data availability statement must make the conditions of access to the “minimum dataset” that are necessary to interpret, verify and extend the research in the article, transparent to readers. More information about our data availability policy can be found here: https://www.nature.com/nature-portfolio/editorial-policies/reporting-standards#availability-of-data<br><br>See here for more information about formatting your Data Availability Statement: http://www.springernature.com/gp/authors/research-data-policy/data-availability-statements/12330880
**Mandatory data deposition**
For all sequencing data, submission to a community-endorsed, public repository is mandatory for publication in a Nature Portfolio journal and is best practice for publication in any venue. Accession numbers must be provided in the paper. Examples of appropriate public repositories are listed below:<br>-Gene Expression Omnibus (Microarray or RNA sequencing data)<br>-Sequence Read Archive (high-throughput sequence data)<br>-The European Nucleotide Archive (ENA)<br><br>More information on mandatory data deposition policies at the Nature Portfolio can be found at http://www.nature.com/authors/policies/availability.html#data<br><br>Please visit https://www.springernature.com/gp/authors/research-data-policy/repositories/12327124 for a list of approved repositories for each mandatory data type.
**Other data requests**
Springer Nature strongly supports data sharing and believes that all datasets on which the conclusions of the paper rely should be available to readers. We encourage authors to ensure that their datasets are either deposited in publicly available repositories (where available and appropriate) or presented in the main manuscript or additional supporting files whenever possible.<br><br>Please see Springer Nature’s information on recommended repositories: https://www.springernature.com/gp/authors/research-data-policy/repositories/12327124<br><br>We recommend that you make your electron microscopy dataset available via the Electron Microscopy Data Bank: https://wwwdev.ebi.ac.uk/emdb/

All source data underlying the graphs and charts presented in the main figures must be made available as Supplementary Data (in Excel or text format) or via a generalist repository (eg, Figshare or Dryad). This is mandatory for publication in a Nature Portfolio journal, but is also best practice for publication in any venue.

Data availability: This journal strongly supports public availability of data and custom code associated with the paper in a persistent repository where they can be freely and enduringly accessed or as a supplementary data file when no appropriate repository is available. If data and code can only be shared on request, please explain why in your data Availability Statement, and also in the correspondence with your editor. For more information, please refer to https://www.nature.com/nature-research/editorial-policies/reporting-standards#availability-of-data

Please ensure that datasets deposited in public repositories are now publicly accessible, and that accession codes or DOI are provided in the "Data Availability" section. As long as these datasets are not public, we cannot proceed with the acceptance of your paper. For data that have been obtained from publicly available sources, please provide a URL and the specific data product name in the data availability statement. Data with a DOI should be further cited in the methods reference section.

Ethics

Manuscripts that report experiments involving the use of human embryos and gametes, human embryonic stem cells and related materials, and clinical applications of stem cells must include confirmation that all experiments were performed in accordance with relevant guidelines and regulations.

Please ensure your manuscript includes an ethics statement identifying the institutional and/or licensing committees approving the experiments and describing any relevant details. The ethics statement must also confirm that informed consent was obtained from all recipients and/or donors of cells or tissues, where necessary, and describe the conditions of donation of materials for research, such as human embryos or gametes.

Reporting & reproducibility

Please include the full, uncropped blot/gel images as Supplementary Figure(s) and cite the new Supplementary Figure(s) in the main manuscript text.

Reproducibility: Please state in the legends how many times each experiment was repeated independently with similar results. This is needed for all experiments, but is particularly important wherever results from representative experiments (such as micrographs) are shown. If space in the legends is limiting, this information can be included in a section titled “Statistics and Reproducibility” in the methods section.

Legends requiring revision:
Please note that this information is missing in the legends of figures 2h, k, m; 3b; 4d (phase contrast

images).

Gels and Blots: Quantitative comparisons between samples on different gels/blots are discouraged; if this is unavoidable, the figure legend must state that the samples derive from the same experiment and that gels/blots were processed in parallel.

Vertically sliced images that juxtapose lanes that were non-adjacent in the gel must have a clear separation or a black line delineating the boundary between the gels. Loading controls (e.g. GAPDH, actin) must be run on the same blot.

Sample processing controls run on different gels must be identified as such in the figure legends, and distinctly from loading controls.

All blots and gels must be accompanied by the locations of molecular weight/size markers. Blots should be cropped such that at least one marker position is present.

Please also supply uncropped and unprocessed scans of the most important blots in the Source Data file or as a supplementary figure in the Supplementary Information. This should be cited once in the Methods section. For an example of presentation of full scan blots, see the Source Data file of https://www.nature.com/articles/s41467-020-16984-1#Sec35 and for more information, please refer to https://www.nature.com/nature-research/editorial-policies/image-integrity

Panels requiring revision:
1. Please note that molecular weight markers are missing for figure 2k (in the main figure).

Micrographs: Please ensure that all micrographs include a scale bar and this scale bar is defined on the panels or in the figure legends.

Flow cytometry data: Please provide a Supplementary Figure to graphically account for all FACS sequential gating/sorting strategies, or provide gating/sorting strategies in-figure. If the former, please be sure to indicate, in the Supplementary Figure legend, which gating panel(s) correspond to which FACS data panel(s) in the manuscript figures. (For an example, please see https://www.nature.com/articles/ncomms15067#supplementary-information).

Data presentation: Please ensure that data presented in a plot, chart or other visual representation format shows data distribution clearly (e.g. dot plots, box-and-whisker plots). When using bar charts, please overlay the corresponding data points (as dot plots) whenever possible and always for n ≤ 10. (Please see the following editorial for the rationale behind this request and an example https://www.nature.com/articles/s41551-017-0079).

Statistics: Wherever statistics have been derived (e.g. error bars, box plots, statistical significance) the legend needs to provide and define the n number (i.e. the sample size used to derive statistics) as a precise value (not a range), using the wording “n=X biologically independent samples/animals/cells/independent experiments/n= X cells examined over Y independent experiments”

etc. as applicable.<br><br>**Legends requiring revision:**<br>1. Please note that this information is missing in the legends of figure 3i and supplementary figures 3c, f-h; 6c.<br>2. Although ‘n’ is provided, please describe the nature of entity for ‘n’ in the legends of figures 1e; 4b.
Statistics such as error bars, significance and p values cannot be derived from n<3 and must be removed from all such cases.<br>Please note that this should be rectified for figure 2l and supplementary figure 3a.
We strongly discourage deriving statistics from technical replicates, unless there is a clear scientific justification for why providing this information is important. Conflating technical and biological variability, e.g., by pooling technical replicate samples across independent experiments is strongly discouraged. (For examples of expected description of statistics in figure legends, please see the following https://www.nature.com/articles/s41467-019-11636-5 or https://www.nature.com/articles/s41467-019-11510-4).
All error bars need to be defined in the legends (e.g. SD, SEM) together with a measure of centre (e.g. mean, median). For example, the legends should state something along the lines of “Data are presented as mean values +/- SEM” as appropriate.<br>All box plots need to be defined in the legends in terms of minima, maxima, centre, bounds of box and whiskers and percentile.<br><br>**Legends requiring revision:**<br>1. Please note that the box plots need to be defined in terms of minima, maxima, centre, bounds of box and whiskers and percentile in the legends of figures 3i; 5b and supplementary figures 3c, f-h; 5e.
The figure legends must indicate the statistical test used. Where appropriate, please indicate in the figure legends whether the statistical tests were one-sided or two-sided and whether adjustments were made for multiple comparisons.<br>For null hypothesis testing, please indicate the test statistic (e.g. F, t, r) with confidence intervals, effect sizes, degrees of freedom and P values noted.<br>Please provide the test results (e.g. P values) as exact values whenever possible and with confidence intervals noted.<br><br>**Legends requiring revision:**<br>1. Please indicate the statistical test used for data analysis and where appropriate, please specify whether it was one-sided or two-sided and whether adjustments were made for multiple comparisons, in the legends of figures 2f, g, n; 3c; 4a, d; supplementary figures 4e; 6d and tables 2; 4.<br>2. Please note that the information on whether the statistical test used was one-sided or two-sided, where appropriate, is missing in the legends of figure 5d and supplementary figures 3f-h; 4a; 5e; 6b.<br>3. Please note that the exact p value should be provided, when possible, in the legends of figures 2f, g,

n; 4a, d; 5d and supplementary figures 3f-h; 4a, e; 5e; 6b, d.
**Other notes**
We have included as an attachment to the decision letter a version of your Reporting Summary with a few notes. This is mainly for your information, but we hope it is helpful when preparing your revised manuscript. If you decide to resubmit the manuscript for further consideration, please be sure to include an updated Reporting Summary.
