# Supplementary material for: A genome-wide CRISPR-Cas9 knockout screen identifies essential and growth-restricting genes in human trophoblast stem cells
Source: Nat Commun. 2022 May 10;13:2548. doi: 10.1038/s41467-022-30207-9 (PMC9090837; doi:10.1038/s41467-022-30207-9)
Supplement: Supplementary file 3 — List of Supplementary Data [file 41467_2022_30207_MOESM3_ESM.docx]

Supplementary Data 1. hTSC CRISPR screen analysis and list of essential genes.

Supplementary Data 2. Gene Ontology and Pathway terms.

Supplementary Data 3. List of growth-restricting genes.

Supplementary Data 4. Differentially expressed genes between WT and TEAD1 KO hTSC, EVT, and STB.

Supplementary Data 5. Transcription factor binding motifs enriched at TEAD1 binding sites in hTSCs.
