# Supplementary material for: A genome-wide CRISPR-Cas9 knockout screen identifies essential and growth-restricting genes in human trophoblast stem cells
Source: Nat Commun. 2022 May 10;13:2548. doi: 10.1038/s41467-022-30207-9 (PMC9090837; doi:10.1038/s41467-022-30207-9)
Supplement: Supplementary file 8 — Supplementary Data 5 [file 41467_2022_30207_MOESM8_ESM.pdf]

Homer *de novo* Motif Results (homer_peaks/)

[Known Motif Enrichment Results](#)
[Gene Ontology Enrichment Results](#)
If Homer is having trouble matching a motif to a known motif, try copy/pasting the matrix file into [STAMP](#)
More information on motif finding results: [HOMER](#) | [Description of Results](#) | [Tips](#)
Total target sequences = 3565
Total background sequences = 37091
* - possible false positive

Rank	Motif	P-value	log P-pvalue	% of Targets	% of Background	STD(Bg STD)	Best Match/Details	Motif File
1	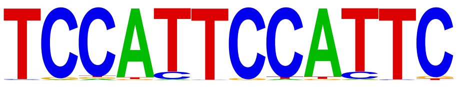	1e-569	-1.312e+03	8.50%	0.05%	49.3bp (71.6bp)	TEAD1(TEAD)/HepG2-TEAD1-ChIP-Seq(Encode)/Homer(0.698) More Information | Similar Motifs Found	motif file (matrix)
2	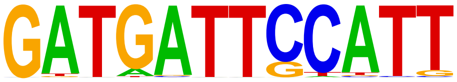	1e-146	-3.372e+02	2.52%	0.02%	55.0bp (15.2bp)	DUX4/MA0468.1/Jaspar(0.733) More Information | Similar Motifs Found	motif file (matrix)
3	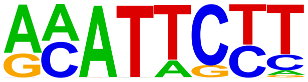	1e-145	-3.351e+02	18.76%	6.11%	54.2bp (71.6bp)	TEAD4(TEA)/Tropoblast-Tead4-ChIP-Seq(GSE37350)/Homer(0.843) More Information | Similar Motifs Found	motif file (matrix)
4	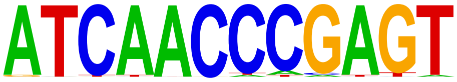	1e-94	-2.184e+02	1.18%	0.00%	57.1bp (0.0bp)	PH0016.1_Cux1_1/Jaspar(0.566) More Information | Similar Motifs Found	motif file (matrix)
5	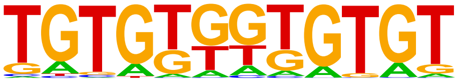	1e-80	-1.845e+02	3.93%	0.45%	54.5bp (136.7bp)	KLF10(Zf)/HEK293-KLF10.GFP-ChIP-Seq(GSE58341)/Homer(0.596) More Information | Similar Motifs Found	motif file (matrix)
6	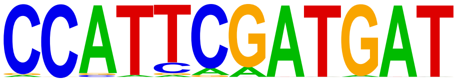	1e-78	-1.796e+02	1.29%	0.01%	48.9bp (0.0bp)	ZNF410/MA0752.1/Jaspar(0.617) More Information | Similar Motifs Found	motif file (matrix)
7	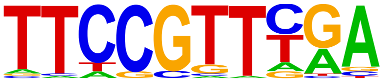	1e-61	-1.426e+02	1.01%	0.01%	59.9bp (52.4bp)	MF0001.1_ETS_class/Jaspar(0.735) More Information | Similar Motifs Found	motif file (matrix)
8	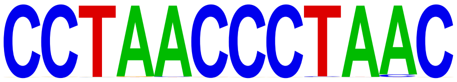	1e-57	-1.328e+02	0.95%	0.01%	51.2bp (47.0bp)	CRX(Homeobox)/Retina-Crx-ChIP-Seq(GSE20012)/Homer(0.587) More Information | Similar Motifs Found	motif file (matrix)
9	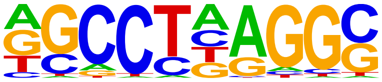	1e-50	-1.161e+02	13.01%	6.14%	52.6bp (62.7bp)	AP-2gamma(AP2)/MCF7-TFAP2C-ChIP-Seq(GSE21234)/Homer(0.934) More Information | Similar Motifs Found	motif file (matrix)
10	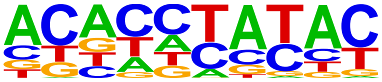	1e-44	-1.036e+02	7.43%	2.76%	58.0bp (100.7bp)	PB0198.1_Zfp128_2/Jaspar(0.627) More Information | Similar Motifs Found	motif file (matrix)
11	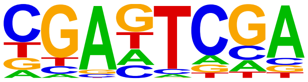	1e-39	-9.089e+01	3.37%	0.75%	60.2bp (70.6bp)	PB0203.1_Zfp691_2/Jaspar(0.653) More Information | Similar Motifs Found	motif file (matrix)
12	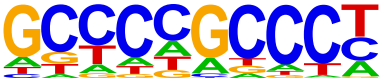	1e-39	-9.026e+01	5.33%	1.74%	54.3bp (55.8bp)	Sp2(Zf)/HEK293-Sp2.eGFP-ChIP-Seq(Encode)/Homer(0.918) More Information | Similar Motifs Found	motif file (matrix)
13	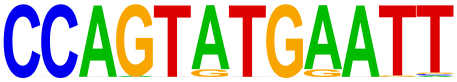	1e-25	-5.876e+01	0.53%	0.01%	55.7bp (32.8bp)	PB0171.1_Sox18_2/Jaspar(0.600) More Information | Similar Motifs Found	motif file (matrix)
14	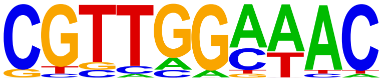	1e-23	-5.480e+01	0.50%	0.01%	53.6bp (25.5bp)	PB0160.1_Rfxdc2_2/Jaspar(0.643) More Information | Similar Motifs Found	motif file (matrix)
15	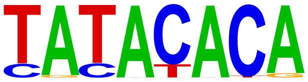	1e-16	-3.856e+01	3.79%	1.67%	55.1bp (106.4bp)	Foxf1(Forkhead)/Lung-Foxf1-ChIP-Seq(GSE77951)/Homer(0.750) More Information | Similar Motifs Found	motif file (matrix)
16		1e-	-3.811e+01	1.26%	0.26%	56.3bp	PB0163.1_Six6_2/Jaspar(0.704)	motif

	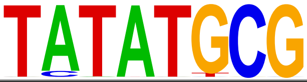	16				(65.8bp)	More Information | Similar Motifs Found	file (matrix)
17	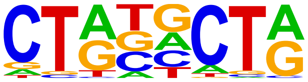	1e-15	-3.528e+01	35.81%	29.55%	54.9bp (69.3bp)	GATA1/MA0035.4/Jaspar(0.647) More Information | Similar Motifs Found	motif file (matrix)
18	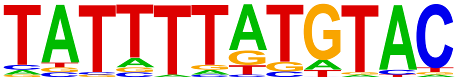	1e-12	-2.979e+01	0.22%	0.00%	54.1bp (31.9bp)	PB0015.1_Foxa2_1/Jaspar(0.652) More Information | Similar Motifs Found	motif file (matrix)
19 *	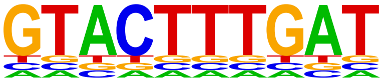	1e-6	-1.586e+01	0.22%	0.02%	57.3bp (46.7bp)	TCF7/MA0769.2/Jaspar(0.803) More Information | Similar Motifs Found	motif file (matrix)
20 *	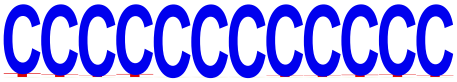	1e-2	-5.511e+00	0.22%	0.07%	55.1bp (62.6bp)	PB0097.1_Zfp281_1/Jaspar(0.916) More Information | Similar Motifs Found	motif file (matrix)
21 *	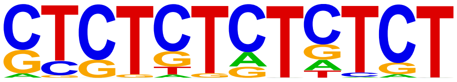	1e0	-1.569e+00	0.45%	0.36%	46.3bp (66.4bp)	PRDM1/MA0508.3/Jaspar(0.646) More Information | Similar Motifs Found	motif file (matrix)
