# Supplementary material for: A genome-wide CRISPR-Cas9 knockout screen identifies essential and growth-restricting genes in human trophoblast stem cells
Source: Nat Commun. 2022 May 10;13:2548. doi: 10.1038/s41467-022-30207-9 (PMC9090837; doi:10.1038/s41467-022-30207-9)
Supplement: Supplementary file 9 — Reporting Summary [file 41467_2022_30207_MOESM9_ESM.pdf]

Corresponding author(s): Bo Zhang, Thorold Theunissen

Last updated by author(s): Apr 8, 2022

Reporting Summary

Nature Research wishes to improve the reproducibility of the work that we publish. This form provides structure for consistency and transparency in reporting. For further information on Nature Research policies, see our Editorial Policies and the Editorial Policy Checklist.

Statistics

For all statistical analyses, confirm that the following items are present in the figure legend, table legend, main text, or Methods section.

n/a	Confirmed	
☐	☒	The exact sample size (*n*) for each experimental group/condition, given as a discrete number and unit of measurement
☐	☒	A statement on whether measurements were taken from distinct samples or whether the same sample was measured repeatedly
☐	☒	The statistical test(s) used AND whether they are one- or two-sided<br>*Only common tests should be described solely by name; describe more complex techniques in the Methods section.*
☒	☐	A description of all covariates tested
☐	☒	A description of any assumptions or corrections, such as tests of normality and adjustment for multiple comparisons
☐	☒	A full description of the statistical parameters including central tendency (e.g. means) or other basic estimates (e.g. regression coefficient) AND variation (e.g. standard deviation) or associated estimates of uncertainty (e.g. confidence intervals)
☐	☒	For null hypothesis testing, the test statistic (e.g. *F*, *t*, *r*) with confidence intervals, effect sizes, degrees of freedom and *P* value noted<br>*Give P values as exact values whenever suitable.*
☒	☐	For Bayesian analysis, information on the choice of priors and Markov chain Monte Carlo settings
☒	☐	For hierarchical and complex designs, identification of the appropriate level for tests and full reporting of outcomes
☐	☒	Estimates of effect sizes (e.g. Cohen's *d*, Pearson's *r*), indicating how they were calculated

Our web collection on statistics for biologists contains articles on many of the points above.

Software and code

Policy information about availability of computer code

Data collection	No software was used for data collection.
Data analysis	HOMER (v4.11.1), AIAP (v1.1), DESeq2 (1.26.0), BAGEL (v.91), MAGeCK (0.5.9.2), deepTools (3.3.1), GREAT (4.0.4), ToppGene Suite (v1), BEDTools Suite (v2.29.2), FACSDiva (v9.0), FlowJo (v10.8.1), STAR (v2.5.4b), ENCODE (v27), RUVSeq (v1.24.0).<br>All custom scripts used in this study are deposited in https://github.com/Zhang-lab/CRISPR/.

For manuscripts utilizing custom algorithms or software that are central to the research but not yet described in published literature, software must be made available to editors and reviewers. We strongly encourage code deposition in a community repository (e.g. GitHub). See the Nature Research guidelines for submitting code & software for further information.

Data

Policy information about availability of data

All manuscripts must include a data availability statement. This statement should provide the following information, where applicable:

- Accession codes, unique identifiers, or web links for publicly available datasets
- A list of figures that have associated raw data
- A description of any restrictions on data availability

The CRISPR screen sequencing data, TEAD1 CUT&Tag data, naïve hPSC ATAC-seq data, and the WT and TEAD1 KO hTSC, EVT, and STB RNA-seq and ATAC-seq data generated in this study are available under the GEO accession number GSE172329 (https://www.ncbi.nlm.nih.gov/geo/query/acc.cgi?acc=GSE172329). The WT primed hPSC, naïve hPSC, hTSC, EVT, and STB RNA-seq data and the WT hTSC ATAC-seq data were retrieved from GSE138762 (https://www.ncbi.nlm.nih.gov/geo/query/acc.cgi?acc=GSE138762); the human embryo scRNA-seq data was retrieved from GSE136447 (https://www.ncbi.nlm.nih.gov/geo/query/acc.cgi?acc=GSE136447); the human maternal-fetal interface scRNA-seq data was retrieved from E-MTAB-6701 (https://www.ebi.ac.uk/arrayexpress/experiments/E-MTAB-6701/) and E-MTAB-6678 (https://www.ebi.ac.uk/arrayexpress/experiments/E-MTAB-6678/); the mouse placenta snRNA-seq data was retrieved from

GSE152248 (https://www.ncbi.nlm.nih.gov/geo/query/acc.cgi?acc=GSE152248); the human reference genome was retrieved from GENCODE v27 (https://www.gencodegenes.org/human/release_27.html). Source data associated with Fig. 2f, g, Fig. 3d, Fig. 4c, Fig. 5d, Fig. 6a, Supplementary Fig. 4a, d, e, f, Supplementary Fig. 5b, Supplementary Fig. 6d, i, j, Supplementary Fig. 8b, d, e, and Supplementary Fig. 9c, f, g are provided with this paper in the Source Data file.

Field-specific reporting

Please select the one below that is the best fit for your research. If you are not sure, read the appropriate sections before making your selection.

☒ Life sciences ☐ Behavioural & social sciences ☐ Ecological, evolutionary & environmental sciences

For a reference copy of the document with all sections, see nature.com/documents/nr-reporting-summary-flat.pdf

Life sciences study design

All studies must disclose on these points even when the disclosure is negative.

Sample size	For CRISPR screen, RNA-seq, and ATAC-seq experiments, two replicates were used per sample; for TEAD1 CUT & Tag experiments, three replicates were used. These sample sizes are chosen because they are sufficient to assess reproducibility for the experiment in question. No statistical method was used to determine the sample size. For all other experiments in this study, no statistical method was used to determine the sample size, and the sample sizes are chosen because they provide sufficient confidence to assess the experimental results.
Data exclusions	No data was excluded.
Replication	To ensure reproducibility, the CRISPR screen and the hTSC derivation experiments were conducted twice independently; the EVT and STB differentiation experiments were conducted at least three times independently. All other results reported in this study contained at least technical replicates. All replications yielded similar results and were successful.
Randomization	No randomized experiment was performed.
Blinding	Blinding was not relevant for our study since no group allocation was performed.

Reporting for specific materials, systems and methods

We require information from authors about some types of materials, experimental systems and methods used in many studies. Here, indicate whether each material, system or method listed is relevant to your study. If you are not sure if a list item applies to your research, read the appropriate section before selecting a response.

Materials & experimental systems

n/a	Involved in the study	
☐	☒	Antibodies
☐	☒	Eukaryotic cell lines
☒	☐	Palaeontology and archaeology
☒	☐	Animals and other organisms
☒	☐	Human research participants
☒	☐	Clinical data
☒	☐	Dual use research of concern

Methods

n/a	Involved in the study	
☐	☒	ChIP-seq
☐	☒	Flow cytometry
☒	☐	MRI-based neuroimaging

Antibodies

Antibodies used	anti-SUSD2-PE, 1:100 (BioLegend, 327406); anti-CD75-eFluor 660, 1:100 (Thermo Fisher, 50-0759-42); anti-ITGA6-FITC, 1:100 (Miltenyi, 130-097-245); anti-EGFR-APC, 1:20 (BioLegend, 352905); anti-Annexin V-FITC, 1:10 (Thermo Fisher, BMS147FI); anti-TEAD1, 1:100 (Cell Signaling Technology, 12292S); anti-rabbit-Alexa 647, 1:500 (Invitrogen, A-31573); guinea pig-anti-rabbit, 1:100 (Antibodies online, ABIN101961); anti-beta-actin, 1:1000 (Cell Signaling Technology, 4970)
Validation	anti-SUSD2-PE: https://www.biolegend.com/en-us/products/pe-anti-human-susd2-antibody-4354?GroupID=GROUP28<br>anti-CD75-eFluor 660: https://www.thermofisher.com/antibody/product/CD75-Antibody-clone-LN-1-Monoclonal/50-0759-42<br>anti-ITGA6-FITC: https://www.miltenyibiotec.com/US-en/products/cd49f-antibody-anti-human-mouse-goh3.html#gref<br>anti-EGFR-APC: https://www.biolegend.com/en-us/products/apc-anti-human-egfr-antibody-7714?GroupID=BLG9533<br>anti-Annexin V-FITC: https://www.thermofisher.com/antibody/product/Annexin-V-Antibody-clone-VAA-33-Monoclonal/BMS147FI<br>anti-TEAD1: https://www.cellsignal.com/products/primary-antibodies/tead1-d9x2l-rabbit-mab/12292<br>anti-rabbit-Alexa 647: https://www.thermofisher.com/antibody/product/Donkey-anti-Rabbit-IgG-H-L-Highly-Cross-Adsorbed-Secondary-Antibody-Polyclonal/A-31573<br>guinea pig-anti-rabbit: https://www.antibodies-online.com/antibody/101961/Guinea+Pig+anti-Rabbit+IgG+Heavy++Light+Chain+antibody+-+Preadsorbed/<br>anti-beta-actin: https://www.cellsignal.com/products/primary-antibodies/b-actin-13e5-rabbit-mab/4970

Eukaryotic cell lines

Policy information about cell lines

Cell line source(s)	The blastocyst-derived BT5 hTSCs were obtained from Dr. William Pastor, Dr. Hiroaki Okae, and Dr. Takahiro Arima (Okae et al., Cell Stem Cell, 2018); H9 primed hPSCs and K562 cells were obtained from WashU GEiC; HEK293T cells were obtained from Dr. Rudolf Jaenisch and the original vendor could not be retrieved.
Authentication	The BT5 hTSCs, K562 cells, and HEK293T cells were not authenticated; the H9 primed hPSCs were authenticated using STR profiling.
Mycoplasma contamination	The cell culture is regularly tested and negative for mycoplasma contamination.
Commonly misidentified lines (See ICLAC register)	None was used in this study.

ChIP-seq

Data deposition

☒ Confirm that both raw and final processed data have been deposited in a public database such as GEO.

☒ Confirm that you have deposited or provided access to graph files (e.g. BED files) for the called peaks.

Data access links<br>*May remain private before publication.*	https://www.ncbi.nlm.nih.gov/geo/query/acc.cgi?acc=GSE172329
Files in database submission	Day0Rep1_S1_L003_R1_001.fastq.gz<br>Day0Rep1_S1_L003_R2_001.fastq.gz<br>Day0Rep2_S2_L003_R1_001.fastq.gz<br>Day0Rep2_S2_L003_R2_001.fastq.gz<br>Day18Rep1_S4_L003_R1_001.fastq.gz<br>Day18Rep1_S4_L003_R2_001.fastq.gz<br>Day18Rep2_S3_L003_R1_001.fastq.gz<br>Day18Rep2_S3_L003_R2_001.fastq.gz<br>Day_0_Rep_1_R1_001.fastq.gz<br>Day_0_Rep_1_R2_001.fastq.gz<br>Day_12_Rep_1_R1_001.fastq.gz<br>Day_12_Rep_1_R2_001.fastq.gz<br>Day_12_Rep_2_R1_001.fastq.gz<br>Day_12_Rep_2_R2_001.fastq.gz<br>Day_6_Rep_1_R1_001.fastq.gz<br>Day_6_Rep_1_R2_001.fastq.gz<br>Day_6_Rep_2_R1_001.fastq.gz<br>Day_6_Rep_2_R2_001.fastq.gz<br>HNKFYDSXY_AACCGAGTTC-GATCTCTGGA_L004_R1.fastq.gz<br>HNKFYDSXY_AACCGAGTTC-GATCTCTGGA_L004_R2.fastq.gz<br>HNKFYDSXY_CGCACGACTG-GGACCAACAG_L004_R1.fastq.gz<br>HNKFYDSXY_CGCACGACTG-GGACCAACAG_L004_R2.fastq.gz<br>HNKFYDSXY_GAGGTTAGAC-AATGTATTGC_L004_R1.fastq.gz<br>HNKFYDSXY_GAGGTTAGAC-AATGTATTGC_L004_R2.fastq.gz<br>WangT_Chen-C1-ATAC_SIC808_CTCCAGGGTA_S4_R1_001.fastq.gz<br>WangT_Chen-C1-ATAC_SIC808_CTCCAGGGTA_S4_R2_001.fastq.gz<br>WangT_Chen-C2-ATAC_SIC809_ACGCTTGGAA_S5_R1_001.fastq.gz<br>WangT_Chen-C2-ATAC_SIC809_ACGCTTGGAA_S5_R2_001.fastq.gz<br>WangT_Chen-E1-ATAC_SIC802_CCTAGCTGGA_S8_R1_001.fastq.gz<br>WangT_Chen-E1-ATAC_SIC802_CCTAGCTGGA_S8_R2_001.fastq.gz<br>WangT_Chen-E2-ATAC_SIC803_CTCCTACAAA_S9_R1_001.fastq.gz<br>WangT_Chen-E2-ATAC_SIC803_CTCCTACAAA_S9_R2_001.fastq.gz<br>filtered_cnt.bedGraph<br>normalized_per_10M_GTAC-92238-1.bigWig<br>normalized_per_10M_GTAC-92238-2.bigWig<br>normalized_per_10M_GTAC-92238-3.bigWig<br>normalized_per_10M_WangT_Chen-C1-ATAC_SIC808_CTCCAGGGTA_S4.bigWig<br>normalized_per_10M_WangT_Chen-C2-ATAC_SIC809_ACGCTTGGAA_S5.bigWig<br>normalized_per_10M_WangT_Chen-E1-ATAC_SIC802_CCTAGCTGGA_S8.bigWig<br>normalized_per_10M_WangT_Chen-E2-ATAC_SIC803_CTCCTACAAA_S9.bigWig
Genome browser session (e.g. UCSC)	http://epigenomegateway.wustl.edu/legacy/?genome=hg38&session=C2kY4DRQZJ&statusId=1990469965

Methodology

Replicates	3 biological replicates
Sequencing depth	rep1/2/3: 2.86x/3.49x/3.14x
Antibodies	anti-TEAD1, 1:100 (Cell Signaling Technology, 12292S); guinea pig-anti-rabbit, 1:100 (Antibodies online, ABIN101961)
Peak calling parameters	bash ATAC_IAP_v1.1.sh -g hg38 -r PE
Data quality	Rep1/2/3:<br>Mapped ratio: 0.53/0.49/0.52<br>Uniquely mapped ratio: 0.19/0.17/0.16<br>RUP ratio: 1.96/2.62/2.63
Software	AIAP (v1.1)

Flow Cytometry

Plots

Confirm that:

☒ The axis labels state the marker and fluorochrome used (e.g. CD4-FITC).

☒ The axis scales are clearly visible. Include numbers along axes only for bottom left plot of group (a 'group' is an analysis of identical markers).

☒ All plots are contour plots with outliers or pseudocolor plots.

☒ A numerical value for number of cells or percentage (with statistics) is provided.

Methodology

Sample preparation	Cells were single-cell dissociated using TrypLE Express and washed once in FACS buffer [PBS supplemented with 5% FBS]. The cells were then resuspended in 100 µL fresh FACS buffer, and incubated with antibodies for 30 minutes on ice. Following antibody incubation, the cells were washed once with FACS buffer, resuspended in fresh FACS buffer, and passed through a cell strainer.
Instrument	Flow cytometry was performed using a BD LSRFortessa X-20
Software	Data was analyzed using the FACSDiva software.
Cell population abundance	10,000 events were acquired per sample during the procedure.
Gating strategy	The live cells were first gated using FSC-A/SSC-A. The singlets were then gated using FSC-A/FSC-W and SSC-A/SSC-W. Unstained cells were then used to determine the positive and negative cell populations.

☒ Tick this box to confirm that a figure exemplifying the gating strategy is provided in the Supplementary Information.
